# Hierarchical multi-timescale structural dynamics of the disordered N-terminal of p53

**DOI:** 10.1038/s41467-026-73145-6

**Published:** 2026-05-21

**Authors:** Dániel Szöllősi, Supriya Pratihar, Dwaipayan Mukhopadhyay, Ashok Kumar Rout, Mookyoung Han, G. Jithender Reddy, Niklas Ebersberger, Stefan Becker, Gábor Nagy, Sarah Rauscher, Donghan Lee, Reinhard Klement, Christian Griesinger, Helmut Grubmüller

**Affiliations:** 1 Max Planck Institute for Multidisciplinary Sciences, Department of Theoretical and Computational Biophysics, Göttingen, Germany; 2 Max Planck Institute for Multidisciplinary Sciences, Department of NMR-Based Structural Biology, Göttingen, Germany; 3 Department of Chemical and Physical Sciences, University of Toronto Mississauga, Mississauga, ON Canada; 4 Department of Physics, University of Toronto, Toronto, ON Canada; 5 Department of Chemistry, University of Toronto, Toronto, ON Canada; 6 Present Address: Columbia University, Department of Biochemistry and Molecular Biophysics, New York, NY USA; 7 Present Address: Institute of Chemistry and Metabolomics, University of Lübeck, Lübeck, Germany; 8 Present Address: NMR Division, Department of Analytical & Structural Chemistry, CSIR-Indian Institute of Chemical Technology, Hyderabad, India; 9 Present Address: Institute of Biochemistry, University of Lübeck, Lübeck, Germany; 10 Present Address: Biopharmaceutical Research Center, Korea Basic Science Institute, Cheongju-Si, South Korea

**Keywords:** Intrinsically disordered proteins, Solution-state NMR, Molecular modelling

## Abstract

Most natively folded proteins exhibit a unique spatial structure, which undergoes functional motions ranging from picoseconds to seconds, governed by a hierarchically ordered, funnel-shaped free energy landscape. Intrinsically disordered proteins (IDPs) lack such a stable native structure, but undergo fast interconversions between many different structures. Accordingly, the underlying free energy landscape is assumed to be rather shallow and unstructured. However, although IDPs represent nearly one-third of the human proteome, their structural dynamics on timescales slower than nanoseconds remain largely elusive. Here we reveal the structural dynamics of the prototypical IDP p53-TAD, also known as the “guardian of the genome”, by combining high-power relaxation dispersion nuclear magnetic resonance spectroscopy with large-scale molecular dynamics simulations. We found a complex hierarchy of structural dynamics on timescales covering over seven orders of magnitude, ranging from fast nanoseconds backbone reorientations, via sub-microsecond helix-formation dynamics involving many structural sub-states and transition times, to transient tertiary structure formation slower than 25 microseconds. These rich structural dynamics are unexpectedly similar to the timescale hierarchy of natively folded proteins, which may be key to the ability of p53-TAD – and possibly of other IDPs – to bind many different partners by folding into different structures.

## Abstract (web summary)

The intrinsically disordered protein p53-TAD exhibits unexpectedly complex structural dynamics between nanoseconds and sub-milliseconds, as revealed by nuclear magnetic resonance spectroscopy and unbiased atomistic simulations.

## Introduction

Folded proteins exhibit complex structural dynamics across a wide range of timescales, covering picoseconds to milliseconds or even longer which often implements or modulates their biochemical function^1–4^. These dynamics are governed by a complex^2,5,6^, funnel-shaped free energy landscape^7^, characterised by a hierarchy of low to high energy barrier “tiers”^8^. Detailed knowledge of the distribution of these energy barriers, the resulting kinetics, and the underlying structural dynamics are therefore key to our understanding of how proteins fold and how they perform their remarkably broad range of biochemical functions on the molecular level. At the opposite end of the structural continuum^9^ lie intrinsically disordered peptides and proteins (IDPs) which, in contrast, lack such a stable native structure^10,11^. Rather, they rapidly interconvert between many different structures, with only transiently formed secondary structure elements^12^ and long-range contacts^13^. Operating at the limits of Anfinsen’s dogma^14^, these fast reconfiguration dynamics^15,16^ gave rise to the general notion that the underlying free energy landscape is rather shallow and unstructured^17,18^ or weakly funnelled^19^.

IDPs pose considerable experimental and computational challenges, however, and hence information on their kinetics and specifically their structural dynamics is still sparse. For example, single-molecule spectroscopy experiments revealed fast (ns) reconfiguration dynamics of several IDPs^16,20,21^. Combining start-stop perturbation experiments with fluorescence correlation spectroscopy measurements enabled probing timescales from picoseconds to seconds^15,20^, yet without revealing atomistic details of the underlying structural dynamics. Kinetic information at fast (ps to ns) timescales is also provided by nuclear magnetic resonance (NMR) relaxation measurements^22,23^, whereas NMR relaxation dispersion (RD) measurements typically probe kinetics at several tens of μs or slower^24^. High-speed atomic force microscopy probes dynamics on the ms timescale with near residue level spatial resolution^25^. Atomistic molecular dynamics (MD) simulations provide more direct structural insights, but typically only quantify sub-μs structural dynamics^26–29^, although extensions to μs timescale have been achieved using Markov state models, e.g., for Aβ42^30^. Deep learning based techniques also aim to predict structural ensembles at reduced computational cost, yet so far without interconversion dynamics or timescale information^31,32^. Thus, while combined fluorescent techniques, in particular Förster resonance energy transfer (FRET) and nanosecond fluorescence correlation spectroscopy (nsFCS) access timescales between 100 ns and 10 μs^20,33^, directly linking the kinetics of IDPs to their structural dynamics at atomistic detail, particularly at these timescales, has not yet been possible.

**Fig. 1:**
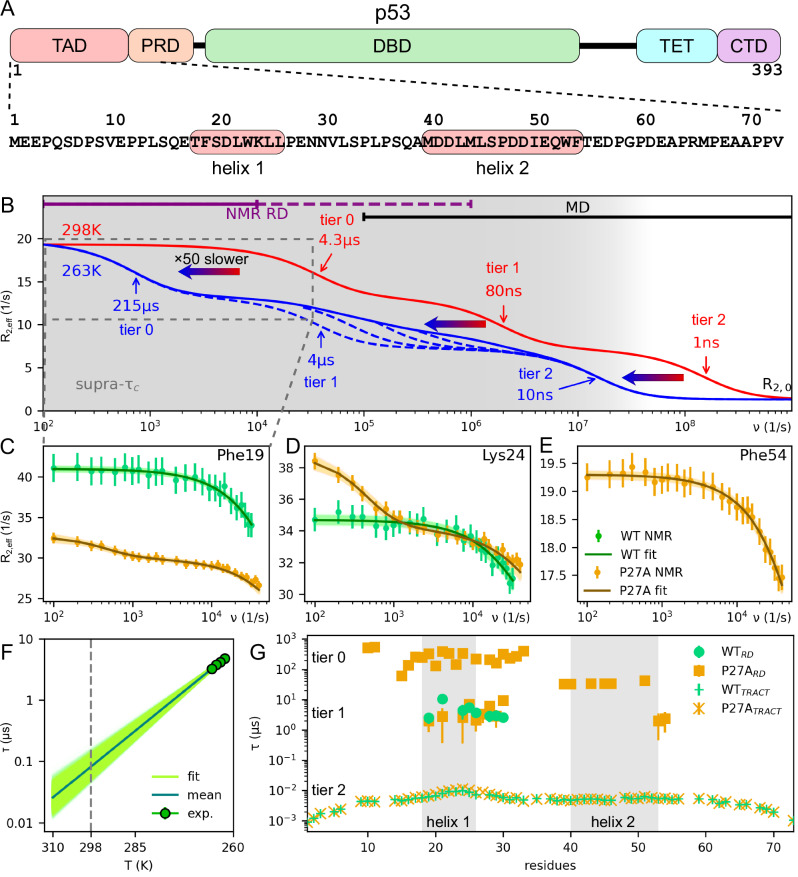
Hierarchical dynamics of p53-TAD across multiple timescales revealed by NMR relaxation dispersion and MD simulations. **A** Domain organisation of full-length p53 protein and sequence of the p53-TAD with highlighted helical segments. **B** Schematic representation of RD profiles revealing three distinct tiers of structural dynamics: slow dynamics (215 μs and 4.3 μs, tier 0), fast dynamics (4 μs and 80 ns, extrapolated using Arrhenius’ equation, tier 1) and molecular tumbling (10 ns and 1 ns, tier 2) at 263 K (blue) and 298 K (red), respectively. Thin arrows indicate timescales from NMR relaxation experiments scaled by (2π)^−1^ for easier interpretation in terms of the well-known Lorentzian function; at the two different temperatures (blue: 263 K, red: 298 K), similar dynamical processes occur at timescales differing by an Arrhenius factor of ca. 50 (horizontal thick arrows, see **F**). Timescales accessible to high-power NMR RD measurement and MD simulations are indicated as purple and black bars; supercooling renders faster timescales accessible to NMR RD (dashed purple bar). The timescale window of the supra-*τ*_c_ dynamics is indicated in grey. **C**–**E** Example mean amide proton (^1^H_N_) CPMG RD profiles measured at 263 K for three different residues (dots) and fitted CPMG curves (posterior distribution shown as lines) including error bars indicating standard deviation of the measurement. Note that no dispersion could be measured for Phe54 of the WT due to spectral overlap. **F** Temperature dependence of relaxation timescales using supercooled NMR ^1^H_N_ *R*_1ρ_ measurements between 262 K and 265 K (mean as green dots, errors are ( ± SD) within symbol size) and Arrhenius extrapolation to 298 K (mean as green line, uncertainty shown by the posterior distribution in light green). **G** Timescales obtained at 263 K for all measured residues by fitting RD data as shown in (**C**–**E**). WT RD profiles (green circles) show a single timescale; several P27A mutant RD profiles show two separate timescales (orange squares), one of which (4 μs) is similar to WT; shown are only those residues for which relaxation dispersion was detected. All residue level tumbling timescales (crosses) are similar for WT and P27A. Error bars are within symbol size; grey shaded areas indicate helix 1 and helix 2.

Here we combined high-power NMR RD measurements and milliseconds atomistic MD simulations to gain access to these 100 ns to 10 μs kinetics and structural dynamics of a prototypic IDP, the intrinsically disordered N-terminal transactivation domain of the tumour protein p53 (p53-TAD, Fig. 1A). Also known as the “guardian of the genome”, p53 regulates the cellular response to genomic damages and thus prevents cancer formation^34^. As one of the most important signalling hubs, p53-TAD adopts different structures upon specific binding to a stunningly large number of proteins^35,36^. X-ray structures of the p53/MDM2 complex^35^, NMR measurements^12^, and MD simulations^26^ suggest that the TAD in solution forms transient helical structures (“helix 1” and “helix 2” in Fig. 1A)^37^. These helices cooperatively engage in binding, can adopt well-folded conformations upon interaction with binding partners^23,35^, and affect the autoinhibition of p53^38,39^. Further, their stability in the unbound state modulates the lifetime of the bound complex^40,41^. The structural dynamics of this prototypical IDP might therefore regulate and accelerate the promiscuous yet selective binding of p53-TAD via conformational selection^37,42,43^, yet much of its kinetics and the underlying structural dynamics are elusive.

## Results

### NMR reveals rich multi-timescale dynamics of p53-TAD

The timescales and dynamics probed by our combined approach are schematically summarised in Fig. 1B. To access so-called supra-τ_c_^44^ dynamics of p53-TAD (residues 1–73) in the low μs range, we have utilised recent advances in high-power RD NMR measurements, in particular, amide proton (^1^H_N_) E-CPMG (CPMG: Carr-Purcell-Meiboom-Gill)^45^ performed at 1.2 GHz (the part of the red curve indicated by the purple bar). To detect even faster protein motions, we also measured under supercooled conditions at 263 K^46^ (dashed purple bar) and found dynamics around 4 μs, e.g., for residues 19, 24, and 54 (green curves in Fig. 1C, D; all measured residues are shown in Supplementary Information Fig. 1 and the extracted timescales in Supplementary Information Table 1). Assuming Arrhenius behaviour and using Bayesian inference to rigorously estimate uncertainties, these supra-*τ*_c_ dynamics are estimated to take place between 66 ns and 100 ns at room temperature (298 K) (Fig. 1B, middle red arrow, Fig. 1F). Here, the rather large uncertainty ( ± 1 standard deviation around the mean) reflects the narrow temperature window between 262 K and 265 K that can be accessed by these measurements. As an additional independent check, we estimated the room temperature timescale using Eyring’s formula^47,48^, yielding similar timescales (Arrhenius: 83.6 ± 18.4 ns; Eyring: 63.2 ± 13.8 ns).

Notably, these supra-*τ*_c_ dynamics are much slower than the nanoseconds reconfiguration dynamics previously observed for IDPs, e.g., by room temperature single-molecule FRET measurements^16,20^. These slower dynamics are therefore governed by markedly higher energy barriers, which we will collectively refer to as ‘tier 1’. Incidentally, loop-closure dynamics of p53-TAD measured by photoinduced electron transfer fluorescence correlation spectroscopy (PET-FCS)^49^ occur at similar timescales, and 50 ns to 100 ns reconfiguration dynamics were also observed for a compact cold shock protein by single molecule FRET/nanosecond FCS^33^. However, because NMR observables are very different from non-local distance fluctuations probed by fluorescence spectroscopy, also the underlying structural dynamics will likely be different. We also observed fast reorientation dynamics (Fig. 1B, right red arrow), governed by lower energy barriers, which we will refer to as ‘tier 2’. Figure 1G shows that these two timescales are measured for many residues (green crosses and circles), suggesting collective structural dynamics involving larger protein segments beyond local fluctuations.

### Tier 1: sub-microsecond supra-*τ*_c_ dynamics of p53-TAD

**Fig. 2:**
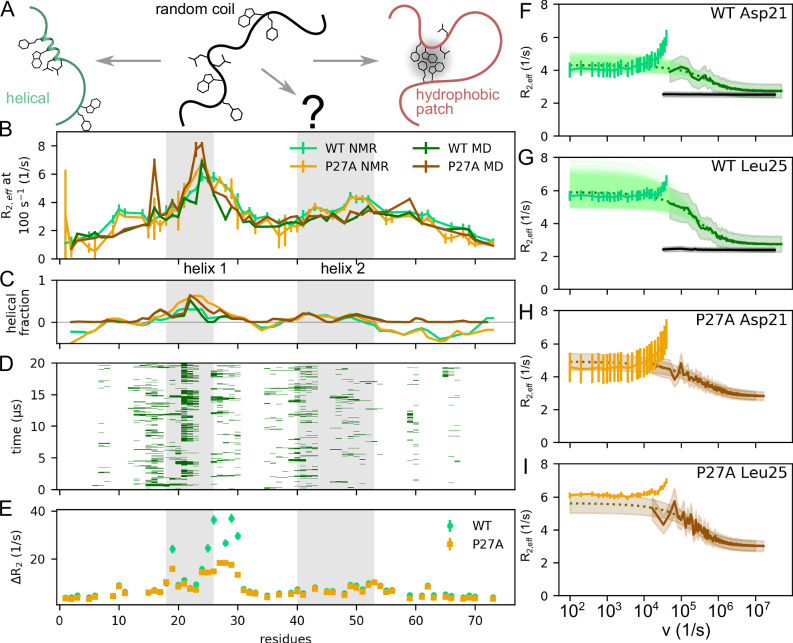
Tier 1 supra-*τ*_c_ dynamics of p53-TAD as observed by MD simulations at 298 K and compared to NMR RD measurements. **A** Schematic representation of two plausible structural origins of the observed supra-*τ*_c_ (tier 1) dynamics: folding/unfolding of transient helices and collapse/dissolution of hydrophobic clusters. **B** Comparison of amplitudes of 298 K NMR RD profiles at 100 s^−1^ CPMG frequency for each measured residue with those amplitudes extrapolated from MD simulations (darker colours) for WT (green) and P27A mutant (orange). Experimental and statistical uncertainties are shown as error bars (*n*_experiment_ = 2; *n*_MD_ = 30); grey shaded areas indicate helix 1 (18–26) and helix 2 (40–54). **C** Comparison of the helix fraction of each residue estimated from measured NMR chemical shifts and SSP with those derived from the MD simulations using dihedral angles (colours as in **B**). Negative values indicate a preference for extended conformations. **D** Dynamics of helix formation; green bars indicate instances during a representative 20 μs MD simulation at which each residue and its neighbours form a short helical segment. **E** Relaxation amplitude difference Δ*R*_2_ between the highest available CPMG frequency and that of relaxation due to overall molecular tumbling, as measured via TRACT experiments. Differences Δ*R*_2_ are shown for the WT (green dots) and the P27A mutant (orange squares). All measurements were performed at 263 K at a magnetic field strength of 1.2 GHz. Both NMR experiments used 2 repeated measurements. **F**–**I** Four examples of RD profiles (lighter solid lines) measured at 298 K and those calculated from the MD simulations (darker solid) and extrapolated to the NMR frequency range (dotted). As a control, black lines show RD profiles calculated from WT simulations for which unfolding of the α-helix was suppressed. Uncertainties of the measured profiles (vertical bars) were estimated from repeated measurements; for the calculated RD profiles, standard deviations around the mean are indicated as shaded areas.

Which structural motions cause the unexpectedly slow tier 1 dynamics of this IDP? As sketched in Fig. 2A, these dynamics might arise either from the folding and unfolding of transient secondary structure elements (e.g. the helices involving residues 18–26 or 40–55)^37^ or, alternatively, from the collapse and subsequent dissolving of a hydrophobic patch^50^—or due to other unknown structural dynamics.

Evidence for helix-formation dynamics comes from partial helicity seen in our NMR chemical shift measurements, which agree with previously observed^12^ helical populations between 15% and 30% for the residues within helix 1 (Fig. 2C). To test which of the above two processes causes the observed tier 1 dynamics, we also performed additional NMR measurements on the previously described Pro27→Ala mutant (P27A)^43^. Pro27 is a highly conserved residue and its alanine substitution has not been observed in naturally occurring variants. Rather, this mutant was originally designed to remove a “helix breaker” proline residue at the C-terminal end of helix 1, thereby changing its helical propensity^43^. If the tier 1 dynamics were dominated by helix folding and unfolding, one would therefore expect these dynamics to be altered in the P27A mutant. Indeed, the helical population of P27A is increased from 30% to 60% and, probably as a consequence^40^, this mutant also shows a markedly increased affinity to MDM2^51^.

However, our ^1^H E-CPMG RD profiles (Fig. 1C–E) measured at 263 K and at 1.2 GHz reveal that the timescale of the tier 1 supra-*τ*_c_ dynamics is the same for WT and P27A mutant. Unexpectedly, additional much slower 215 µs kinetics is detected for the P27A mutant, which is absent in WT p53-TAD (orange curves and symbols in Fig. 1C–E, G; see also Supplementary Information Fig. 3), pointing to distinct additional structural dynamics, different from—and much slower than—tier 1. We will refer to these new dynamics as “tier 0”, the structural origin of which is elusive at this stage.

Notably, because at each particular CPMG frequency the measured *R*_2,eff_ value contains relaxation contributions from all faster relaxation processes, the RD profiles shown in Fig. 1C–E (and in Supplementary Information Figs. 1, 4 and 5) also contain information about these faster conformational dynamics, even though these occur far outside the RD detectable timescale window between the rotational correlation time and ~4 μs. To disentangle the contributions from these faster kinetics from the components of the RD profiles that arise from exchange processes at the slower tier 0 relaxation frequencies, we independently determined residue-specific rotational correlation times *τ*_c_ via TRACT^52^ (TROSY for rotational correlation times) measurements (crosses in Fig. 1G). Accordingly, the difference Δ*R*_2_ between the *R*_2,eff_ value measured at the highest CPMG frequency and the TRACT-derived intrinsic transverse relaxation rate *R*_2,0_ quantifies the contribution from additional supra-*τ*_c_ dynamics that are faster than what could be detected at the maximum E-CPMG frequency (Fig. 2E). This finding provides an independent approach to detect dynamics faster than the timescale accessible to the E-CPMG RD measurements.

To isolate these fast contributions quantitatively, we subtracted the TRACT-derived relaxation rates from the RD measurements acquired at 1.2 GHz (Fig. 2E). Indeed, residues within and adjacent to helix 1 (residues 18–30) show markedly enhanced Δ*R*_2_, indicating the presence of dynamics that are slower than overall tumbling (*τ*_c_) yet faster than those directly captured by the E-CPMG RD experiments. Although the precise timescale of these structural dynamics cannot be determined from these NMR experiments alone, they clearly establish the existence of supra-*τ*_c_ tier 1 dynamics within this otherwise “blind spot” of NMR RD measurements.

### Unbiased atomistic simulations validated against NMR

To reveal the structural motions that cause the observed dynamics on all three tiers identified by NMR, we have carried out extensive MD simulations of both p53-TAD WT and P27A mutant in explicit solvent at 298 K. Contrary to previous studies^53,54^, these simulations are unbiased and have not been fitted to any p53 measurement, which allowed assessing their accuracy by calculating RD profiles, i.e., effective relaxation rates *R*_2,eff_, from the MD simulations. For each residue, two main components contribute to *R*_2,eff_, (1) relaxation arising from exchange between states (e.g., folding and unfolding) with different chemical shifts *R*_2,ex_; (2) relaxation caused by molecular tumbling and sub-*τ*_c_ internal motion summarised in *R*_2,0_. The effective relaxation *R*_2,eff_ contains both components *R*_2,ex_ and *R*_2,0_, which therefore were calculated separately from the trajectories. We note that due to the rich internal dynamics of this IDP, and in contrast to folded proteins, no well-defined overall tumbling timescale exists, which is why we resorted to a residue-wise analysis. Owing to the total MD trajectory length of 2.4 ms, the calculated RD profiles cover slow timescales up to 10 μs (sketched in Fig. 1B, black bar), thus enabling direct comparison with the experimentally determined ones.

To enable a direct comparison between RD profiles computed from our MD simulations and those measured by our NMR experiments, we measured full RD profiles also at 298 K. Figure 2F–I shows representative comparisons between the measured RD profiles (solid, lighter colours) and those calculated from the MD simulations (solid, darker colours) for two selected residues of both the WT (Fig. 2F, G) and the P27A mutant (Fig. 2H, I). Complete comparisons for all analysed residues are provided in Supplementary Information Figs. 4 and 5. Note that at higher CPMG frequencies, the measured RD profiles exhibit an unphysical increase, attributable to high-power artifacts likely arising from hardware limitations rather than sample heating. Therefore, the comparison between experiment and simulation was restricted to the low CPMG-frequency region of the RD profiles, which are free of such artifacts and reliably report on exchange dynamics.

To enable the most direct comparison, we fitted the RD profiles calculated from the MD simulations using Bayesian inference and a stretched CPMG model^55^ (dotted lines), without using the measured RD profiles. As described in more detail in Supplementary Information Fig. 6, this stretched CPMG model generalises the established two-state model^56^ to many states and, hence, represents a superposition of many relaxation rates. Very good agreement is seen, e.g. for residue Asp21 (Fig. 2F, H), whereas Leu25 (Fig. 2I) illustrates a typical deviation. Figure 2B compares the low frequency *R*_2,eff_ amplitude measured for all residues by NMR with those obtained from the MD simulations via the stretched CPMG fits.

Overall, good agreement is achieved for most residues, with an average deviation of ~15%. In particular, the dynamic profiles in Fig. 2B, showing higher amplitudes for the helical regions, are very similar in shape for both measured and calculated amplitudes. Deviations are seen mainly for the N-terminal region up to residue Val10, likely caused by the presence of 2–4 additional residues in the experiments, which were required for cloning. These were omitted in the MD simulations, which aimed at an accurate modelling of the WT p53-TAD. Indeed, after inclusion of these additional residues within the MD simulations, much better agreement is seen also for the N-terminal region, as shown in Supplementary Information Fig. 7. The deviation seen for Asn28 is presumably due to the absence of proline isomers in the MD simulations, supported by the fact that much better agreement is seen for the P27A mutant (Fig. 2B, orange). Further, the helical fractions for all residues calculated from the MD simulations (Fig. 2C, darker colours) also agree well with those derived from the NMR chemical shifts using SSP^57^ (lighter colours), see also Supplementary Information Fig. 8.

We note that the comparison (Fig. 2B, F–I) between the measured RD profiles and those calculated from MD simulations involves several sources of uncertainty. First, IDPs are known to be particularly sensitive to force field inaccuracies^58–60^; we have therefore carried out test simulations using different force fields and obtained similar profiles for the NMR observables (Supplementary Information Fig. 9). The best agreement was seen for the Amber99sbws force field, which we therefore chose for further analysis.

Second, despite milliseconds of sampling, the MD ensemble may not fully cover the full structural ensemble probed in the experiments. To assess convergence of our MD simulations, we compared observables calculated separately from three consecutive 20 μs blocks of the 60 μs long P27A trajectories. Chemical shift-based RD profiles (without the *R*_2,0_ tumbling related offset) were calculated for the full trajectories as described above and were averaged over 30 independent trajectories using the first, second and third 20 μs block of each trajectory (Supplementary Information Fig. 10). As can be seen, most averaged RD profiles are within their respective error ranges except those of residues Asp49, Phe54, Glu56, Glu62, Ala63, and Met66. The RD profiles of these residues, therefore, might not be sufficiently converged, and therefore their relaxation times were omitted from Fig. 2B.

Lastly, the accuracy of calculated RD profiles relies on that of the chemical shift calculations using SPARTA+^61^. To assess the accuracy of these empirical predictions, we have also calculated chemical shifts using SHIFTX2^62^ and obtained almost identical values, deviating by ca. 2.5% on average. Next, we compared calculated chemical shifts, averaged over our MD trajectories, to our own NMR measurements as well as to chemical shifts by Wong et al.^63^ (Supplementary Information Fig. 8). For the C_α_ chemical shifts, very good agreement is seen, with Pearson correlations of 0.996 and a mean absolute error of 0.26 ppm and 0.24 ppm (Supplementary Information Fig. 8A, C), respectively. Proton chemical shifts, from which RD profiles were calculated, show a Pearson correlation of over 0.76 and a mean absolute error of 0.13 ppm (Supplementary Information Fig. 8B, D). We note that the amplitudes of the RD spectra are sensitive to the somewhat larger uncertainty of the predicted proton chemical shifts and, therefore, the comparison of calculated to measured RD profiles provides an independent accuracy assessment. In contrast, relaxation times derived from calculated RD spectra are insensitive to such inaccuracies.

Notably, because the orientational correlation times were directly calculated from the orientational autocorrelation of the backbone NH bond vector without recourse to chemical shifts, their comparison with the measured TRACT relaxation times also provides an independent test of the accuracy of the simulations. Indeed, for all residues except the N-terminal ones, very good agreement is seen (Supplementary Information Fig. 11, *R*_2_ panel). Here, too, this deviation is mainly due to the additional residues required for sample preparation, as evidenced by the markedly improved agreement obtained for additional control simulations which included these residues (Supplementary Information Fig. 7). Finally, deviations between experiment and MD simulations might arise from the very slow isomerisation dynamics of prolines^64^, which are not described by our simulations. However, additional control simulations comparing, e.g., WT cis-Pro8 with trans-Pro8 showed no significant differences (Supplementary Information Fig. 11).

To further assess the accuracy of our unbiased MD simulations, we have calculated from our trajectories all observables derived from further independent calculations and experiments collected from the literature. These were ensemble averaged size determined by size-exclusion chromatography, small-angle X-ray scattering, and dynamic light scattering (Supplementary Information Fig. 12), as well as distances determined by fluorescence resonance energy transfer (Supplementary Information Fig. 13), photoinduced electron transfer fluorescence correlation spectroscopy (Supplementary Information Fig. 14), and paramagnetic relaxation enhancement (Supplementary Information Fig. 15). As discussed in more detail in these Supplementary Information Sections, good agreement is seen for all these observables.

### MD simulations reveal tier 1 helix-formation dynamics with multiple relaxation rates

Taken together, these quantitative comparisons of our atomistic simulations to a broad range of measurements suggest that the simulations describe the structural dynamics of p53-TAD sufficiently accurately to allow identification of the structural motions that give rise to the observed tier 1 dynamics. To this end, Fig. 2D shows the secondary structure dynamics particularly within the helical regions (grey), revealing sub-μs folding and unfolding of short helical structure elements (green bars). These dynamics are indeed the dominant cause of the observed NMR RD dynamics, as evidenced by control simulations, for which the α-helical regions have been forced to remain in helical geometry. Indeed, the RD profiles calculated from these control simulations (black curves in Fig. 2F, G) do not show any tier 1 dynamics. These results establish sub-μs helix folding dynamics as the structural determinant of tier 1, on top of the known and much faster tier 2 structural reorganisation dynamics^12,20^ (cf. Fig. 1B).

As can also be seen in Fig. 2D, these secondary structure folding dynamics are quite complex and are neither described by a fully cooperative two-state model, nor are the partial folding steps fully uncorrelated. Rather, these tier 1 dynamics seem to involve transitions between many different partially helical conformations and, accordingly, also involve many different timescales.

**Fig. 3:**
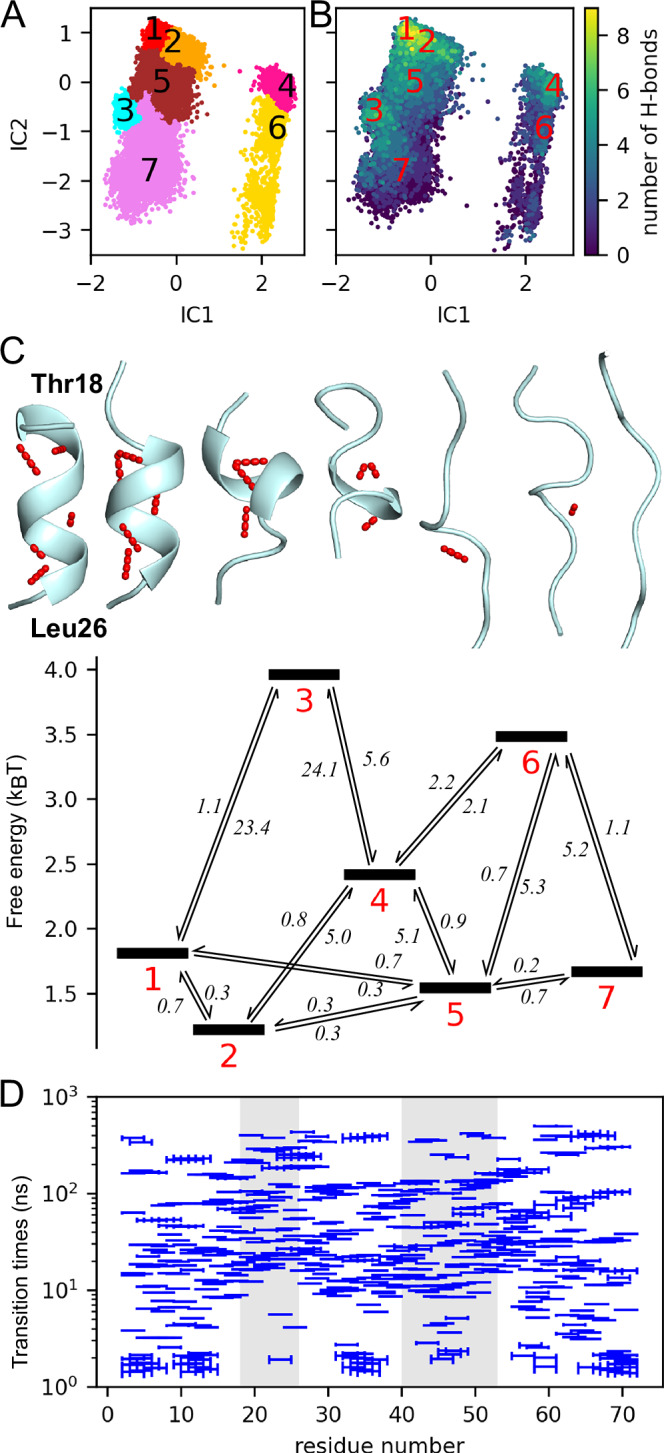
Markov state model reveals multi-timescale folding/unfolding dynamics of helix 1 underlying tier 1 motions of p53-TAD. **A** Projection of the conformational ensemble of helix 1 (residues 18–26) onto the TICA space defined by the two collective coordinates IC 1 and IC 2 that contribute most to the intra-helical dynamics using WT trajectories. Colours and numbers indicate seven Markov states. **B** The same projection as in (**A**), but coloured by the number of intra-helical hydrogen bonds. **C** Representative structures of the seven Markov states (intra-helical hydrogen bonds shown as red dots, top) and their free energies (black bars, below); mean first passage times of transitions between the states (arrows) are shown in μs. For a similar analysis (**A**–**C**) of helix 2, see Supplementary Information Fig. 16. **D** Per-residue timescale spectra (blue bars) calculated from MD trajectories, obtained using four residue (one helix turn) segments across the p53-TAD sequence (statistical uncertainties ( ± SD) shown as error bars). Grey shaded areas indicate helix 1 and helix 2 residues.

To characterise these partially cooperative tier 1 dynamics in more detail, we have used the WT MD trajectories to derive a Markov state model of the helix 1 conformational dynamics (Fig. 3). Figure 3A, B shows the MD ensemble after dimension reduction via time-lagged independent component analysis (TICA)^65^; each dot represents a structure snapshot, characterised by the most relevant independent components IC1 and IC2, which describe collective motions. In Fig. 3A, each of the obtained seven Markov states (colours and black numbers) represents a particular conformation of the helix, characterised in Fig. 3B by the number of intra-helical hydrogen-bonds (colours) ranging from ten (fully folded) to zero (unfolded). The conformations corresponding to these seven Markov states (top of Fig. 3C, hydrogen-bonds are shown as red dots) also display this sequence of decreasing helical structure. Our Markov state analysis yields free energy estimates for each state (bottom of Fig. 3C, thick bars) and transition rates between these states, ranging from 0.2 to 24.1 µs (arrows). These form a ‘folding funnel’ very similar to the one characteristic of folded proteins^7^. Resolved by residue, the whole spectrum of timescales of the p53 dynamics (Fig. 3D, blue bars) densely covers a range between a few and several hundred ns. Notably, here the Markov state model is not used to extrapolate to larger implied timescale otherwise inaccessible to the simulations^30^; rather, these dynamics are fully sampled by our simulations, and the Markov state model only serves to extract their rates and structural intermediates from the simulations.

Taken together, the IDP undergoes structural dynamics with transient and stepwise secondary structure formation that are far more complex than a simple two-state model. This finding also explains why a single two-state RD profile^56^ is unable to properly describe the E-CPMG RD profiles calculated from our MD simulations; instead, a stretched CPMG model that represents a superposition of many CPMG curves at different frequencies^55^ (indicated by the blue dashed lines in Fig. 1B), fits the calculated profiles very well (Fig. 2 and Supplementary Information Figs. 4 and 5). As illustrated in Supplementary Information Fig. 6 and described in more detail in the respective Supplementary Information Section, the stretching factor of approximately *γ* = 0.6 obtained from these fits is consistent with a timescale spectrum covering a similar range as the transition times shown in Fig. 3C. In a similar spirit as the stretched exponential rebinding kinetics originally proposed by Frauenfelder^5^ and more recently adapted by Palmer^24^, Edholm and Blomberg^66^, and Blackledge^67^, the broader spectrum of many relaxation rates described by the stretched CPMG model, can be understood as arising from a multi-state Markov model of interconverting conformational states which are separated by energy barriers of different heights. Specifically, the relaxation rates are the eigenvalues of the master equation that defines the Markov model^68^.

### Tier 0: multi-µs dynamics due to transient formation of tertiary structure elements resembling folding intermediates of natively folded proteins

**Fig. 4:**
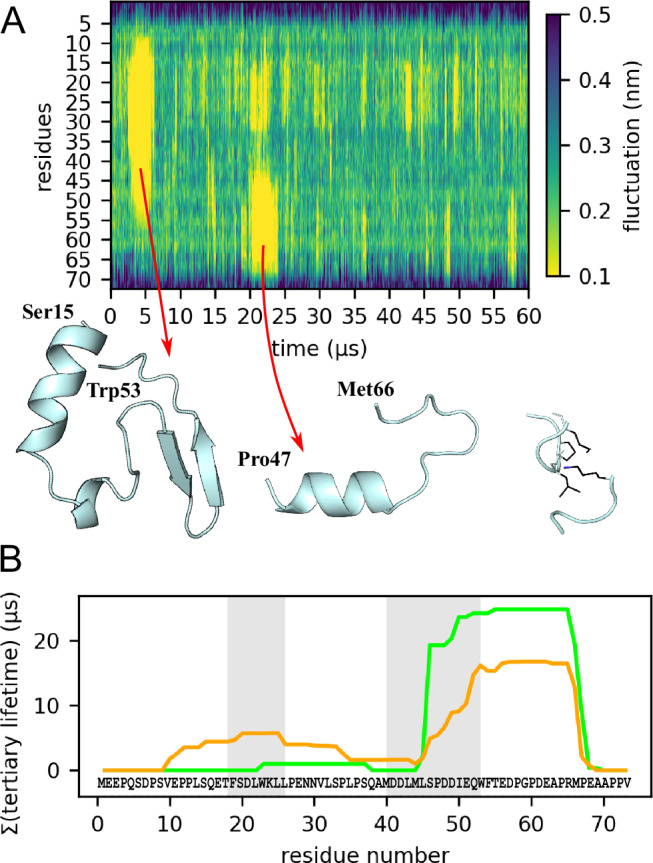
Residue distance fluctuation analysis reveals stable tertiary structures mainly around transient helices. **A** Example of C_α_-C_α_ distance fluctuations analysis. The trajectory visited multiple transient tertiary structures from which two are shown. For every residue, distance fluctuations, ranging from 0.1 (yellow, rigid) to 0.5 nm (dark blue, flexible), were determined and averaged over a 0.1 μs sliding time window. **B** Accumulated tertiary structural element lifetimes of all WT (green) and mutant (orange) MD trajectories, normalised by trajectory length to facilitate comparison. For the WT, particularly for the helix 2 region, these lifetimes are dominated by very few long-lived tertiary structures and, therefore, are subject to large statistical uncertainty. Grey shaded areas indicate helix 1 and helix 2 residues.

Which structural motions give rise to the slow 215 µs (tier 0) kinetics revealed by the NMR RD profiles of the P27A mutant (indicated by the left side of the blue curve in Fig. 1B)? To answer this question, we systematically searched for long-lived stable structures in our MD trajectories. Indeed, a residue-resolved analysis of C_α_-C_α_ distance fluctuations (Fig. 4A) revealed sporadic multi-µs collective excursions of pronounced stability, which are both larger and longer lived than the transient helices (Fig. 2D) of tier 1. We identified a total of 18 such long-lived events in the 0.6 ms WT simulations and 75 for P27A, three of which are shown in Fig. 4A. They represent metastable tertiary structure elements larger than simple α-helices, which persist up to 5 μs and are mostly stabilised by hydrogen bonds. We note that the observed µs persistence times cannot be quantitatively translated into kinetic rates, because rare and longer-lived tertiary structures may have been missed by our simulations. Therefore, the actual formation rates may be slower.

For the P27A mutant, the simulations suggest that the removal of the sterically restrictive proline increases the conformational flexibility of p53-TAD, which enables the formation of larger and longer-lived tertiary structures. The larger number of long-lived structure formation events, along with their increased total lifetime seen in the P27A simulations, explains the much more pronounced low-frequency signal seen in the P27A CPMG RD profiles.

Some of these structures are also stabilised through hydrophobic contacts (e.g. the rightmost example in Fig. 4A); hence, and in contrast to the faster tier 1 dynamics, hydrophobic collapses seem to contribute to the tier 0 dynamics. Notably, these long-lived tertiary structures frequently form in the presence of (and involve) one of the two otherwise much shorter-lived α-helices (Fig. 4B and, e.g. the largest structure shown in Fig. 4A), thereby stabilising these helices over much longer timescales. This observation suggests that, at least for this particular IDP, the α-helices form “nucleation seeds”, which promote subsequent tertiary structure formation attempts, similar to primary folding intermediates seen for natively folded proteins^69,70^.

This effect is particularly notable for tertiary structures involving residues 45–65, where only the first few residues located in the N-terminal segment are involved in helix formation (Fig. 4B). In contrast, the terminal regions of the protein exhibit only fast reorganisation dynamics, whereas there are also segments outside the helix 1 and helix 2 regions (e.g. residues 10–35 particularly for P27A) for which formation of transient tertiary structures associated with slow dynamics are seen. This finding also indicates that secondary structure formation is not a strict prerequisite for transient tertiary formation and the associated slow tier-0 dynamics. This pronounced sequence and structure dependent heterogeneity adds to the complexity of the underlying hierarchical free energy landscape of this IDP.

### Tier 2: a stretched spectral density function reflects complex multistate dynamics also at fast nanosecond timescales

Contributing to the NMR RD profiles are also faster, ns and ps motions, which have been probed by regular relaxation experiments^24^ and here add an offset to *R*_2,eff_. These motions are characterised by the spectral density function (SDF), which is the Fourier transform of the correlation function of the dipolar couplings and the chemical shift anisotropies^71^. Despite its central role, the full SDF is inaccessible to experiments; rather, only isolated points of the SDF at specific frequencies can be determined, using combinations of multiple NMR measurements such as *η*_*xy*_, *τ*_c_, *R*_1_, *R*_2_, and NOE at different conditions^10^.

**Fig. 5:**
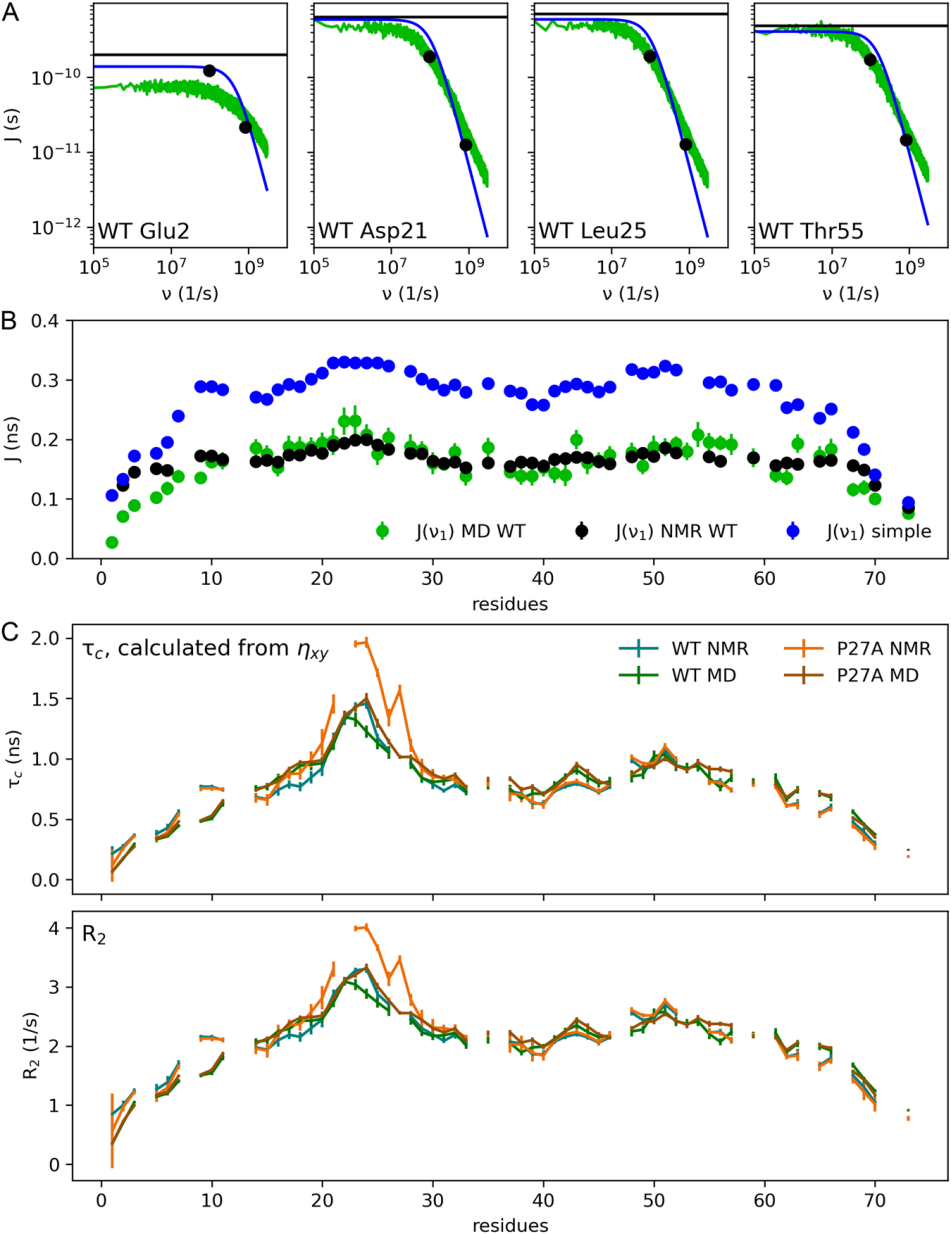
Comparison between calculated spectral density functions (SDFs) *J(ν)* with NMR measurements at 298 K. **A** Examples of SDFs calculated from our MD simulations (green lines) and measured SDF values at frequency *v* = 0 (horizontal black lines) as well as at frequencies *v*_1_ = ω_N_/2π = 96.3 MHz and *v*_2_ = εω_H_/2π = 826.5 MHz, respectively (black dots) for the residues of the p53-TAD shown in Fig. 2F–I and for two additional residues of the N-terminus and of helix 2. Blue lines show SDFs calculated from a simple two-state model. Per literature convention, frequencies *v* are given in units 1/s, whereas frequencies *ω* are in units rad/s. To facilitate easier comparison with low-frequency spectra from CPMG experiments, we here show high frequency spectra such as the SDFs also as a function of *v* and in units of 1/s. **B** Measured SDFs at *v*_1_ = *ω*_N_/2π = 96.3 MHz (*J*(*v*_1_), black dots, *n* = 2) compared to values calculated from our MD simulations (green, *n* = 30) and predicted from a simple two-state model (blue); Data are presented as mean values ± SEM and are smaller than the symbols. **C** Comparison of *τ*_c_ and the rate constant *R*_2_ derived from NMR measurements at 1.2 GHz (*n* = 2) with values calculated from MD simulations (*n* = 30) for the p53-TAD WT (turquoise and green lines) and the P27A mutant (orange and brown lines). For proline residues without backbone amide protons, no spectra were obtained. Complete results are provided in the Supplementary Information Figs. 11, 17 and 18. Data are presented as mean values ± SEM.

The considerable length of our MD trajectories enabled us to calculate full SDFs of p53-TAD. Figure 5A shows examples for four residues, two of which are also shown in Fig. 2; SDFs for all residues are shown in Supplementary Information Fig. 17. For each residue, the SDF was calculated over a broad frequency range covering more than four orders of magnitude (green lines). Notably, the SDF calculated from our MD simulations are logarithmically stretched relative to the Lorentzian functions that would result from a single *τ*_c_^24^, which we refer to as “simple model” (blue lines), as evident from the shallower slope at higher frequencies. In this simple model (Eq. (7)), the underlying dynamics are described by a single tumbling timescale *τ*_c_, which we measured by the TRACT experiment described above. This marked logarithmic stretching was observed for all residues and is in line with a similar observation for RD profiles (Supplementary Information Fig. 6), which strongly suggests that also the tier 2 reorientation dynamics (which determine the SDF) comprise not one but many timescales. A plausible explanation could be that tumbling motions of certain residues depend on their immediate environment and are, e.g., slower when in a transient helical conformation than in a disordered state.

For comparison to the experiment, we determined SDFs *J*(0), *J*(ω_N_), and *J*(εω_H_) at three distinct frequencies (black line and dots in Fig. 5A and Supplementary Information Fig. 17) using multiple NMR measurements at various conditions and using ε = 0.87 as described earlier by Farrow et al.^72^. As can be seen, these agree much better with the SDF values calculated from our MD simulations than with the simple model, as is also evident for all other residues (Supplementary Information Fig. 18). Notably, at *v*_1_ = 96.3 MHz (Fig. 5B), calculated and measured values agree very well, whereas the simple model (blue) consistently predicts larger values, due to the different slopes of the high-frequency parts, underscoring the multi-timescale reorientation dynamics of p53-TAD within tier 2. At the highest frequency (*v* = 826.5 MHz, Supplementary Information Fig. 18C), the simple model predictions agree better with the measured values, which, however, we consider largely coincidental, due to the fact that this frequency is often close to the intersection point of the two spectra (cf. Supplementary Information Fig. 17). While the SDF profile *J*(*v*_2_) calculated from our MD simulations has a quite similar shape as the measured one, the values are consistently too large. The reason for this discrepancy remains unclear; because the nanosecond dynamics produced by the Amber99sbws force field is known to be slightly too slow (cf. Table 2 in ref. ^73^), we speculate that equipartition might imply correspondingly larger amplitudes at these fast frequencies. In addition to this systematic deviation, larger differences are seen for the N-terminal residues up to Val10, again likely due to the few residues added during cloning, which were absent in the simulations.

A more direct comparison to NMR is achieved by calculating the above five NMR observables *η*_*xy*_, *τ*_c_, *R*_1_, *R*_2_, and NOE, from the MD simulations (Fig. 5C shows *τ*_c_ and *R*_2_; all five observables are shown in Supplementary Information Fig. 11). Overall, good agreement is seen, facilitating structural interpretation of the parts of the SDFs that are inaccessible to experiment. Deviations are seen for mutant residues particularly in the region 23–27, which is most likely due to the pronounced tier 0 dynamics of P27A. Whereas we used the longest possible autocorrelation window size (10 µs) for the calculation of the SDFs, the experimental timeframe is much wider and thus can detect (and be affected by) slower events. Notably, the SDF calculated from the simple model generally deviates more from the experiment at *v* = 96.3 MHz than the SDF calculated from our simulations, providing further and independent evidence for the presence of multi-state dynamics covering a wide spectrum of timescales also within this fast tier 2 regime. Indeed, such fast motions have been observed for different IDPs previously, such as librational motions of amide groups, fast backbone conformational fluctuations, and slow chain segmental motions^67,74^, which all can contribute to the measured relaxations.

Because the orientational dynamics within secondary structure elements are expected to be slower than those of disordered regions, residues that are involved in the formation of transient helices should show correspondingly slower correlation times. Indeed, whereas fast sub-ns dynamics are seen for the terminal regions, the local reorientation dynamics of residues with substantial helical population (grey shaded areas Fig. 2) slow down to 1 to 2 ns (Fig. 5C, panel *τ*_c_). These timescales agree well with previous NMR measurements^12^ as well as with single-molecule FRET experiments on the Csp*Tm* cold-shock protein^16^, underscoring the slower rigid-body motion of these transient helices within the faster rearranging disordered phase.

Our simulations also enable comparison of the fast (tier 2) conformational dynamics of p53 to polymer models^21,54,75^. In particular, p53 is highly and rather homogeneously flexible with a persistence length of 2–3 amino acids (Supplementary Information Fig. 19A, B). Its scaling exponent of *v* = 0.66 is compatible with a self-avoiding chain model^76^, which agrees with previous measurements of polymers in good solvents^77^ and is above the critical Θ-point, at which chain-chain and chain-solvent interaction balance at the thermodynamic phase boundary^78^.

Exponential fits to the orientation autocorrelation function (Supplementary Information Fig. 19D) show even higher flexibilities at short contour lengths, in line with single-molecule FRET experiments^16^. An unexpected additional component is seen with a considerably longer persistence length of over 5 nm, pointing to so far unresolved partial long-range order, likely due to self-crowding governed by the slower tier 1 and tier 0 structural dynamics. These findings are supported by similar fast components of the time-lagged structure RMSD decay as well as time autocorrelation functions of the radius of gyration and end-to-end distance (10 ns, 22 ns, and 18.5 ns, respectively, Supplementary Information Fig. 19E, F). The latter two also reveal additional, much slower dynamics of 405 ns and 350 ns, respectively, likely arising from tier 1 dynamics. Overall, the structural dynamics of p53 resemble those of a highly flexible heteropolymer with fast reorganisation dynamics^77^ at short contour lengths and additional slower dynamics at larger length scales that give rise to pronounced long-range correlations.

## Discussion

**Fig. 6:**
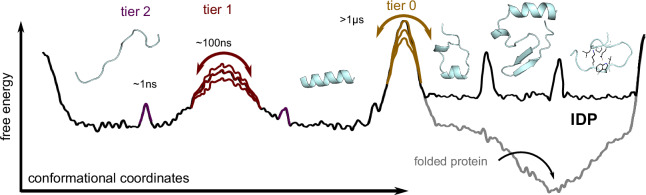
Sketch of the p53-TAD IDP hierarchical free energy landscape emerging from our combined NMR/MD study. Schematic free energy landscape (black curve) summarising the three distinct tiers of p53-TAD dynamics emerging from our combined NMR/MD study. A hierarchy of free energy barriers (coloured) similar to that known for folded proteins^5^ governs fast (ns) reorientation and reconfiguration dynamics (tier 2 in purple), slower (sub-µs) supra-*τ*_c_ dynamics arising from transient helix formation (tier 1 in red), and very slow (multi-µs to ms) formation of transient tertiary structure elements (tier 0 in beige). The indicated variation of barrier heights is also similar to that observed for folded proteins and gives rise to a correspondingly broadened spectrum of relaxation times, which explains the observed stretched RD profiles. Contrary to folded proteins (grey curve), the transient tertiary structure elements of IDPs (tier 0) are only metastable and difficult to access experimentally. Structures shown as ribbon plots within the respective energy wells indicate typical intermediates seen in the MD ensemble.

Our high-power RD NMR measurements (E-CPMG)^45^ performed at 1.2 GHz combined with milliseconds atomistic MD simulations revealed a hierarchy of complex structural dynamics of the intrinsically disordered transactivation domain of the central gene regulation protein p53 (p53-TAD), which is known to form transient α-helices^12^ that are relevant for binding and molecular recognition^23,35^. Covering a dynamic range of over seven orders of magnitude, from ps to sub-ms, we found three tiers of quite diverse structural dynamics (Fig. 6). This finding follows independently and consistently from our NMR measurements as well as from our MD simulations. Combined, our results markedly extend the timescale range at which IDP structural dynamics is observed at atomic scales, add structural and dynamic detail to transient structure formation in IDPs^20,22^, and thereby further challenge the earlier notion that the lack of stable folded structures of IDPs implies a shallow or weakly funnelled free energy landscape that gives rise only to rather simple and fast tier 2 reorganisation dynamics with occasional secondary structure^17,18,20^.

Instead, our findings suggest that the classic Frauenfelder stretched exponential description of folded protein dynamics, which arises from an underlying multi-tier hierarchical free energy landscape^5^, also applies to the largely unstructured IDP p53. Notably, we have obtained similar results for Measles Virus N_TAIL_ (Supplementary Information Fig. 20), an unrelated IDP. N_TAIL_ is a pre-molten globule type IDP^79^ with little secondary structure except for a short, transiently helical molecular recognition element (MoRE) that forms a stable four-helix bundle when binding the Measles phosphoprotein X-domain^80^. Recent PET experiments revealed N_TAIL_ dynamics that markedly deviate from simple homopolymer model predictions, which can be explained by the presence of transient tertiary structures that have been observed in recent molecular dynamics simulations^81^. Combined, our findings suggest that these unexpectedly complex hierarchical dynamics may be a feature of other IDPs.

In particular, transient secondary structure formation dynamics are seen on a broad range of supra-*τ*_c_ (10s to 100s of ns) timescales between several ns and sub-µs, forming a second dynamics level, tier 1. In our simulations, these serve as dynamic precursors for the tier 0 formation of metastable and quite diverse tertiary structures at different positions along the TAD sequence. We speculate that these may be a key factor in the ability of p53 to fold into a broad range of different structures upon binding to a similarly broad range of different partners. Indeed, a structure similarity search in the Protein Data Bank (PDB)^82^ revealed that motifs of these tertiary structures seen in our MD simulations also occur in proteins (Supplementary Information Fig. 21). Because such rich conformational selection dynamics has the potential to accelerate binding, this third dynamics tier, emerging from an unexpectedly complex free energy landscape (Fig. 6), may thus be not only a feature of p53-TAD, but also a key to understanding the binding promiscuity of many other IDPs.

Adding further complexity, this timescale hierarchy is expressed at varying degrees along the protein sequence. In particular, whereas the dynamics of the terminal segments are dominated by tier 2 dynamics, tier 1 dynamics are more restricted to the helical regions. Although these tier 1 dynamics serve as precursors for tier 0 dynamics in these regions, transient tertiary structure formation is also observed independent of helical structures, e.g., for residues 56–65 (Fig. 4B).

Our results show that the structural dynamics of IDPs are not limited to fast and essentially random reorganisations, but are organised across a broad hierarchy of timescales, as is characteristic for the conformational dynamics of natively folded proteins. This complexity refines the notion of an inverted free energy landscape^83^ and highlights similarities to the (un-)folding dynamics of natively folded proteins, where both MD simulations^84^ and Φ-value measurements^85^ have revealed transiently stable secondary and tertiary structure elements along unfolding pathways, similar to those seen here for p53-TAD in equilibrium. Knowledge of such transient structures may help shift their conformational ensembles and modulate their activity, such that these structures may become potential drug targets. Unbiased MD ensembles such as the one presented here, which are consistent with NMR and other measurements, may also provide valuable training data sets for deep learning generated IDP conformational ensembles and dynamics.

## Methods

### Sample preparation

Recombinant p53-TAD (1–73) was expressed as a fusion protein with an N-terminal Z_2_ domain using a modified pET28a vector^86^. Perdeuterated ^15^N-labelled p53-TAD (1–73) samples were expressed at 25 °C in *E. coli* adapted to 100% D_2_O minimal medium supplemented with D_7_-glucose as the carbon source and ^15^N-NH_4_Cl as the nitrogen source. Protein expression was induced with 1 mM IPTG. The expression culture was harvested 12 h after induction. Recombinant p53-TAD was purified using immobilised metal affinity chromatography on Ni-NTA resin (Macherey-Nagel, Germany) followed by Tobacco Etch Virus (TEV) protease cleavage at room temperature. The cleaved protein was reloaded onto Ni-NTA resin to remove the Z_2_ domain and TEV protease. Gel filtration on a Superdex 75 16/60 HiLoad column (GE Healthcare) was performed to further purify the p53-TAD (1–73) fragment. Due to cloning, the WT fragment included an additional N-terminal Gly-Ser-extension and the P27A mutant a Gly-Ser-His-Met-extension. The fractions containing the purified protein were combined, concentrated with a 10 kDa concentrator (Vivascience).

### Sample conditions

Nuclear Magnetic Resonance (NMR) experiments were performed on a 1.5 mM p53-TAD sample in 50 mM sodium acetate buffer at pH 6.3, containing 50 mM sodium chloride and 0.03% sodium azide. Backbone amide ^1^H Off-resonance relaxation dispersion (*R*_1ρ_-RD) experiments at temperatures ranging from 262 K to 265 K were carried out in glass capillary tubes to produce super-cooled conditions below the freezing point of water. Each capillary of 1 mm outer diameter (Wilmad, Buena, New Jersey) contained 25 µl of the p53-TAD sample, and 12 such capillaries were placed inside a 5 mm NMR sample tube. The sample was purposefully not labelled with ^13^C nuclei to avoid the necessity of ^13^C decoupling, which could be an extra source for RF heating. In addition, the heteronuclear J coupling of C_α_ and carbonyl carbon to the nearby amide proton and nitrogen nuclei can be a source of artifacts in relaxation dispersion profiles.

All NMR sub-*τ*_c_ relaxation measurements and ^1^H_N_ extreme power Carr-Purcell-Meiboom-Gill (CPMG) RD measurements were performed on uniformly perdeuterated, ^15^N labelled proteins (both p53-TAD WT and P27A mutant) back exchanged with 100% H_2_O. The samples were finally buffer exchanged to 50 mM sodium acetate buffer at pH 6.3 containing 50 mM NaCl, 5% (vol/vol) D_2_O, and 0.02% sodium azide. The final p53-TAD protein concentrations of WT and P27A samples were 1.0 mM and 0.7 mM, respectively. Each sample was transferred to a 2 mm capillary and was placed within the magnet using a Bruker Match insert assembly.

### NMR relaxation measurements

All NMR spectra were collected with a Bruker Avance III HD spectrometer and corresponding Topspin 3.7 software operating at 950 MHz equipped with a TCI 5 mm cryo-probe and a Bruker Neo spectrometer (Topspin 4.0) operating at 1.2 GHz ^1^H field strengths, equipped with a TCI 3 mm cryo-probe. Sample temperature was controlled with dry N_2_ gas using Bruker BCU-II VT units with medium chiller strength and 670 litre/hour gas flow for all experiments. Temperatures over the 263–298 K range were calibrated using a 3 mm Greisinger GMH 3750 thermometer equipped with a thermocouple. All spectra were referenced with respect to the water peak.

2D−^15^N−^1^H spectra, collected with a FAST-HSQC^87^ pulse sequence at each temperature, were used to validate sample conditions. Assignments were transferred from previously published sources^88^ at 298 K and propagated to spectra collected at 263 K by recording a series of 2D-HSQC spectra at 5 K temperature intervals. All 2D-NMR data were processed within the UNIX software environment NMRPipe^89^ (version 12.6) and were further analysed and visualised using the software package nmrfam-sparky^90^ (version 3.190).

The ^15^N *R*_1_, *R*_1ρ_ experiments were used for mapping points of the spectral density function^10^ and recorded at 298 K, and 263 K under 950 MHz ^1^H field strength using standard protocols^91^ and an eight-point measurement scheme (with two repeat points). ^15^N-*R*_1ρ_ relaxation rates were measured using a spin lock field of 2 kHz^92^. Heteronuclear ^15^N nuclear Overhauser effect (NOE) measurements were recorded with 5 s mixing time using standard protocols^91^ at 298 K under 950 MHz ^1^H field strength.

The amide backbone ^1^H Off-resonance *R*_1ρ_ RD experiments were recorded for the WT p53-TAD at supercooled temperatures 262 K, 263 K, 264 K, and 265 K at a 950 MHz ^1^H field^93,94^. This narrow temperature range was limited by two constraints, (1) the requirement that the exchange process remains within the sensitivity window of NMR RD, and (2) sample freezing at 261 K even under the supercooled conditions inside capillaries. The pulse sequence of ref. ^94^ was taken. The spin-lock period t_rel_ is flanked by adiabatic half-passage pulses (amplitude tailored according to the tanh function, frequency sweep controlled by the tan function, sweep width 100 kHz, duration 4 ms, the two half-passage pulses are related via time reversal)^95^. These pulses are integrated in the spin-lock period such that their maximum amplitude is equal to the spin-lock amplitude (up to 35 kHz). The proton RF field is applied at water line, except during the spinlock where the carrier is positioned at 8 ppm±Δ, where Δ = √2 × spinlock amplitude. Nitrogen decoupling during the acquisition is achieved using a WURST sequence^96^ with maximum RF field strength of 1.2 kHz. The phase cycle is: *ϕ*_1_ x,-x; *ϕ*_2_ 2x, 2y, 2(-x), 2(-y); *ϕ*_3_ and *ϕ*_4_ x; *ϕ*_rec_ x,-x, -x, x. Quadrature detection in F1 is achieved using the sensitivity enhancement scheme^97,98^ by recording two datasets with (*ϕ*_4_, g5) and (*ϕ*_4_ + 180°, -g5) for each t_1_ increment. Phase *ϕ*_1_ is incremented in concert with receiver phase to shift axial peaks to the edge of the spectrum^99^. Gradient pulses g1 to g7 are applied with durations 1.0, 0.5, 1.0, 1.0, 1.25, 0.5, and 0.125 ms and amplitudes 5.0, 4.0, 20.0, 12.0, 15.0, 4.0, and 14.86 G/cm, respectively. The delays are *τ*_a_ 2.25 ms, *δ*_1_ = 1.5 ms, *δ*_2_ = 0.5 ms. The spin-lock power was varied between 1 kHz and 35 kHz during the measurement to obtain effective transverse relaxation rates at different RF field. A 298 K timescale between 66 and 100 ns ( ± 1*σ* uncertainty) was estimated by Arrhenius extrapolation from these four temperatures, validated against an Eyring estimate, and the uncertainty of this estimate was determined using Bayesian inference.

Site-specific ^15^N-transverse cross-correlated relaxation (CCR) rates (*η*_*xy*_) were measured via 2D ^15^N TRACT (TROSY for rotational correlation times)^100,101^ experiments performed at 298 K, and 263 K under 950 MHz and 1.2 GHz field strengths. The software NMRPipe was used to process all pseudo-3D spectra as well as to extract relaxation rates. Uncertainties in measured relaxation rates were estimated using error propagation from spectral RMS noise. Measured *η*_*xy*_ values were converted^52,102^ to approximate site-specific rotational correlation times (*τ*_c_) and further converted to approximate chemical exchange free intrinsic ^15^N and ^1^H transverse auto-relaxation rates (*R*_2,0_) as published^103^ using Python (version 3.8) scripts. For all calculations, standard values^52^ for ^15^N−^1^H bond length 1.02 Å, *θ*_*xy*_ = 17°, ^15^N CSA = -160 ppm, and ^1^H_N_ CSA = 10 ppm^104^ were used. The effect of varying these parameters in the calculation of *τ*_c_ has been described in detail elsewhere^52^. The relaxation data was visualised using OriginPRO software (version 10.1).

One set of ^1^H_N_ Carr–Purcell–Meiboom–Gill (CPMG) experiments using extreme power were recorded at 263 K and 298 K with the published pseudo-4D IP/AP scheme pulse sequence^105^ with modifications as published in ref. ^45^, required to run the experiment at extreme CPMG frequencies^106^. The experiments^106–109^ were performed with the power on the ^1^H channel set to 18 W (^1^H 90° pulse length ~ 8.1 μs) on the 950 MHz spectrometer and ~ 16.3 W (^1^H 90° pulse length ~ 6.25 μs) on the 1.2 GHz spectrometer. A recycle delay of 3 s for all experiments was used to ensure minimal sample heating and a low-duty cycle. All experiments were recorded with 128 initial dummy scans to equilibrate the spin system and sample temperature before data acquisition. For the 1.2 GHz experiments, the constant CPMG duration (T_CP_) was set to 20 or 40 ms, and 28 points were sampled in the CPMG frequency dimension (including the reference plane and two repeat points) ranging from 100 Hz up to 40 kHz. For each experiment, 100–120 (indirect dimension) and 1536 (direct dimension) complex points were recorded with 16 scans using 28 and 16 ppm spectral width along the indirect and direct dimension, respectively. The experiments were recorded with a 2 ppm bandwidth E-BURP refocusing pulse (centred at the middle of the ^1^H_N_ region ~ 8.0 ppm) at the centre of the CPMG duration. The total experiment time was ~ 90 h at each temperature for each sample. The experiments at 950 MHz were acquired with 30 points (including the reference plane and two repeat points) in the CPMG frequencies dimension, ranging from 100 Hz up to 30.7 kHz, and with a hard pulse at the centre of the CPMG duration. For each experiment, 120 (indirect dimension) and 1024 (direct dimension) complex points were recorded with 4 scans, totalling ~ 24 h experimental time per sample at each temperature.

For all experiments, spectra were processed, and relaxation rates were extracted separately for the IP and AP sets of spectra using the UNIX software environment NMRPipe, followed by averaging for subsequent analysis. The differences between values from the IP and AP datasets were minimal. Site-specific solvent exchange contributions to measured ^1^H_N_ *R*_2_ values at 298 K were estimated at 950 MHz, using differences of site-specific ^1^H *R*_1_ values acquired from two sets of inversion recovery pulse sequences recorded with standard parameters and recycle delays of >10 s. In the first experiment, water magnetisation was kept along the *z*-axis, whereas in the second experiment, it was completely dephased with a low gradient^110^.

All fourteen proline NH resonances (Fig. 1A) were absent in all NH HSQC planes. In the relaxation measurements for the WT p53 sample at 263 K, the resonances of residues D7 and D57 overlapped, and at 298 K the resonances of residues W53 and F54 overlapped, both due to limited resolution in these experiments. Similarly, overlaps occurred for the P27A p53 mutant sample at 263 K for resonances of residues D7 and D57, and at 298 K for resonances of residues D7, L22, W53, W54, and D57. For these, relaxation rates could not be determined.

The 2D [^1^H-^1^H]-NOESY experiment was collected on a Bruker Avance III HD spectrometer operating at 900 MHz equipped with a TCI 5 mm cryo-probe at 298 K. The NOESY experiment was performed with 120 ms mixing time and 1024 and 512 complex points along *t*_1_ and *t*_2_ dimensions, respectively. The NOESY data were processed using NMRPipe^89^ and analysed with nmrDraw (version 12.6) and CARA^111^ (version 1.9.9).

### Molecular dynamics simulations

All simulations were performed using the molecular dynamics (MD) simulation software package GROMACS Version 2019.3^112^. Starting structures for each of the 30 trajectories were generated by first collapsing a fully extended p53-TAD (1–73) molecule performing a short generalised Born implicit solvent (GBSA) simulation^113^. From these trajectories structures were selected which were not fully collapsed (radius of gyration >3.5 nm) and did not contain any secondary structure elements. The p53-TAD (residues 1–73) WT protein and the P27A mutant were placed within dodecahedral boxes with an initial volume of 1222 nm^3^ (edge lengths: 12.0, 12.0, 8.485 nm) solvated in water and 100 mM NaCl. Virtual sites were used to allow for a time step of 4 fs^114^. The LINCS algorithm^115^, applying a sixth-order iterative restraint on the bond distances, was used. The Particle Mesh Ewald (PME) algorithm^116^ was used for electrostatic interactions with a cut-off of 1.0 nm. A reciprocal grid of 96 × 96 × 96 cells was used with fourth order B-spline interpolation. A single cut-off of 1.215 nm was used for the Van der Waals interactions. Neighbour searching was performed every 60 steps. Temperature was controlled by the velocity-rescale algorithm^117^ with a 298 K target temperature and a 0.1 ps coupling constant; for pressure coupling the Parrinello-Rahman algorithm^118^ was used with 1 bar target pressure and a coupling constant of 20.0 ps. All fully hydrated systems were allowed to equilibrate for 10 ns before the final production runs were started. Protein coordinates were recorded every 100 ps. All WT simulations were 20 μs long and all P27A simulations were 60 μs long to achieve better convergence for the expected slower P27A timescales. The Amber99sbws^119^ force field was used in combination with the TIP4P2005s^120^ water model.

### Exchange related relaxation: *R*_2,ex_

Conformational dynamics such as folding and unfolding of an intrinsically disordered protein (IDP) induce changes in the chemical shift of the involved nuclei and were probed by CPMG RD experiments as described above. By fitting to the CPMG curve (Eq. (18)), the characteristic exchange timescale between the folded and unfolded states was determined for those residues for which the exchange rate falls within the detectable frequency range set by the maximum applicable RF power and where the chemical shift difference is sufficiently large. RD profiles were calculated from our MD simulations via the power spectrum of the combined chemical shift time traces CS_HN_(*t*), calculated for all available trajectories of the respective nuclei. Chemical shifts of the p53-TAD backbone protons were predicted using the software SPARTA+^61^ from simulation frames separated 100 ps in time from 20 μs trajectories for the WT and 60 μs trajectories for the P27A system. As shown by Xue et al. earlier, the RD profile is derived from the chemical shift autocorrelation functions^121,122^, which we have calculated from CS_HN_(*t*) via the properly normalised power spectrum, i.e. the absolute-squared Fourier transform

1 $${R}_{2,{{{\rm{ex}}}}}\left(\nu \right)=\frac{1}{2}N\Delta t {\left|{{{\mathscr{F}}}}\left({{{\rm{C}}}}{{{{\rm{S}}}}}_{{{{\rm{NH}}}}}\left(t\right)\right)\right|}^{2}$$

using the Wiener-Khinchin theorem^123^. Here, *v* is the CPMG frequency, *N* is the number of simulation frames, Δ*t* is the time step between frames (100 ps). Note that the unit of CS_HN_(*t*) is rad/s. RD profiles were calculated separately from each MD trajectory and then averaged.

### Tumbling related relaxation: *R*_2,0_

To estimate *R*_2,0_, the spectral density function (SDF) was calculated for each residue as described subsequently. From the SDF of each residue, *R*_2,0_ was calculated via Eq. (13) and used as the offset for the calculated RD profiles.

### Calculation of the spectral density function and derived NMR observables *η*_*xy*_, *τ*_c_, *R*_1_, *R*_2_, and NOEs

SDFs *J*(*v*) were calculated for each residue from the Fourier transformation

2 $$J\left(\nu \right)=\frac{1}{5}{t}_{\max }{{\mathrm{Re}}}\left({{{\mathscr{F}}}}\left(C\left(\tau \right)\right)\right),$$

of the backbone H-N-bond rotation-autocorrelation function *C*(*τ*)^71,124^

3 $$C(\tau )=\left\langle {P}_{2}({{{\boldsymbol{\mu }}}}(t) \cdot {{{\boldsymbol{\mu }}}}(t+\tau ))\right\rangle,$$

where *τ* is the lag time, ***μ***(*t*) is the unit vector of the covalent bond, *P*_2_ is the second Legendre polynomial, *t*_max_ = 100 ns is the longest lag time considered, and 〈 〉 denotes the time average along the trajectories. Due to the symmetry of the autocorrelation function, the real part of the Fourier transform is used for this calculation.

By combining the values of the SDF at specific frequencies, the transverse cross-correlation rate constant *η*_*xy*_ and tumbling timescale *τ*_c_ were calculated using Eqs. (4)–(8), respectively, as described by Robson et al.^52^,

4 $${\eta }_{{xy}}=p{\delta }_{{{{\rm{N}}}}}(4J(0)+3J({\omega }_{{{{\rm{N}}}}}))(3{\cos }^{2}\theta -1),$$

where

5 $$p=\frac{{\mu }_{0}{\gamma }_{{{{\rm{H}}}}}{\gamma }_{{{{\rm{N}}}}}h}{16{\pi }^{2}\sqrt{2}{r}^{3}},$$

6 $${\delta }_{{{{\rm{N}}}}}=\frac{{\gamma }_{{{{\rm{N}}}}}{B}_{0}\Delta {\delta }_{{{{\rm{N}}}}}}{3\sqrt{2}},$$

7 $$J(\omega )=\frac{2{\tau }_{{{{\rm{c}}}}}}{5[1+{({\tau }_{{{{\rm{c}}}}}\omega )}^{2}]},$$

and *μ*_0_ = 1.27 × 10^−6^ H m^−1^, *γ*_H_ = 267.52 rad s^−1^ T^−1^, and *γ*_N_ = −27.12 rad s^−1^ T^−1^ are the gyromagnetic ratios of proton and nitrogen nuclei, respectively; *h* = 6.63 × 10^−34^ Js is Planck’s constant, Δ*δ*_N_ = 160 ppm, *r*_NH_ = 1.02 Å, *B*_0_ = 28.19 T, and *θ* = 17°.

Similarly^52^,

8 $${\tau }_{{{{\rm{c}}}}}=\frac{5{c}_{1}}{24}-\frac{336{\omega }_{{{{\rm{N}}}}}^{2}-25{c}_{1}^{2}{\omega }_{{{{\rm{N}}}}}^{4}}{24{\omega }_{{{{\rm{N}}}}}^{2}\,{\left(1800{c}_{1}{\omega }_{{{{\rm{N}}}}}^{4}+125{c}_{1}^{3}{\omega }_{{{{\rm{N}}}}}^{6}+24\sqrt{3}\,\sqrt{21952{\omega }_{{{{\rm{N}}}}}^{6}-3025{c}_{1}^{2}{\omega }_{{{{\rm{N}}}}}^{8}+625{c}_{1}^{4}{\omega }_{{{{\rm{N}}}}}^{10}}\right)}^{\frac{1}{3}}} \\ \quad+\frac{{\left(1800{c}_{1}{\omega }_{{{{\rm{N}}}}}^{4}+125{c}_{1}^{3}{\omega }_{{{{\rm{N}}}}}^{6}+24\sqrt{3}\,\sqrt{21952{\omega }_{{{{\rm{N}}}}}^{6}-3025{c}_{1}^{2}{\omega }_{{{{\rm{N}}}}}^{8}+625{c}_{1}^{4}{\omega }_{{{{\rm{N}}}}}^{10}}\right)}^{\frac{1}{3}}}{24{\omega }_{{{{\rm{N}}}}}^{2}},$$

where

9 $${c}_{1}=\frac{{\eta }_{{xy}}}{p{\delta }_{{{{\rm{N}}}}}(3{\cos }^{2}\theta -1)}.$$

Next, the rate constants *R*_1_ and *R*_2_ were calculated via Eqs. (10)–(15) as described by Palmer III.^24^,

10 $${R}_{1}=\frac{{d}_{2}^{2}}{4}(3J({\omega }_{{{{\rm{N}}}}})+J({\omega }_{{{{\rm{m}}}}})+6J({\omega }_{{{{\rm{p}}}}}))+{c}_{2}^{2}J({\omega }_{{{{\rm{N}}}}}),$$

where

11 $${c}_{2}=\frac{{\omega }_{{{{\rm{N}}}}}\Delta {\delta }_{{{{\rm{N}}}}}}{\sqrt{3}},$$

12 $${d}_{2}=\frac{{\mu }_{0} {\gamma }_{{\rm{H}}} {\gamma }_{{{{\rm{N}}}}}h} {8{\pi }^{2}{r}^{3}},$$

and ${\omega }_{{{{\rm{m}}}}}={\omega }_{{{{\rm{H}}}}}-{\omega }_{{{{\rm{N}}}}}$ and ${\omega }_{{{{\rm{p}}}}}={\omega }_{{{{\rm{H}}}}}+{\omega }_{{{{\rm{N}}}}}$, as well as

13 $${R}_{2}=	 \frac{{d}_{3}}{8}\left(4J\left(0\right)+3J\left({\omega }_{{{{\rm{H}}}}}\right)+J\left({\omega }_{{{{\rm{m}}}}}\right)+6J\left({\omega }_{{{{\rm{N}}}}}\right)+6J \big({\omega }_{{{{\rm{p}}}}}\big)\right) \\ 	+\frac{1}{6}{c}_{3}{\omega }_{{{{\rm{H}}}}}^{2}\left(4J\left(0\right)+3J\left({\omega }_{{{{\rm{H}}}}}\right)\right),$$

where

14 $${c}_{3}=\frac{\Delta {{{\rm{\delta }}}}^{2}_{{{\rm{H}}}}}{3},$$

15 $${d}_{3}={\left(\frac{{\mu }_{0}}{4{{{\rm{\pi }}}}}\right)}^{2}{\left(\frac{h}{2{{{\rm{\pi }}}}}\right)}^{2}{\gamma }_{{{{\rm{H}}}}}^{2}{\gamma }_{{{{\rm{N}}}}}^{2}{r}^{-6},$$

and Δ*δ*_H_ = 10^-5^ is the difference in the axially symmetric proton chemical shift tensor.

Finally, NOEs were calculated as described by Farrow et al.^72^,

16 $${{{\rm{NOE}}}}=1+\frac{{d}_{3}^{2}{\gamma }_{{{{\rm{H}}}}}}{4{\gamma }_{{{{\rm{N}}}}}}\left(6J\big({\omega }_{{{{\rm{p}}}}}\big)-J\left({\omega }_{{{{\rm{m}}}}}\right)\right)\frac{1}{{R}_{1}},$$

where *d*_*3*_ is defined in equation (15) and *R*_1_ in Eq. (10).

The code for the above calculations, as well as sample calculations and sample data, were deposited at the public repository GitHub https://github.com/dszollosi/p53_TAD_dynamics

### Fitting of 2-state CPMG model to RD profiles measured by NMR at 263 K

Characteristic timescales *τ* and their uncertainties were derived from NMR CPMG measurements at 263 K via Bayesian inference. The posterior distribution was estimated by Monte Carlo sampling (4000 samples) using the pymc5 package^125^ (version 5.15.0) with Python 3.11.11. Posterior probabilities were calculated from the probabilities of the set of RD measurements for CPMG profiles described by Eq. (17) using a Gaussian experimental error distribution, the width σ of which was also subject to inference. For some of the residues of the P27A mutant (e.g., Phe19 or Asp21, see Supplementary Information Fig. 1), the RD profile indicated the presence of two relaxation processes with markedly different timescales. For these residues, a sum of two CPMG profiles with a single *R*_2,0_ offset was estimated, Eq. (18). For σ and *R*_2,0_, a uniform prior with limits from 0 to the maximum of the RD profiles was used. For *Φ* and *τ*, log-uniform priors with boundaries of 10^1^−10^9^ 1/s^2^ and 10^−8^–10^−4.5^ s, respectively were used; for profiles that indicated a second process the additional *Φ* parameter used the same boundaries while *τ*_2_ was searched between 10^−4.5^ and 10^−3^ s. Estimated experimental errors (see 10.17617/3.JWVPWJ) were found to be similar in magnitude to the posterior distribution for σ.

17 $${R}_{2,{{{\rm{eff}}}}}\left(\nu \right)={R}_{{{\mathrm{2,0}}}}+\phi \tau \left(1-4\nu \tau \,\tanh \frac{1}{4\nu \tau }\right),$$

18 $${R}_{2,{{{\rm{eff}}}}}\left(\nu \right)={R}_{{{\mathrm{2,0}}}}+{\phi }_{1}{\tau }_{1}\left(1-4\nu {\tau }_{1}\,\tanh \frac{1}{4\nu {\tau }_{1}}\right)+{\phi }_{2}{\tau }_{2}\left(1-4\nu {\tau }_{2}\,\tanh \frac{1}{4\nu {\tau }_{2}}\right) . $$

### Fitting of 2-state CPMG model and stretched CPMG model to RD profiles calculated from MD simulations

To compare our NMR CPMG measurements with our MD simulations not only in terms of the raw spectra (see above), but also in terms of the characteristic exchange dynamics timescales *τ*, the analytical form of CPMG profiles for a two-state model (Eq. (19))^55,126^

19 $${R}_{2,{{{\rm{MD}}}}}\left(\nu \right)=\phi \tau \left(1-4\nu \tau \,\tanh \frac{1}{4\nu \tau }\right),$$

was fitted both to the measured NMR RD spectra as well as to those calculated from our MD simulations. Here, *v* is the CPMG frequency, *Φ* = *p*_A_ *p*_B_ ΔCS^2^ is the population weighted chemical shift variance, *p*_A_ and *p*_B_ are the populations of the two states A and B of the model, respectively, and ΔCS is the chemical shift difference between these two states.

Inspired by the shape of the RD profiles calculated from the simulations (cf. Supplementary Information Fig. 4), which in the logarithmic plot appears to be ‘stretched’ in the frequency domain relative to the above CPMG two-state model, a generalised model in terms of a ‘stretched’ function was considered,

20 $${R}_{2,{{{\rm{MD}}}}}\left(\nu \right)=\phi \tau \left(1-{\left(4\nu \tau \right)}^{\gamma }\,\tanh \frac{1}{{\left(4\nu \tau \right)}^{\gamma }}\right),$$

similar in spirit to the ‘stretched exponential functions’ used by Frauenfelder to describe the multi-tier dynamics of folded proteins^5,66,127^. Salvi et al.^67^ used a weighted superposition of *R*_2_ transverse relaxation rates with discrete timescale (fast, intermediate, slow). This is an alternative approach to include a broad range of characteristic timescale which we here described by the additional fitting parameter *γ* in Eq. (20). Accordingly, the stretched CPMG profile can be interpreted as a weighted superposition of many two-state CPMG profiles with a broad range of characteristic timescales, which is described by the additional fitting parameter *γ*. Therefore, *γ* also characterises the distribution width of barrier heights governing p53-TAD dynamics, namely *γ* = 1 indicates a two-state process and the smaller the value the broader the timescale distribution is. Fits of Eqs. (19) and (20) to either measured or calculated RD profiles were performed using a Bayesian approach^128^ using the pymc5 Python package^125^, with *τ* and *Φ* (and, additionally, *γ* for Eq. (20)) as free parameters to be determined by the fits.

The fact that all RD profiles were calculated by averaging over 30 individual RD profiles, each calculated from an absolute-squared Fourier transform of chemical shift trajectories as described above, required particular attention. Notably, Fourier transforms calculated numerically from finite time series are notoriously noisy, and the distribution of each individual (absolute squared) Fourier coefficient *R*_*k*_:= *R*_2_(*v*_*k*_) for realisations with a uniform distribution of random phases follows an exponential function^129^,

21 $$p\left({R}_{k}\right)=\frac{1}{{R}_{k}^{0}}{e}^{-\frac{{R}_{k}}{{R}_{k}^{0}}},$$

where $p({R}_{k}^{0})$ is the true coefficient from which the realisations were drawn. Hence, the probability distribution of the mean ${\bar{R}}_{k}$ of *N* = 30 Fourier transforms of realisations of the same process (the phases of which are assumed to be statistically independent and uniformly distributed) follows a gamma distribution

22 $$p\left({\bar{R}}_{k}\right)=\frac{{{{{\beta }}}}^{N}{\bar{R}}_{k}^{N-1}{e}^{-\beta {\bar{R}}_{k}}}{\Gamma \left(N\right)},$$

where $$\beta=\frac{N}{{\bar{R}}_{k}^{0}}$$.

Accordingly, this probability distribution was used (instead of a Gaussian distribution) for the Bayesian inference of each Fourier coefficient, with the fitting target ${\bar{R}}_{k}$. A log-uniform prior distribution between 10^3^–10^9^ Hz^2^ was used for *Φ*, a log-uniform prior between 1 ns–100 μs for *τ*, and a uniform prior between 0–2 for *γ*. An additional Jupyter notebook describes in detail all fitting steps, available at the public repository GitHub https://github.com/dszollosi/p53_TAD_dynamics.

For the residues 49ASP, 54PHE, 56GLU, 57ASP, 62GLU, 63ALA, and 66MET, the RD profile does not reach a plateau at low frequencies, indicating very slow dynamics (cf. the respective panels in Supplementary Information Figs. 4 and 5) which are also apparent from the Bayes posteriors. For these residues, therefore, no relaxation timescales were derived from the fits, which are therefore also not shown in Fig. 2B.

For an illustrative example comparing the different fitting models described above, see Supplementary Information Fig. 6 and the respective Supplementary Information Section.

### Markov state model analysis

The structural dynamics of the p53-TAD WT helix 1 and helix 2 comprising residues 18–26 and 40–53 respectively, were analysed by building a Markov state model from a dimension-reduced representation of the 600,000 available structure snapshots using the Python library “Deeptime”^130^ version 0.4.4. To extract the Markov states from the MD simulations, clustering was performed on the combined 600 μs trajectory set using the WT backbone dihedral angles *φ* and *ψ* of all helix 1 residues and snapshots from the MD trajectories recorded every nanosecond. For the clustering, sine and cosine values of these angles were used to avoid periodicity discontinuities, such that each residue is described/characterised by sin(*φ*), cos(*φ*), sin(*ψ*), and cos(*ψ*). Using these internal coordinates, super-positioning of snapshots of this IDP was avoided, which would have been challenging.

Dimension reduction of the resulting structure vectors was performed using time-lagged independent component analysis (TICA)^65^, with a lag time of 10 ns. The first three independent components were used as input for subsequent KMeans clustering^131^ (using the Deeptime implementation requesting 150 clusters, init_strategy was ‘kmeans++‘, max_iter = 500, and fixed_seed = 13). This clustering served to assign all simulation frames to microstates, which served as input for building a hidden Markov model requesting 15 initial macrostates. These 15 initial macrostates were merged heuristically into a final number of seven macrostates, chosen empirically by inspecting spatial proximity of average structures in the TICA projection and also considering the number of α-helix stabilising hydrogen bonds, to extract structurally and chemically unique and distinct conformations (Table 1). Hydrogen bonds stabilising an α-helix or a 3_10_-helix were defined by donor-acceptor distance *d* (in Å) according to the empirical Espinosa hydrogen bonds energy estimate^132^ (in kJ/mol) *E*_HB_ = −25,300*e*^−3.6*d*^ with a cut-off hydrogen bond strength of 1 *k*_B_*T*. As a result, hydrogen bonds were counted for all donor-acceptor pairs closer than *d* = 0.256 nm.

**Table 1:** Combining initial into final Markov states

Initial state	Final state
13	1
15	2
8	3
7, 9, 12	4
10, 14	5
1, 2, 4, 6	6
3, 5, 11	7

A total of 15 initial macrostates (left column) were combined into seven final macrostates (right column) for p53-TAD WT helix 1.

### Identification of metastable tertiary structure elements

p53-TAD transiently forms tertiary structures more complex than single α-helices or β-sheets. To detect those tertiary structures that last longer than 1 μs and therefore contribute to the overall structural ensemble, we performed a C_α_-C_α_ distance fluctuation analysis on our MD trajectories (both for the WT and for the P27A mutant), which uses internal coordinates and therefore does not require orientational fitting. Specifically, for every trajectory frame, intramolecular distances between all pairs of C_α_ atoms were calculated. Residue pairs separated by no or only one residue along the sequence were excluded from this analysis, because their mutual C_α_-C_α_ distances showed only small fluctuations and thus do not provide much information on metastable tertiary structures.

For all trajectories, the size of the distance fluctuations was quantified over time and for each residue pair via standard deviations, calculated by averaging over a sliding window of 100 ns width (containing 1000 frames). For each window position in time, and for each residue, the standard deviation of the distances to all other residues was calculated and then sorted. From the obtained list of standard deviations, the 10^th^ smallest one, indicating the 10^th^ least fluctuating distance, was used as a tertiary structure indicator. Here, the 10^th^ distance was chosen empirically, to best reflect tertiary structures larger than a typical two-turn α-helix or a smaller β-sheet. Of note, this indicator was also chosen because it does not require the involved residues to be on a continuous sequence segment and, therefore, it also identifies folds formed by distant residues.

Figure 4A shows an example of the resulting fluctuation map, showing averaged distance fluctuations between 0.1 nm (yellow) and 0.5 nm (dark blue), plotted for every residue (y-axis) over time (x-axis). In this plot, regions extending vertically over several residues and horizontally over more than 1 μs are easily identified, e.g., the two large yellow regions involving residues 15–53 lasting from 2–6 μs, and the one involving residues 47–66 between 20 and 24 μs. This analysis also recovers the faster tier 1 folding dynamics of the two helices of p53-TAD, which are sufficiently stable to show up as rapidly fluctuating yellow bands between residues 18–26 (helix 1) and residues 40–53 (helix 2). For an automated scan of all MD trajectories, a fluctuation cutoff of 0.15 nm was chosen, and 93 transient tertiary structure elements were identified lasting longer than 1 μs.

One might expect the transient tertiary structures indicated by the MD simulations and tier 0 dynamics to be detected on a NOESY spectrum. The fact that not one but many different transient tertiary structures are seen also explains why these are not seen as peaks in the NOESY spectra. Indeed, inspection of the expected positions in measured NOESY spectra (see “Methods” subsection “NMR relaxation measurements”) did not reveal any signals beyond the noise level. Although the population of tertiary structures, taken together, is large enough to evoke a low-frequency component in the RD profiles at least for P27A, the population of each of the many different structures is below 0.3%, whereas an estimated population of at least 1% would be required to generate a visible NOESY cross-peak.

### Analyses and comparison to polymer models

The extensive sampling provided by our atomistic simulations of the p53-TAD also allowed us to characterise the local fast (tier 2) dynamics of this IDP from a polymer model perspective, e.g., in terms of persistent lengths and end-to-end distances, which for a polymer chain—and depending on the polymer model used—are connected^133^. For, e.g., a wormlike chain model, the (average squared) end-to-end distance *R*_e_ reads

23 $$\big\langle {R}_{e}^{2}\big\rangle=2{l}_{{{{\rm{p}}}}}L\left(1-\frac{{l}_{{{{\rm{p}}}}}}{L}\left(1-{e}^{-\frac{L}{{l}_{{{{\rm{p}}}}}}}\right)\right),$$

where *l*_p_ is the persistence length, *L* = *bN* is the contour length, i.e., the length of one polymer unit (*b* = 0.38 nm) multiplied by their number *N*. From our MD trajectories, $\big\langle {R}_{e}^{2}\big\rangle$ was calculated by time- and trajectory-averaging over all distances between all pairs of C_α_ atoms separated by given contour length (i.e., number of residues). Fitting Eq. (23) to the resulting curve (Supplementary Information Fig. 19A) yielded a persistence length of *l*_*p*_ = 1.0 nm.

Residue-specific persistence lengths were obtained similarly, except that averaging over different sequence positions was omitted, such that $\big\langle {R}_{e}^{2}\big\rangle$ was obtained as a function of ‘start’ residue. Similar fits to Eq. (23) as above provided residue-position-resolved persistence length, characterising deviations from a simple homopolymer (Supplementary Information Fig. 19B). As can be seen, shorter persistence lengths *l*_*p*_ were obtained for proline rich segments, e.g. Pro12-Pro13.

We also characterised the relation between persistence length *l*_p_ and radius of gyration *R*_g_, which is given by the polymer scaling law^77^

24 $$\big\langle {R}_{{{{\rm{g}}}}}\big\rangle=\sqrt{\frac{2{l}_{{{{\rm{p}}}}}b}{\left(2{{{\rm{\nu }}}}+1\right)\left(2{{{\rm{\nu }}}}+2\right)}}{N}^{{{{\rm{\nu }}}}},$$

where *N* is the number of residues in the different tested segments, *l*_p_ and *b* are as defined above, and *v* is the scaling exponent. A value of *v* = 0.5 indicates a ‘Flory random coil’^134^, smaller values a compact and larger values a more extended ensemble. Similarly, as for $\big\langle {R}_{e}^{2}\big\rangle$, *R*_g_ was calculated for all protein segments of length between *N* = 6 and 30 residues, averaging over time, trajectories, and all possible segment positions in the peptide. From the fit shown in Supplementary Information Fig. 19C a markedly shorter persistence length of 0.31 nm and a scaling exponent of 0.66 was obtained; the latter indicating an expanded coil state^77^, similar to an excluded volume chain model (0.588)^135^. For comparison, *R*_g_ of the full protein was calculated similarly, but was not used for the above fit.

Alternatively, the persistence length was also estimated (Supplementary Information Fig. 19D) by fitting single and double exponential functions, respectively, to the normalised orientation autocorrelation function of the vectors connecting C_α_ atoms separated by *N* > 2 residues^133^,

25 $$C\left(N\right)=A{e}^{-\frac{{Nb}}{{l}_{{{{\rm{p}}}},1}}}+\left(1-A\right){e}^{-\frac{{Nb}}{{l}_{{{{\rm{p}}}},2}}},$$

where *A* is a prefactor, *N* is the number of polymer units (residues) separating the vectors, with the same unit length *b* = 0.38 nm as above. Persistence length (*l*_p_) from the single exponential fit is 1.19 ± 0.01 nm, whereas from the double exponential 0.84 ± 0.01 nm and 5.06 ± 0.26 nm, for *l*_p,1_ and *l*_p,2_, respectively.

The fast reorientation tier 2 dynamics were also characterised by the running average root mean squared deviation (RMSD)

26 $${{{\rm{RMSD}}}}\left({{{\tau }}}\right)={\sum }_{i=1}^{{N}_{{{{\rm{atoms}}}}}}\sqrt{\frac{1}{{N}_{{{{\rm{atoms}}}}}}{\left({x}_{i}\left(t\right)-{x}_{i}\left(t+{{{\tau }}}\right)\right)}^{2}},$$

averaged over all trajectories (Supplementary Information Fig. 19E). With increasing lag time, and for ca. 10 ns over nearly two timescale decades, the structural deviation increase closely follows a power law with an exponent of 0.39; thereafter the RMSD saturates. This reorganisation time of ca 10 ns agrees with that measured for other polymers by single-molecule FRET^15^.

Complementing this analysis of structural dynamics at a rather detailed level, we finally calculated the autocorrelation times of the radius of gyration *R*_g_ as well as of the end-to-end distance of the full p53-TAD chain. Fitting a double exponential function

27 $$C\left(\tau \right)=A{e}^{-\frac{\tau }{{\tau }_{1}}}+\left(1-A\right){e}^{-\frac{\tau }{{\tau }_{2}}}$$

to the respective autocorrelation functions that were calculated similarly as above, two timescale values (*τ*_1_ and *τ*_2_) were obtained for each of the two observables (Supplementary Information Fig. 19F), namely 22.0 and 406.8 ns for *R*_*g*_ and 18.5 and 349.8 ns for the end-to-end distance.

### Reporting summary

Further information on research design is available in the Nature Portfolio Reporting Summary linked to this article.

## Supplementary information

Supplementary Information

Description of Additional Supplementary Files

Supplementary Movie 1

Supplementary Movie 2

Supplementary Movie 3

Reporting Summary

Transparent Peer Review file

## Data Availability

Supplementary Movies 1–3 have been deposited at https://github.com/dszollosi/p53_TAD_dynamics [10.5281/zenodo.19704215]^136^. All NMR spectra have been deposited at https://edmond.mpg.de/dataset.xhtml?persistentId=doi:10.17617/3.JWVPWJ, 10.17617/3.JWVPWJ. All molecular dynamics simulation starting structures, simulation parameters, and the force field were deposited to the same repository, as well as coarse-sampled trajectories. The full molecular dynamics simulation trajectories are available from H.G. upon request; requests will be answered within 1 month. These data have not been deposited due to large data size ( > 1 TByte). The following Biological Magnetic Resonance Bank entry was used: BMRB 17760 (backbone resonance assignments of p53 N-terminal transactivation domain (1–93)). The following Protein Data Bank entries were used: PDB 2ESX (V3 region of gp120 of the JR-FL HIV-1 strain); PDB 2BTB (NMR STUDY OF N-TERMINAL HUMAN BAND 3 PEPTIDE, RESIDUES 1–15); PDB 8Y3S (Human Keratin 19 head domain segment G28–G38 in solution); PDB 5MWP (The structure of MR in complex with AZD9977). Source Data have been deposited at https://edmond.mpg.de/dataset.xhtml?persistentId=doi:10.17617/3.JWVPW.

## Code availability

The program code used for all calculations, additional sample calculations, and sample data has been deposited at https://github.com/dszollosi/p53_TAD_dynamics [10.5281/zenodo.19704215]^136^.

## References

[1] Karplus, M. & McCammon, J. A. Dynamics of proteins: elements and function. *Annu. Rev. Biochem.* **52**, 263–300 (1983). 6351724 10.1146/annurev.bi.52.070183.001403

[2] Henzler-Wildman, K. & Kern, D. Dynamic personalities of proteins. *Nature* **450**, 964–972 (2007). 18075575 10.1038/nature06522

[3] Smith, C. A. et al. Allosteric switch regulates protein–protein binding through collective motion. *Proc. Natl. Acad. Sci. USA* **113**, 3269–3274 (2016). 26961002 10.1073/pnas.1519609113 PMC4812760

[4] Boehr, D. D., Nussinov, R. & Wright, P. E. The role of dynamic conformational ensembles in biomolecular recognition. *Nat. Chem. Biol.* **5**, 789–796 (2009). 19841628 10.1038/nchembio.232 PMC2916928

[5] Frauenfelder, H., Sligar, S. G. & Wolynes, P. G. The energy landscapes and motions of proteins. *Science* **254**, 1598–1603 (1991). 1749933 10.1126/science.1749933

[6] Smith, C. A. et al. Population shuffling of protein conformations. *Angew. Chem. Int. Ed.* **54**, 207–210 (2015). 10.1002/anie.201408890 25377083

[7] Onuchic, J. N., Wolynes, P. G., Luthey-Schulten, Z. & Socci, N. D. Toward an outline of the topography of a realistic protein-folding funnel. *Proc. Natl. Acad. Sci. USA* **92**, 3626–3630 (1995). 7724609 10.1073/pnas.92.8.3626 PMC42220

[8] Frauenfelder, H. et al. A unified model of protein dynamics. *Proc. Natl. Acad. Sci. USA* **106**, 5129–5134 (2009). 19251640 10.1073/pnas.0900336106 PMC2649210

[9] Dyson, H. J. & Wright, P. E. Intrinsically unstructured proteins and their functions. *Nat. Rev. Mol. Cell Biol.* **6**, 197–208 (2005). 15738986 10.1038/nrm1589

[10] Abyzov, A. et al. Identification of dynamic modes in an intrinsically disordered protein using temperature-dependent NMR relaxation. *J. Am. Chem. Soc.* **138**, 6240–6251 (2016). 27112095 10.1021/jacs.6b02424

[11] Wright, P. E. & Dyson, H. J. Intrinsically disordered proteins in cellular signalling and regulation. *Nat. Rev. Mol. Cell Biol.* **16**, 18–29 (2015). 25531225 10.1038/nrm3920 PMC4405151

[12] Lee, H. et al. Local structural elements in the mostly unstructured transcriptional activation domain of human p53. *J. Biol. Chem.* **275**, 29426–29432 (2000). 10884388 10.1074/jbc.M003107200

[13] Bernadó, P., Bertoncini, C. W., Griesinger, C., Zweckstetter, M. & Blackledge, M. Defining long-range order and local disorder in native α-synuclein using residual dipolar couplings. *J. Am. Chem. Soc.* **127**, 17968–17969 (2005). 16366524 10.1021/ja055538p

[14] Kulkarni, P. et al. Intrinsically disordered proteins: ensembles at the limits of Anfinsen’s dogma. *Biophys. Rev*. **3**, 011306 (2022). 10.1063/5.0080512 PMC10903413 38505224

[15] Nettels, D., Hoffmann, A. & Schuler, B. Unfolded protein and peptide dynamics investigated with single-molecule FRET and correlation spectroscopy from picoseconds to seconds. *J. Phys. Chem. B* **112**, 6137–6146 (2008). 18410159 10.1021/jp076971j

[16] Schuler, B., Lipman, E. A. & Eaton, W. A. Probing the free-energy surface for protein folding with single-molecule fluorescence spectroscopy. *Nature* **419**, 743–747 (2002). 12384704 10.1038/nature01060

[17] Burger, V. M., Gurry, T. & Stultz, C. M. Intrinsically disordered proteins: where computation meets experiment. *Polymers* **6**, 2684–2719 (2014).

[18] Muhammedkutty, F. N. K., MacAinsh, M. & Zhou, H.-X. Atomistic molecular dynamics simulations of intrinsically disordered proteins. *Curr. Opin. Struct. Biol.* **92**, 103029 (2025). 40068541 10.1016/j.sbi.2025.103029 PMC12360891

[19] Papoian, G. A. Proteins with weakly funneled energy landscapes challenge the classical structure–function paradigm. *Proc. Natl. Acad. Sci. USA* **105**, 14237–14238 (2008). 18799750 10.1073/pnas.0807977105 PMC2567144

[20] Chowdhury, A., Nettels, D. & Schuler, B. Interaction dynamics of intrinsically isordered proteins from single-molecule spectroscopy. *Annu. Rev. Biophys.* **52**, 433–462 (2023). 36750251 10.1146/annurev-biophys-101122-071930

[21] Cubuk, J., Stuchell-Brereton, M. D. & Soranno, A. The biophysics of disordered proteins from the point of view of single-molecule fluorescence spectroscopy. *Essays Biochem.* **66**, 875–890 (2022). 36416865 10.1042/EBC20220065 PMC9760427

[22] Salvi, N. et al. Convergent views on disordered protein dynamics from NMR and computational approaches. *Biophys. J.* **121**, 3785–3794 (2022). 36131545 10.1016/j.bpj.2022.09.016 PMC9674986

[23] Vise, P. D., Baral, B., Latos, A. J. & Daughdrill, G. W. NMR chemical shift and relaxation measurements provide evidence for the coupled folding and binding of the p53 transactivation domain. *Nucleic Acids Res.* **33**, 2061–2077 (2005). 15824059 10.1093/nar/gki336 PMC1075921

[24] Palmer, A. G. III. NMR characterization of the dynamics of biomacromolecules. *Chem. Rev.* **104**, 3623–3640 (2004). 15303831 10.1021/cr030413t

[25] Kodera, N. et al. Structural and dynamics analysis of intrinsically disordered proteins by high-speed atomic force microscopy. *Nat. Nanotechnol.* **16**, 181–189 (2021). 33230318 10.1038/s41565-020-00798-9

[26] Herrera-Nieto, P., Pérez, A. & De Fabritiis, G. Characterization of partially ordered states in the intrinsically disordered N-terminal domain of p53 using millisecond molecular dynamics simulations. *Sci. Rep.* **10**, 12402 (2020). 32709860 10.1038/s41598-020-69322-2 PMC7382488

[27] Best, R. B. Computational and theoretical advances in studies of intrinsically disordered proteins. *Curr. Opin. Struct. Biol.* **42**, 147–154 (2017). 28259050 10.1016/j.sbi.2017.01.006

[28] van der Lee, R. et al. Classification of intrinsically disordered regions and proteins. *Chem. Rev.* **114**, 6589–6631 (2014). 24773235 10.1021/cr400525m PMC4095912

[29] Graen, T., Klement, R., Grupi, A., Haas, E. & Grubmüller, H. Transient secondary and tertiary structure formation kinetics in the intrinsically disordered state of α-synuclein from atomistic simulations. *ChemPhysChem* **19**, 2507–2511 (2018). 30047198 10.1002/cphc.201800504

[30] Löhr, T., Kohlhoff, K., Heller, G. T., Camilloni, C. & Vendruscolo, M. A kinetic ensemble of the Alzheimer’s Aβ peptide. *Nat. Comput. Sci.* **1**, 71–78 (2021). 38217162 10.1038/s43588-020-00003-w

[31] Mollaei, P., Sadasivam, D., Guntuboina, C. & Barati Farimani, A. IDP-Bert: predicting properties of intrinsically disordered proteins using large language models. *J. Phys. Chem. B* **128**, 12030–12037 (2024). 39586094 10.1021/acs.jpcb.4c02507 PMC11647883

[32] Gupta, A., Dey, S., Hicks, A. & Zhou, H.-X. Artificial intelligence guided conformational mining of intrinsically disordered proteins. *Commun. Biol.* **5**, 610 (2022). 35725761 10.1038/s42003-022-03562-y PMC9209487

[33] Soranno, A. et al. Quantifying internal friction in unfolded and intrinsically disordered proteins with single-molecule spectroscopy. *Proc. Natl. Acad. Sci. USA* **109**, 17800–17806 (2012). 22492978 10.1073/pnas.1117368109 PMC3497802

[34] Levine, A. J. p53: 800 million years of evolution and 40 years of discovery. *Nat. Rev. Cancer* **20**, 471–480 (2020). 32404993 10.1038/s41568-020-0262-1

[35] Kussie, P. H. et al. Structure of the MDM2 oncoprotein bound to the p53 tumor suppressor transactivation domain. *Science* **274**, 948–953 (1996). 8875929 10.1126/science.274.5289.948

[36] Watson, M. & Stott, K. Disordered domains in chromatin-binding proteins. *Essays Biochem.* **63**, 147–156 (2019). 30940742 10.1042/EBC20180068

[37] Chi, S.-W. et al. Structural details on MDM2-p53 Interaction. *J. Biol. Chem.* **280**, 38795–38802 (2005). 16159876 10.1074/jbc.M508578200

[38] Wei, X. et al. Secondary interaction between MDMX and p53 core domain inhibits p53 DNA binding. *Proc. Natl. Acad. Sci. USA* **113**, E2558–E2563 (2016). 27114532 10.1073/pnas.1603838113 PMC4868482

[39] Sun, X., Dyson, H. J. & Wright, P. E. A phosphorylation-dependent switch in the disordered p53 transactivation domain regulates DNA binding. *Proc. Natl. Acad. Sci. USA* **118**, e2021456118 (2021). 33443163 10.1073/pnas.2021456118 PMC7817127

[40] Crabtree, M. D. et al. Conserved helix-flanking prolines modulate intrinsically disordered protein:target affinity by altering the lifetime of the bound complex. *Biochemistry* **56**, 2379–2384 (2017). 28425697 10.1021/acs.biochem.7b00179 PMC5467178

[41] Higbee, P. S., Dayhoff, G. W., Anbanandam, A., Varma, S. & Daughdrill, G. Structural adaptation of secondary p53 binding sites on MDM2 and MDMX. *J. Mol. Biol.* **436**, 168626 (2024). 38810774 10.1016/j.jmb.2024.168626 PMC12624572

[42] Zwier, M. C. et al. Efficient atomistic simulation of pathways and calculation of rate constants for a protein–peptide binding process: application to the MDM2 protein and an intrinsically disordered p53 peptide. *J. Phys. Chem. Lett.* **7**, 3440–3445 (2016). 27532687 10.1021/acs.jpclett.6b01502 PMC5008990

[43] Borcherds, W. et al. Disorder and residual helicity alter p53-MDM2 binding affinity and signaling in cells. *Nat. Chem. Biol.* **10**, 1000–1002 (2014). 25362358 10.1038/nchembio.1668

[44] Lange, O. F. et al. Recognition dynamics up to microseconds revealed from an RDC-derived Ubiquitin ensemble in solution. *Science* **320**, 1471–1475 (2008). 18556554 10.1126/science.1157092

[45] Mukhopadhyay, D., Pratihar, S., Becker, S. & Griesinger, C. Extending the detectable time window of fast protein dynamics using 1HN E-CPMG. *J. Biomol. NMR* **79**, 231–241 (2025). 10.1007/s10858-025-00470-1 PMC12664843 40586990

[46] Skalicky, J. J., Sukumaran, D. K., Mills, J. L. & Szyperski, T. Toward structural biology in supercooled water. *J. Am. Chem. Soc.* **122**, 3230–3231 (2000).

[47] Evans, M. G. & Polanyi, M. Some applications of the transition state method to the calculation of reaction velocities, especially in solution. *Trans. Faraday Soc.* **31**, 875–894 (1935).

[48] Eyring, H. The activated complex in chemical reactions. *J. Chem. Phys.* **3**, 107–115 (1935).

[49] Lum, J. K., Neuweiler, H. & Fersht, A. R. Long-range modulation of chain motions within the intrinsically disordered transactivation domain of tumor suppressor p53. *J. Am. Chem. Soc.* **134**, 1617–1622 (2012). 22176582 10.1021/ja2078619 PMC3265989

[50] Kizilsavas, G., Ledolter, K. & Kurzbach, D. Hydrophobic collapse of the intrinsically disordered transcription tactor Myc associated factor X. *Biochemistry* **56**, 5365–5372 (2017). 28880537 10.1021/acs.biochem.7b00679

[51] Zondlo, S. C., Lee, A. E. & Zondlo, N. J. Determinants of specificity of MDM2 for the activation domains of p53 and p65: proline27 disrupts the MDM2-binding motif of p53. *Biochemistry* **45**, 11945–11957 (2006). 17002294 10.1021/bi060309g

[52] Robson, S. A., Dağ, Ç, Wu, H. & Ziarek, J. J. TRACT revisited: an algebraic solution for determining overall rotational correlation times from cross-correlated relaxation rates. *J. Biomol. NMR* **75**, 293–302 (2021). 34480265 10.1007/s10858-021-00379-5 PMC8627365

[53] Shrestha, U. R. et al. Generation of the configurational ensemble of an intrinsically disordered protein from unbiased molecular dynamics simulation. *Proc. Natl. Acad. Sci. USA* **116**, 20446–20452 (2019). 31548393 10.1073/pnas.1907251116 PMC6789927

[54] Wells, M. et al. Structure of tumor suppressor p53 and its intrinsically disordered N-terminal transactivation domain. *Proc. Natl. Acad. Sci. USA* **105**, 5762–5767 (2008). 18391200 10.1073/pnas.0801353105 PMC2311362

[55] Musselman, C., Zhang, Q., Al-Hashimi, H. & Andricioaei, I. Referencing strategy for the direct comparison of nuclear magnetic resonance and molecular dynamics motional parameters in RNA. *J. Phys. Chem. B* **114**, 929–939 (2010). 20039757 10.1021/jp905286h PMC4287414

[56] Allerhand, A. & Thiele, E. Analysis of Carr-Purcell spin-echo NMR experiments on multiple-spin systems. II. The effect of chemical exchange. *J. Chem. Phys.* **45**, 902–916 (1966).

[57] Marsh, J. A., Singh, V. K., Jia, Z. & Forman-Kay, J. D. Sensitivity of secondary structure propensities to sequence differences between α- and γ-synuclein: implications for fibrillation. *Protein Sci.* **15**, 2795–2804 (2006). 17088319 10.1110/ps.062465306 PMC2242444

[58] Rauscher, S. et al. Structural ensembles of intrinsically disordered proteins depend strongly on force field: a comparison to experiment. *J. Chem. Theory Comput.* **11**, 5513–5524 (2015). 26574339 10.1021/acs.jctc.5b00736

[59] Huang, J. et al. CHARMM36m: an improved force field for folded and intrinsically disordered proteins. *Nat. Methods* **14**, 71–73 (2017). 27819658 10.1038/nmeth.4067 PMC5199616

[60] Robustelli, P., Piana, S. & Shaw, D. E. Developing a molecular dynamics force field for both folded and disordered protein states. *Proc. Natl. Acad. Sci. USA* **115**, E4758–E4766 (2018). 29735687 10.1073/pnas.1800690115 PMC6003505

[61] Shen, Y. & Bax, A. SPARTA+: a modest improvement in empirical NMR chemical shift prediction by means of an artificial neural network. *J. Biomol. NMR* **48**, 13–22 (2010). 20628786 10.1007/s10858-010-9433-9 PMC2935510

[62] Han, B., Liu, Y., Ginzinger, S. W. & Wishart, D. S. SHIFTX2: significantly improved protein chemical shift prediction. *J. Biomol. NMR* **50**, 43–57 (2011). 21448735 10.1007/s10858-011-9478-4 PMC3085061

[63] Wong, T. R., Rutherford, T., Freund, S. & Fersht, A. BMRB Entry 17760: backbone resonance assignments of p53 N-terminal transactivation domain (1-93). https://bmrb.io/data_library/summary/?bmrbId=17760 (2011).

[64] Craveur, P., Joseph, A. P., Poulain, P., de Brevern, A. G. & Rebehmed, J. Cis–trans isomerization of omega dihedrals in proteins. *Amino Acids* **45**, 279–289 (2013). 23728840 10.1007/s00726-013-1511-3

[65] Molgedey, L. & Schuster, H. G. Separation of a mixture of independent signals using time delayed correlations. *Phys. Rev. Lett.* **72**, 3634–3637 (1994). 10056251 10.1103/PhysRevLett.72.3634

[66] Edholm, O. & Blomberg, C. Stretched exponentials and barrier distributions. *Chem. Phys.* **252**, 221–225 (2000).

[67] Salvi, N., Abyzov, A. & Blackledge, M. Multi-timescale dynamics in intrinsically disordered proteins from NMR relaxation and molecular simulation. *J. Phys. Chem. Lett.* **7**, 2483–2489 (2016). 27300592 10.1021/acs.jpclett.6b00885

[68] van Kampen, N. G. *Stochastic Processes in Physics and Chemistry* 3rd edn (Elsevier, 2007).

[69] Lindorff-Larsen, K., Piana, S., Dror, R. O. & Shaw, D. E. How fast-folding proteins fold. *Science* **334**, 517–520 (2011). 22034434 10.1126/science.1208351

[70] Meinhold, D. W., Felitsky, D. J., Dyson, H. J. & Wright, P. E. Transient on- and off-pathway protein folding intermediate states characterized with NMR relaxation dispersion. *J. Phys. Chem. B* **126**, 9539–9548 (2022). 36354189 10.1021/acs.jpcb.2c05592 PMC9793904

[71] Peter, C., Daura, X. & van Gunsteren, W. F. Calculation of NMR-relaxation parameters for flexible molecules from molecular dynamics simulations. *J. Biomol. NMR* **20**, 297–310 (2001). 11563554 10.1023/a:1011241030461

[72] Farrow, N. A., Zhang, O., Szabo, A., Torchia, D. A. & Kay, L. E. Spectral density function mapping using 15N relaxation data exclusively. *J. Biomol. NMR* **6**, 153–162 (1995). 8589604 10.1007/BF00211779

[73] Sorin, E. J. & Pande, V. S. Exploring the helix-coil transition via all-atom equilibrium ensemble simulations. *Biophys. J.* **88**, 2472–2493 (2005). 15665128 10.1529/biophysj.104.051938 PMC1305346

[74] Abyzov, A., Mandelkow, E., Zweckstetter, M. & Rezaei-Ghaleh, N. Fast motions dominate dynamics of intrinsically disordered Tau protein at high temperatures. *Chem. Eur. J.* **29**, e202203493 (2023). 36579699 10.1002/chem.202203493

[75] Huang, F. et al. Multiple conformations of full-length p53 detected with single-molecule fluorescence resonance energy transfer. *Proc. Natl. Acad. Sci. USA* **106**, 20758–20763 (2009). 19933326 10.1073/pnas.0909644106 PMC2791586

[76] Hammouda, B. in *Polymer Characteristics* 87–133 (Springer, 1993).

[77] Hofmann, H. et al. Polymer scaling laws of unfolded and intrinsically disordered proteins quantified with single-molecule spectroscopy. *Proc. Natl. Acad. Sci. USA* **109**, 16155–16160 (2012). 22984159 10.1073/pnas.1207719109 PMC3479594

[78] de Gennes, P.-G. *Scaling Concepts in Polymer Physics* (Cornell University Press, 1979).

[79] Bourhis, J.-M. et al. The C-terminal domain of measles virus nucleoprotein belongs to the class of intrinsically disordered proteins that fold upon binding to their physiological partner. *Virus Res.* **99**, 157–167 (2004). 14749181 10.1016/j.virusres.2003.11.007

[80] Troilo, F., Bonetti, D., Bignon, C., Longhi, S. & Gianni, S. Understanding intramolecular crosstalk in an intrinsically disordered protein. *ACS Chem. Biol.* **14**, 337–341 (2019). 30715849 10.1021/acschembio.8b01055

[81] Otteson, L. et al. Transient non-local interactions dominate the dynamics of measles virus N-TAIL. *Commun. Chem.* **8**, 298 (2025). 41053265 10.1038/s42004-025-01682-0 PMC12501004

[82] Berman, H. M. et al. The Protein Data Bank. *Nucleic Acids Res.* **28**, 235–242 (2000). 10592235 10.1093/nar/28.1.235 PMC102472

[83] Granata, D. et al. The inverted free energy landscape of an intrinsically disordered peptide by simulations and experiments. *Sci. Rep.* **5**, 15449 (2015). 26498066 10.1038/srep15449 PMC4620491

[84] Shaw, D. E. et al. Atomic-level characterization of the structural dynamics of proteins. *Science* **330**, 341–346 (2010). 20947758 10.1126/science.1187409

[85] Fersht, A. R. & Sato, S. Φ-Value analysis and the nature of protein-folding transition states. *Proc. Natl. Acad. Sci. USA* **101**, 7976–7981 (2004). 15150406 10.1073/pnas.0402684101 PMC419542

[86] Bogomolovas, J., Simon, B., Sattler, M. & Stier, G. Screening of fusion partners for high yield expression and purification of bioactive viscotoxins. *Protein Expression Purif.* **64**, 16–23 (2009). 10.1016/j.pep.2008.10.003 18983922

[87] Mori, S., Abeygunawardana, C., Johnson, M. O. & Vanzijl, P. C. M. Improved sensitivity of HSQC spectra of exchanging protons at short interscan delays using a new fast HSQC (FHSQC) detection scheme that avoids water saturation. *J. Magn. Reson. Ser. B* **108**, 94–98 (1995). 7627436 10.1006/jmrb.1995.1109

[88] Xie, M., Hansen, A. L., Yuan, J. & Brüschweiler, R. Residue-specific interactions of an intrinsically disordered protein with silica nanoparticles and their quantitative prediction. *J. Phys. Chem. C* **120**, 24463–24468 (2016). 10.1021/acs.jpcc.6b08213 PMC5358802 28337243

[89] Delaglio, F. et al. NMRPipe: a multidimensional spectral processing system based on UNIX pipes. *J. Biomol. NMR* **6**, 277–293 (1995). 8520220 10.1007/BF00197809

[90] Lee, W., Tonelli, M. & Markley, J. L. NMRFAM-SPARKY: enhanced software for biomolecular NMR spectroscopy. *Bioinformatics* **31**, 1325–1327 (2014). 25505092 10.1093/bioinformatics/btu830 PMC4393527

[91] Lakomek, N. A., Ying, J. & Bax, A. Measurement of ¹⁵N relaxation rates in perdeuterated proteins by TROSY-based methods. *J. Biomol. NMR* **53**, 209–221 (2012). 22689066 10.1007/s10858-012-9626-5 PMC3412688

[92] Peng, J. W., Thanabal, V. & Wagner, G. 2D heteronuclear NMR measurements of spin-lattice relaxation times in the rotating frame of X nuclei in heteronuclear HX spin systems. *J. Magn. Reson.* **94**, 82–100 (1991).

[93] Pratihar, S. et al. Kinetics of the antibody recognition site in the third IgG-binding domain of Protein G. *Angew. Chem. Int. Ed.* **55**, 9567–9570 (2016). 10.1002/anie.201603501 27345359

[94] Eichmüller, C. & Skrynnikov, N. R. A new amide proton R1ρ experiment permits accurate characterization of microsecond time-scale conformational exchange. *J. Biomol. NMR* **32**, 281–293 (2005). 16211482 10.1007/s10858-005-0658-y

[95] Mulder, F. A. A., de Graaf, R. A., Kaptein, R. & Boelens, R. An off-resonance rotating frame relaxation experiment for the investigation of macromolecular dynamics using adiabatic rotations. *J. Magn. Reson.* **131**, 351–357 (1998). 9571112 10.1006/jmre.1998.1380

[96] Kupce, E. & Freeman, R. Adiabatic pulses for wideband inversion and broadband decoupling. *J. Magn. Reson. Ser. A* **115**, 273–276 (1995).

[97] Kay, L. E., Keifer, P. & Saarinen, T. Pure absorption gradient enhanced heteronuclear single quantum correlation spectroscopy with improved sensitivity. *J. Am. Chem. Soc.* **114**, 10663–10665 (1992).

[98] Schleucher, J., Sattler, M. & Griesinger, C. Coherence selection by gradients without signal attenuation: application to the three-dimensional HNCO experiment. *Angew. Chem. Int. Ed.* **32**, 1489–1491 (1993).

[99] Marion, D., Ikura, M., Tschudin, R. & Bax, A. Rapid recording of 2D NMR spectra without phase cycling. Application to the study of hydrogen exchange in proteins. *J. Magn. Reson.* **85**, 393–399 (1989).

[100] Lee, D., Hilty, C., Wider, G. & Wüthrich, K. Effective rotational correlation times of proteins from NMR relaxation interference. *J. Magn. Reson.* **178**, 72–76 (2006). 16188473 10.1016/j.jmr.2005.08.014

[101] Sieme, D. et al. Autoinhibition in the signal transducer CIN85 modulates B cell activation. *J. Am. Chem. Soc.* **146**, 399–409 (2024). 38111344 10.1021/jacs.3c09586 PMC10786037

[102] Goldman, M. Interference effects in the relaxation of a pair of unlike spin-12 nuclei. *J. Magn. Reson.* **60**, 437–452 (1984).

[103] Cavanagh, J., Fairbrother, W. J. & Palmer, A. G. I. *Protein NMR Spectroscopy: Principles and Practice* (Academic Press, 2006).

[104] Tjandra, N. & Bax, A. Solution NMR measurement of amide proton chemical shift anisotropy in 15N-enriched proteins. Correlation with hydrogen bond length. *J. Am. Chem. Soc.* **119**, 8076–8082 (1997).

[105] Yuwen, T. & Kay, L. E. Revisiting 1HN CPMG relaxation dispersion experiments: a simple modification can eliminate large artifacts. *J. Biomol. NMR* **73**, 641–650 (2019). 31646421 10.1007/s10858-019-00276-y

[106] Reddy, J. G. et al. Simultaneous determination of fast and slow dynamics in molecules using extreme CPMG relaxation dispersion experiments. *J. Biomol. NMR* **70**, 1–9 (2018). 29188417 10.1007/s10858-017-0155-0

[107] Ban, D. et al. Enhanced accuracy of kinetic information from CT-CPMG experiments by transverse rotating-frame spectroscopy. *J. Biomol. NMR* **57**, 73–82 (2013). 23949308 10.1007/s10858-013-9769-z PMC12885143

[108] Ban, D., Smith, C. A., de Groot, B. L., Griesinger, C. & Lee, D. Recent advances in measuring the kinetics of biomolecules by NMR relaxation dispersion spectroscopy. *Arch. Biochem. Biophys.* **628**, 81–91 (2017). 28576576 10.1016/j.abb.2017.05.016

[109] Ban, D., Sabo, T. M., Griesinger, C. & Lee, D. Measuring dynamic and kinetic information in the previously inaccessible supra-tc window of nanoseconds to microseconds by solution NMR spectroscopy. *Molecules* **18**, 11904–11937 (2013). 24077173 10.3390/molecules181011904 PMC6270068

[110] Brutscher, B. & Solyom, Z. in *Fast NMR Data Acquisition: Beyond the Fourier Transform* (eds Mobli, M. & Hoch, J. C.) 0 (The Royal Society of Chemistry, 2017).

[111] Keller, R. L. J. *The Computer Aided Resonance Assignment Tutorial* (CANTINA Verlag, 2004).

[112] Abraham, M. J. et al. GROMACS: high performance molecular simulations through multi-level parallelism from laptops to supercomputers. *SoftwareX* **1-2**, 19–25 (2015).

[113] Qiu, D., Shenkin, P. S., Hollinger, F. P. & Still, W. C. The GB/SA continuum model for solvation. A fast analytical method for the calculation of approximate Born radii. *J. Phys. Chem. A* **101**, 3005–3014 (1997).

[114] Berendsen, H. J. C. & Van Gunsteren, W. F. in *Molecular Liquids: Dynamics and Interactions* (eds Barnes, A. J., Orville-Thomas, W. J., & Yarwood, J.) 475–500 (Springer, 1984).

[115] Hess, B., Bekker, H., Berendsen, H. J. C. & Fraaije, J. G. E. M. LINCS: a linear constraint solver for molecular simulations. *J. Comput. Chem.* **18**, 1463–1472 (1997).

[116] Darden, T., York, D. & Pedersen, L. Particle mesh Ewald: an N⋅log(N) method for Ewald sums in large systems. *J. Chem. Phys.* **98**, 10089–10092 (1993).

[117] Bussi, G., Donadio, D. & Parrinello, M. Canonical sampling through velocity rescaling. *J. Chem. Phys*. **126**, 014101 (2007). 10.1063/1.2408420 17212484

[118] Parrinello, M. & Rahman, A. Polymorphic transitions in single crystals: a new molecular dynamics method. *J. Appl. Phys.* **52**, 7182–7190 (1981).

[119] Best, R. B. & Hummer, G. Optimized molecular dynamics force fields applied to the helix-coil transition of polypeptides. *J. Phys. Chem. B* **113**, 9004–9015 (2009). 19514729 10.1021/jp901540t PMC3115786

[120] Abascal, J. L. F. & Vega, C. A general purpose model for the condensed phases of water: TIP4P/2005. *J. Chem. Phys*. **123**, 234505 (2005). 10.1063/1.2121687 16392929

[121] Xue, Y., Ward, J. M., Yuwen, T., Podkorytov, I. S. & Skrynnikov, N. R. Microsecond time-scale conformational exchange in proteins: using long molecular dynamics trajectory to simulate NMR relaxation dispersion data. *J. Am. Chem. Soc.* **134**, 2555–2562 (2012). 22206299 10.1021/ja206442c

[122] Ban, D. et al. Kinetics of conformational sampling in Ubiquitin. *Angew. Chem. Int. Ed.* **50**, 11437–11440 (2011). 10.1002/anie.201105086 22113802

[123] Khintchine, A. Korrelationstheorie der stationären stochastischen Prozesse. *Math. Ann.* **109**, 604–615 (1934).

[124] Camacho-Zarco, A. R. et al. NMR provides unique insight into the functional dynamics and interactions of intrinsically disordered proteins. *Chem. Rev.* **122**, 9331–9356 (2022). 35446534 10.1021/acs.chemrev.1c01023 PMC9136928

[125] Abril-Pla, O. et al. PyMC: a modern, and comprehensive probabilistic programming framework in Python. *PeerJ Comput. Sci.* **9**, e1516 (2023). 37705656 10.7717/peerj-cs.1516 PMC10495961

[126] Carr, H. Y. & Purcell, E. M. Effects of diffusion on free precession in nuclear magnetic resonance experiments. *Phys. Rev.* **94**, 630–638 (1954).

[127] Koss, H., Rance, M. & Palmer, A. G. Algebraic expressions for Carr-Purcell-Meiboom-Gill relaxation dispersion for N-site chemical exchange. *J. Magn. Reson.* **321**, 106846 (2020). 33128917 10.1016/j.jmr.2020.106846 PMC8129722

[128] Jaynes, E. T. *Probability Theory: The Logic of Science* (Cambridge University Press, 2003).

[129] Priestley, M. B. *Spectral Analysis and Time Series, Two-Volume Set* 1–2 (Academic Press, 1982).

[130] Hoffmann, M. et al. Deeptime: a Python library for machine learning dynamical models from time series data. *Mach. Learn. Sci. Technol.* **3**, 015009 (2022).

[131] Steinhaus, H. Sur la division des corps matériels en parties. *Bull. Pol. Acad. Sci.* **Cl. III 4**, 801–804 (1957).

[132] Espinosa, E., Molins, E. & Lecomte, C. Hydrogen bond strengths revealed by topological analyses of experimentally observed electron densities. *Chem. Phys. Lett.* **285**, 170–173 (1998).

[133] Zhang, J.-Z. et al. The persistence length of semiflexible polymers in lattice Monte Carlo simulations. *Polymers* **11**, 295 (2019). 30960279 10.3390/polym11020295 PMC6419224

[134] Flory, P. J. & Volkenstein, M. Statistical mechanics of chain molecules. *Biopolymers* **8**, 699–700 (1969).

[135] Holla, A. et al. Identifying sequence effects on chain dimensions of disordered proteins by integrating experiments and simulations. *JACS Au* **4**, 4729–4743 (2024). 39735932 10.1021/jacsau.4c00673 PMC11672150

[136] Szöllösi, D. Hierarchical multi-timescale structural dynamics of the disordered N-terminal of p53. https://github.com/dszollosi/p53_TAD_dynamics, 10.5281/zenodo.19704215 (2026). 10.1038/s41467-026-73145-6 PMC13221463 42168213

