# Supplementary material for: Hierarchical multi-timescale structural dynamics of the disordered N-terminal of p53
Source: Nat Commun. 2026 May 21;17:4812. doi: 10.1038/s41467-026-73145-6 (PMC13221463; doi:10.1038/s41467-026-73145-6)
Supplement: Supplementary file 1 — Supplementary Information [file 41467_2026_73145_MOESM1_ESM.pdf]

Supplementary Information for

Hierarchically ordered multi-timescale structural dynamics of the intrinsically disordered p53 transactivation domain

Dániel Szöllősi†, Supriya Pratihar†, Dwaipayan Mukhopadhyay, Ashok Kumar Rout, Mookyoung Han, G. Jithender Reddy, Niklas Ebersberger, Stefan Becker, Gábor Nagy, Sarah Rauscher, Donghan Lee, Reinhard Klement, Christian Griesinger, Helmut Grubmüller

†These authors contributed equally

Corresponding authors:
Christian Griesinger (cigr@mpinat.mpg.de)
Helmut Grubmüller (hgrubmu@mpinat.mpg.de)

Contents

NMR RD profiles 2

$R_{1\rho}$ raw NMR data 6

Comparison of NMR RD profiles at 1.2 GHz versus 950 MHz 7

Comparison of NMR and MD RD profiles 8

Fitting CPMG model to RD profiles calculated from MD simulations 14

Effects of additional residues at the N-terminus 16

Comparison of measured chemical shifts with those calculated from the MD structural ensemble 17

Comparison of different force fields 20

Convergence analysis of the MD simulations 22

Comparison of measured with calculated NMR observables η_{xy}, τ_c, R_1, R_2, and NOEs 25

Further accuracy assessments of the unbiased MD structural ensemble 26

Markov state model analysis: Convergence and helix 2 folding dynamics 32

Comparison of SDFs calculated from MD simulations with NMR measurements 34

Analyses and comparison to polymer models 38

Stretching parameters from RD profiles for the Measles N_{TAIL} peptide 39

p53-TAD tertiary structures resembling protein structure elements 40

NMR RD profiles

Supplementary Figure 1 shows NMR RD profiles measured at 263 K and 1.2 GHz for WT (green dots) and P27A mutant (orange dots) of p53-TAD. The grey lines show fits of the CPMG equation (equations (17) and (18), main text) to these measured data points using Bayesian interference as described in the Methods section, main text (Fitting of 2-state CPMG model to RD profiles measured by NMR at 263 K). The Bayesian posterior was estimated using standard Monte Carlo sampling and provides an estimated uncertainty of the fit. 4000 samples were taken for the posterior distribution of the fitted parameters and used to calculate plausible CPMG profiles. These sample profiles were added to the figure as transparent lines, which appear as a shaded area around the mean fitted curve (darker opaque line). Relaxation times τ were obtained from these fits and are shown in Supplementary Table 1, together with their uncertainties also estimated from the posterior.

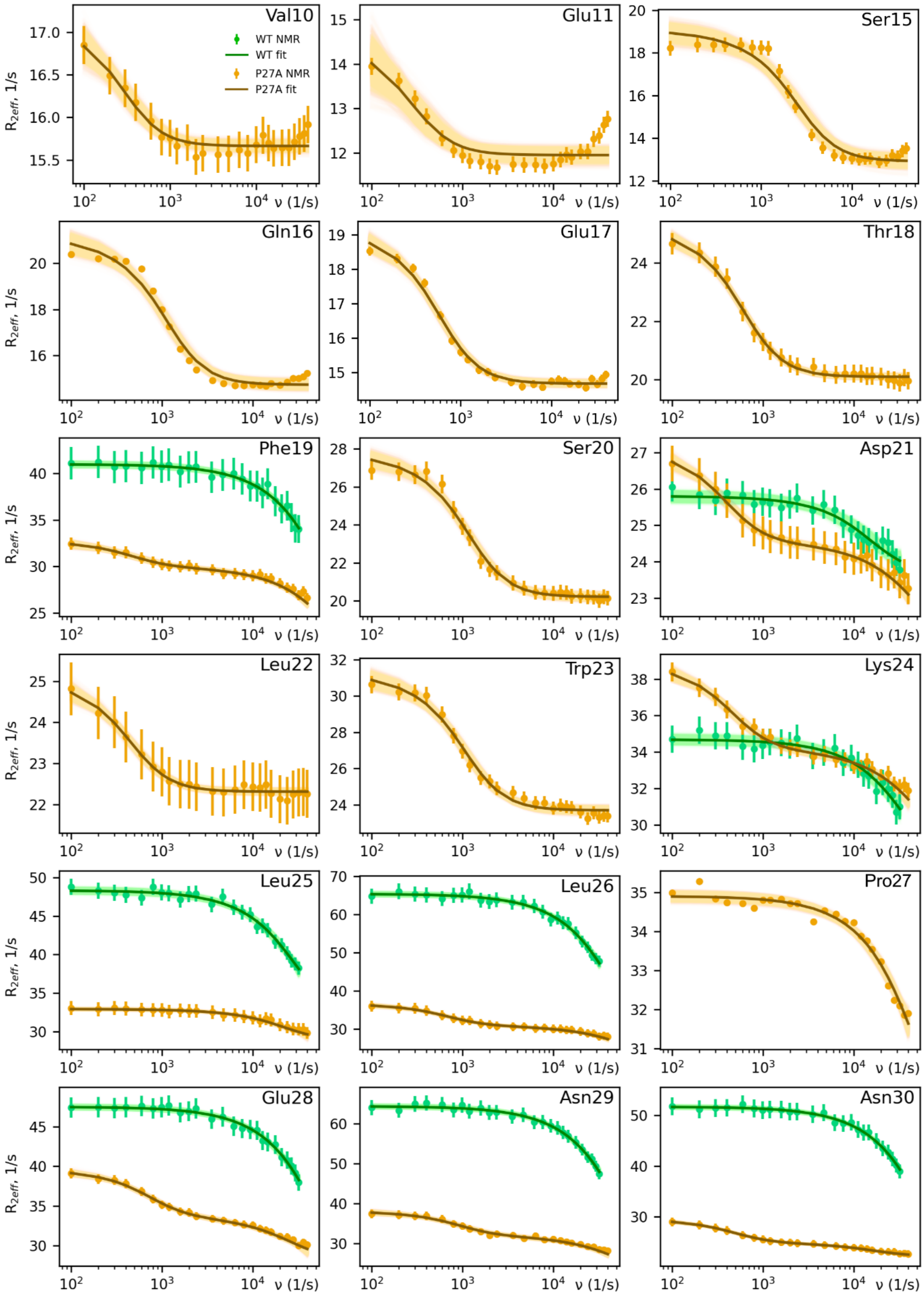
Val10
WT NMR
WT fit
P27A NMR
P27A fit
Glu11
Ser15
Gln16
Glu17
Thr18
Phe19
Ser20
Asp21
Leu22
Trp23
Lys24
Leu25
Leu26
Pro27
Glu28
Asn29
Asn30
R_{2eff}, 1/s
ν (1/s)

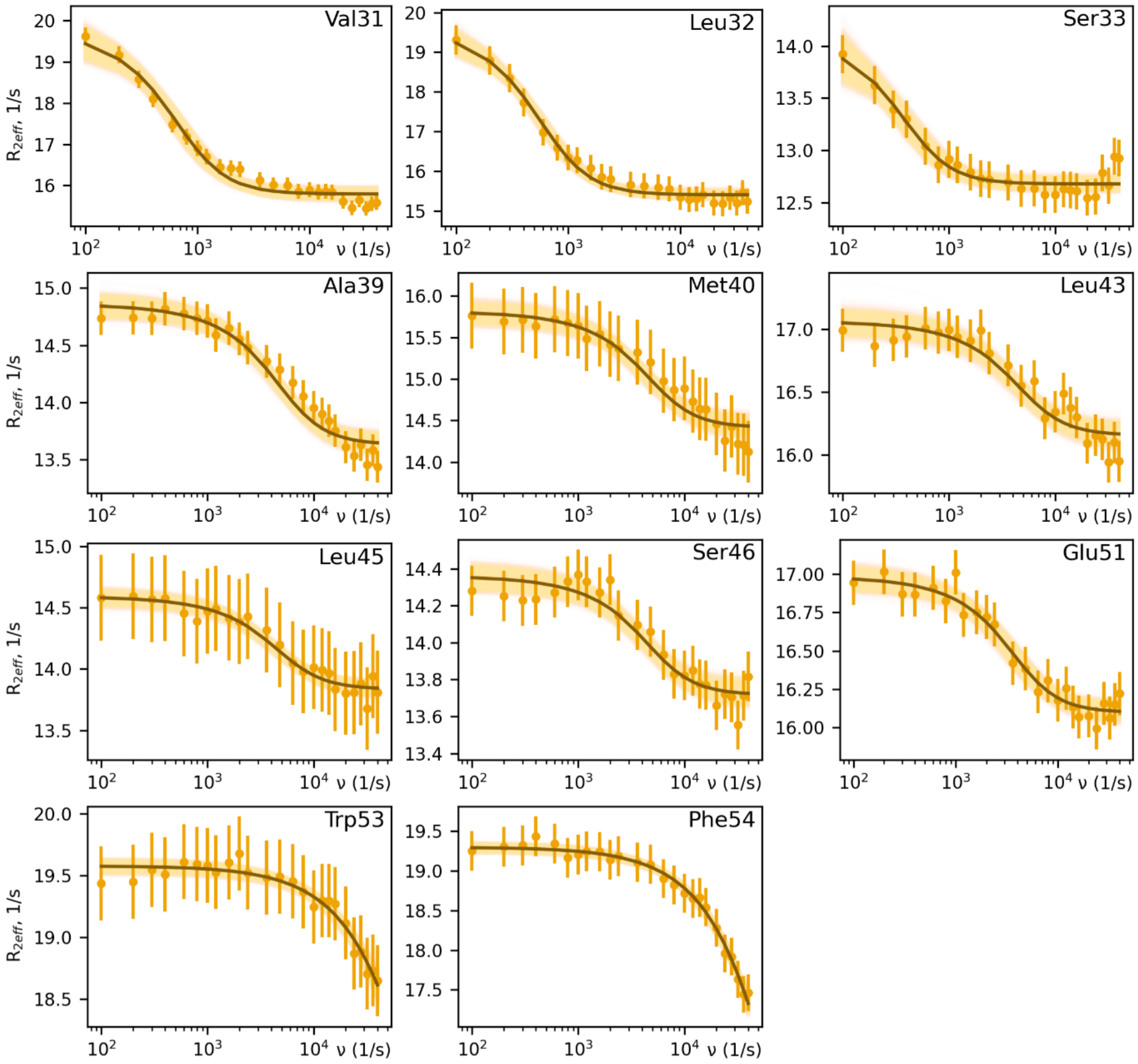

Supplementary Figure 1. Complete set of p53-TAD NMR RD profiles for all fitted residues. Profiles were recorded at 263 K and at 1.2 GHz for WT (green) and for P27A (orange); vertical bars indicate measurement uncertainties (n=2). Superimposed are CPMG fits (equations (17) and (18), main text) to the measured data points (grey lines) and uncertainties of the fits (shaded areas representing the posterior distribution) derived from Bayesian inference (subsection 'Fitting of 2-state CPMG model to RD profiles measured by NMR at 263 K'). Relaxation times τ obtained from these fits are listed in Supplementary Table 1.

Residue	WT τ (µs)	P27A τ (µs)	P27A τ_2 (µs)
Val10		522.01±84.86	
Glu11		553.18±135.67	
Ser15		61.04±6.08	
Gln16		136.37±9.58	
Glu17		259.32±13.21	
Thr18		250.47±10.08	
Phe19	2.51±0.95	330.12±46.89	1.99±1.16
Ser20		130.59±7.47	
Asp21	10.49±2.82	391.28±35.63	2.77±2.41
Leu22		339.25±26.40	
Trp23		144.92±9.39	
Lys24	4.45±2.28	336.20±32.52	2.56±2.22
Leu25	5.53±1.24	7.14±1.19	
Leu26	3.72±0.95	223.13±14.07	2.13±1.23
Ala27		3.28±1.63	
Glu28	2.91±1.11	209.24±13.58	6.16±1.72
Asn29	3.00±0.80	165.59±14.54	2.85±1.67
Asn30	2.61±0.61	317.95±12.83	9.42±0.92
Val31		233.95±25.88	
Leu32		269.43±20.56	
Ser33		404.64±70.14	
Ala39		33.01±1.55	
Met40		33.37±2.04	
Leu43		34.19±2.81	
Leu45		33.98±2.32	
Ser46		34.25±2.59	
Glu51		42.37±5.48	
Trp53		1.98±1.53	
Phe54		2.37±1.56	

Supplementary Table 1. Lifetimes τ obtained from the CPMG fits to the fitted NMR RD profiles shown in Supplementary Figure 1. P27A shows additional, drastically increased relaxation times. For certain residues at helix 1, a second timescale was detected in the same timescale range as for the WT. The amplitude of the faster relaxation process is close to the detection limit and, therefore, was not observed for most residues. Uncertainties indicate the ±SD of the posterior distribution.

$R_{1\rho}$ raw NMR data

Supplementary Figure 2 shows amide backbone ^{1}H off-resonance $R_{1\rho}$ RD experiments which were recorded for WT p53-TAD at supercooled temperatures 262 K, 263 K, 264 K, and 265 K at a 950 MHz ^{1}H field. Timescales used for the Arrhenius extrapolation were obtained using a global leas-squares method by fitting all residues at the same temperature to obtain a single consensus timescale τ using

$$R_{2,\mathrm{eff}}(\omega) = R_{2,0} + \frac{\Phi\tau}{1+\omega^2\tau^2} \ .$$

The resulting timescales τ were 4.76 ± 0.20, 4.15 ± 0.19, 3.76 ± 0.18 and 3.24 ± 0.23 μs at 262 K, 263 K, 264 K, and 265 K, respectively.

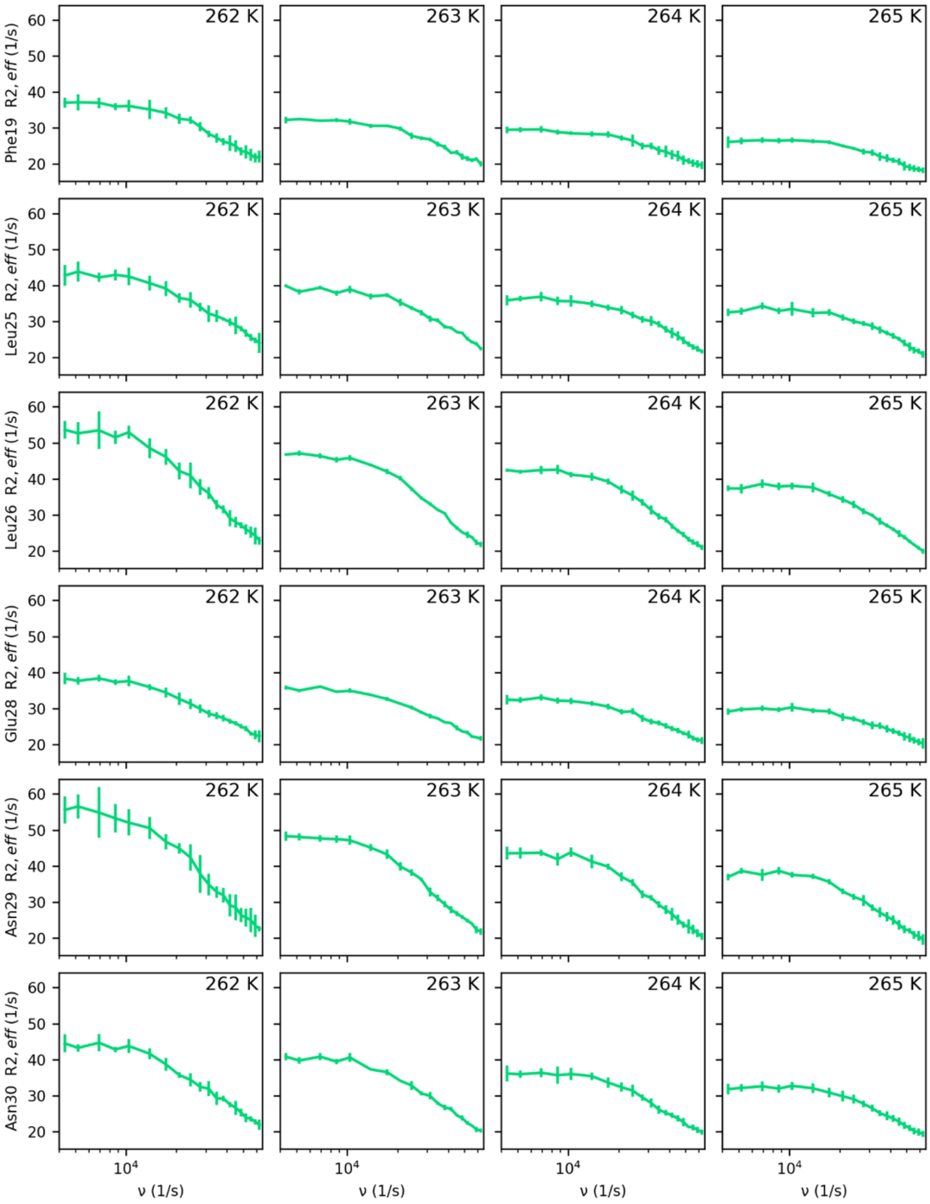

Supplementary Figure 2. $R_{1\rho}$ RD experiments used for the Arrhenius extrapolation. Different helix 1 residues are shown in rows (named on y-axis) measured at different temperatures labelled at the upper right corner. Measurement error is indicated as vertical bars (n=2).

Comparison of NMR RD profiles at 1.2 GHz versus 950 MHz

The detection of the 4 µs motion in the P27A mutant was possible at 1.2 GHz only but not at 950 MHz (Supplementary Figure 3). For such fast motions, the amplitude of the RD profile is very small indeed; however, because it increases quadratically with the field strength, the amplitude is 60% larger at 1.2 GHz compared to 950 MHz, thus enabling detection of these dynamics.

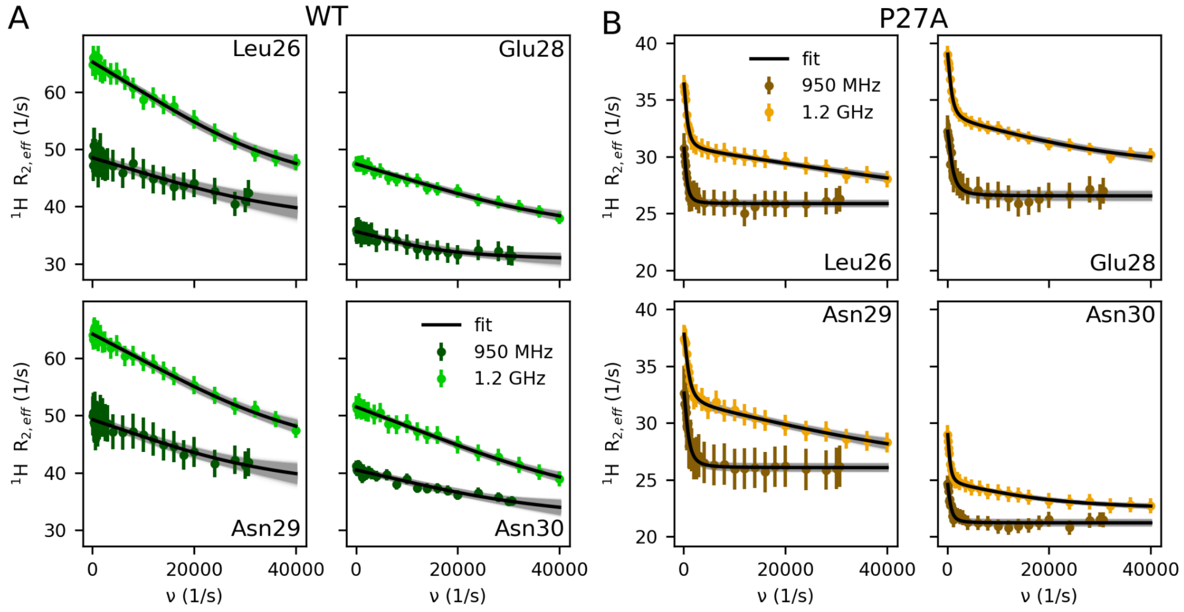

Supplementary Figure 3. Field-dependent detection of fast dynamics in p53-TAD using high-power $^1H^N$ CPMG RD, highlighting the importance of 1.2 GHz B_0 field used in the study. Comparison of RD profiles measured at 263 K and at two magnetic field strengths: 950 MHz (dark colours) and 1.2 GHz (light colours), for both WT (**A**, green/dark green dots with error bars) and P27A (**B**, orange/brown dots with error bars). RD profiles of WT and P27A at 950 MHz were fitted by a single CPMG equation (mean fit: black line, posterior distribution of the mean: shaded grey area); the RD profiles for P27A measured at 1.2 GHz were fitted by the sum of two CPMG functions, yielding two different timescales. Relaxation times τ obtained from these fits are listed in Supplementary Table 2. All experiments were repeated two times (n=2).

**Residue**	**WT τ (µs)**		**P27A τ slow (µs)**		**P27A τ fast (µs)**
	**950 MHz**	**1.2 GHz**	**950 MHz**	**1.2 GHz**	**1.2 GHz**
Leu26	5.02 ± 1.22	4.97 ± 0.64	262.23 ± 24.13	220.37 ± 13.34	3.18 ± 0.76
Glu28	6.31 ± 2.52	4.38 ± 0.73	153.27 ± 18.61	202.48 ± 11.9	4.97 ± 1.29
Asn29	5.62 ± 1.27	4.62 ± 0.64	171.99 ± 14.19	163.37 ± 13.91	3.74 ± 0.96
Asn30	4.64 ± 1.13	3.98 ± 0.52	241.5 ± 36.12	310.69 ± 12.62	8.41 ± 1.05

Supplementary Table 2. Relaxation times (τ) obtained from the fits to the NMR RD profiles. Profiles of WT and P27A were measured at 263 K at 950 MHz and 1.2 GHz, as shown in Supplementary Figure 3. Uncertainties indicate the ±SD of the posterior distribution.

Comparison of NMR and MD RD profiles

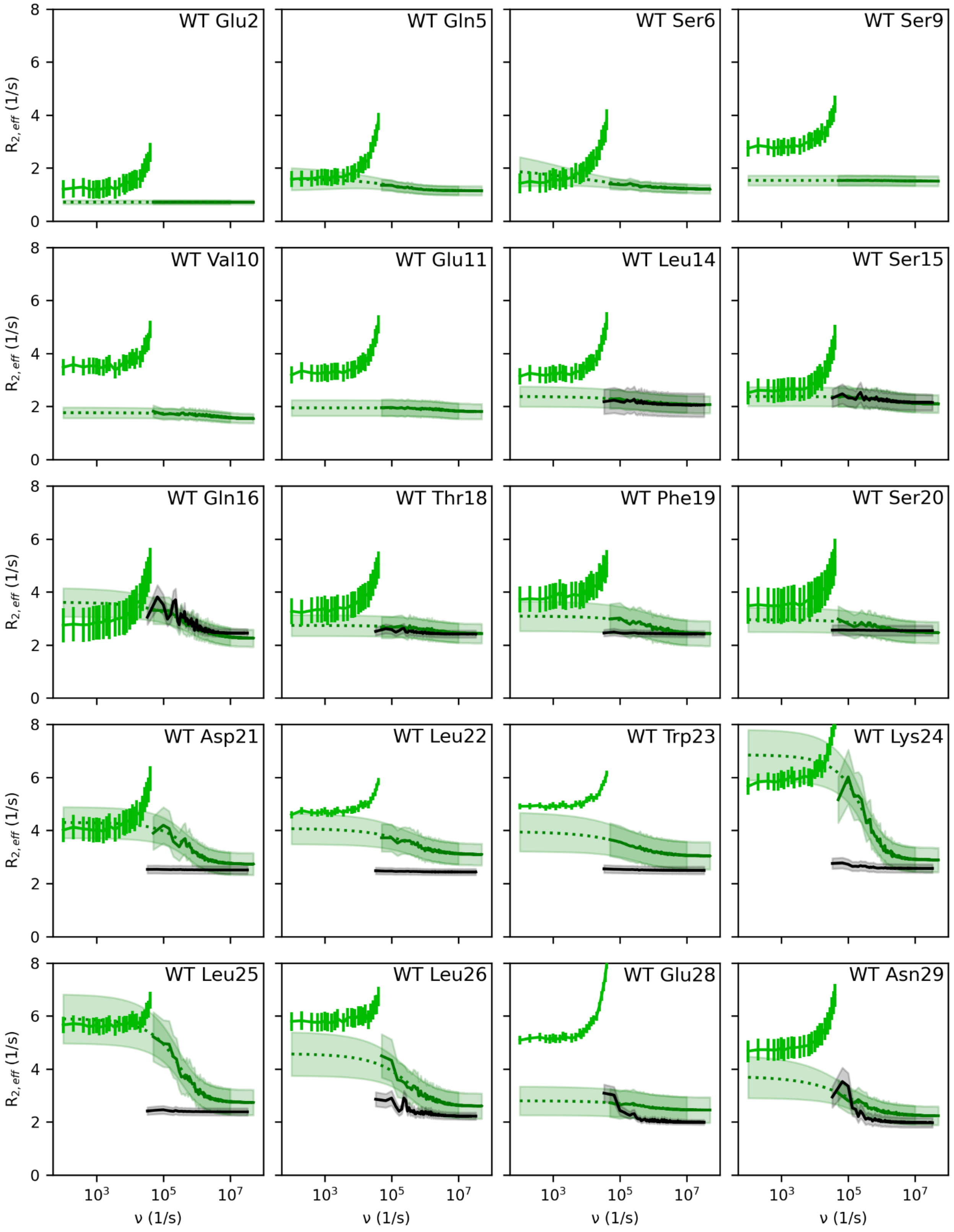

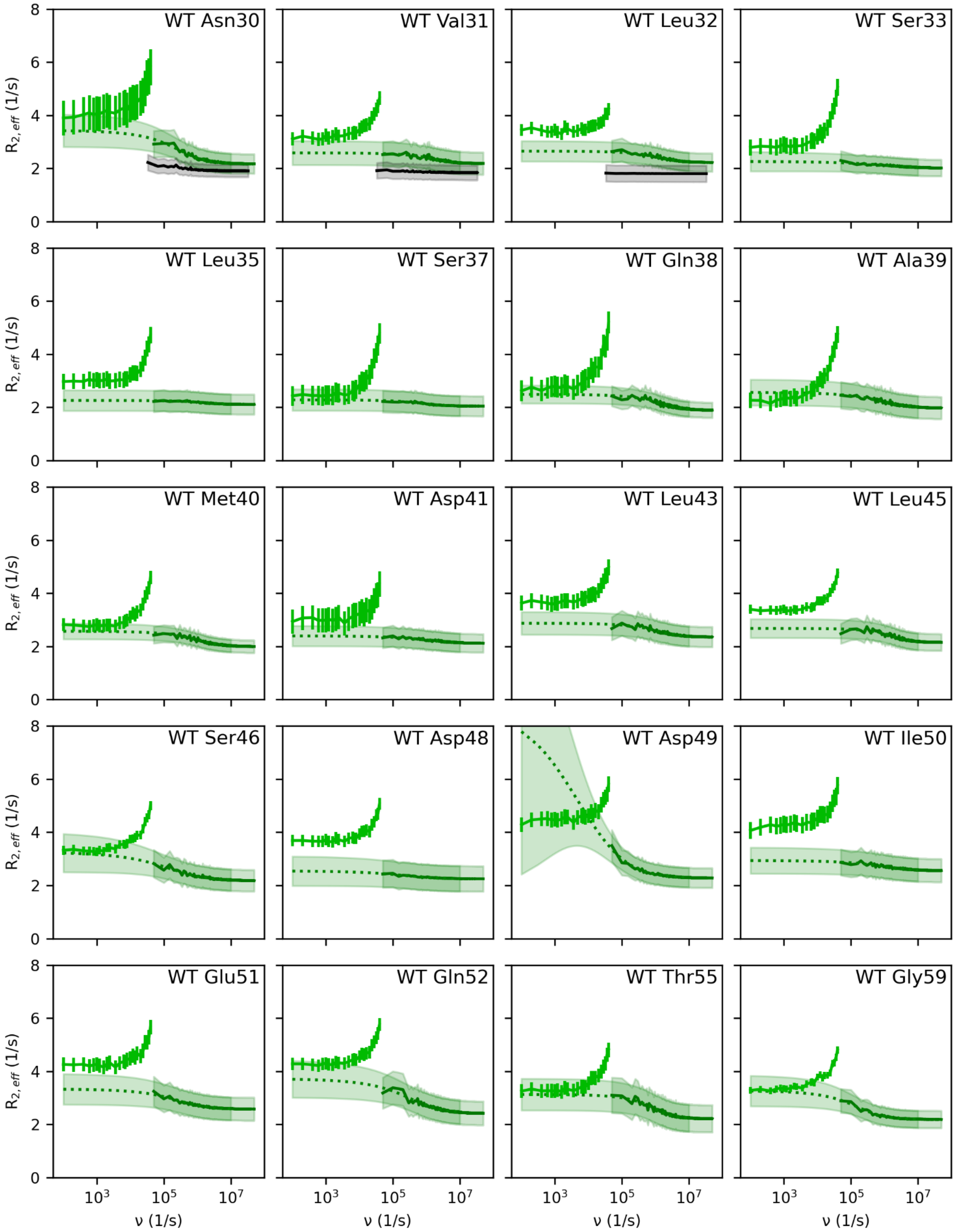
WT Asn30
WT Val31
WT Leu32
WT Ser33
WT Leu35
WT Ser37
WT Gln38
WT Ala39
WT Met40
WT Asp41
WT Leu43
WT Leu45
WT Ser46
WT Asp48
WT Asp49
WT Ile50
WT Glu51
WT Gln52
WT Thr55
WT Gly59
R2, eff (1/s)
ν (1/s)

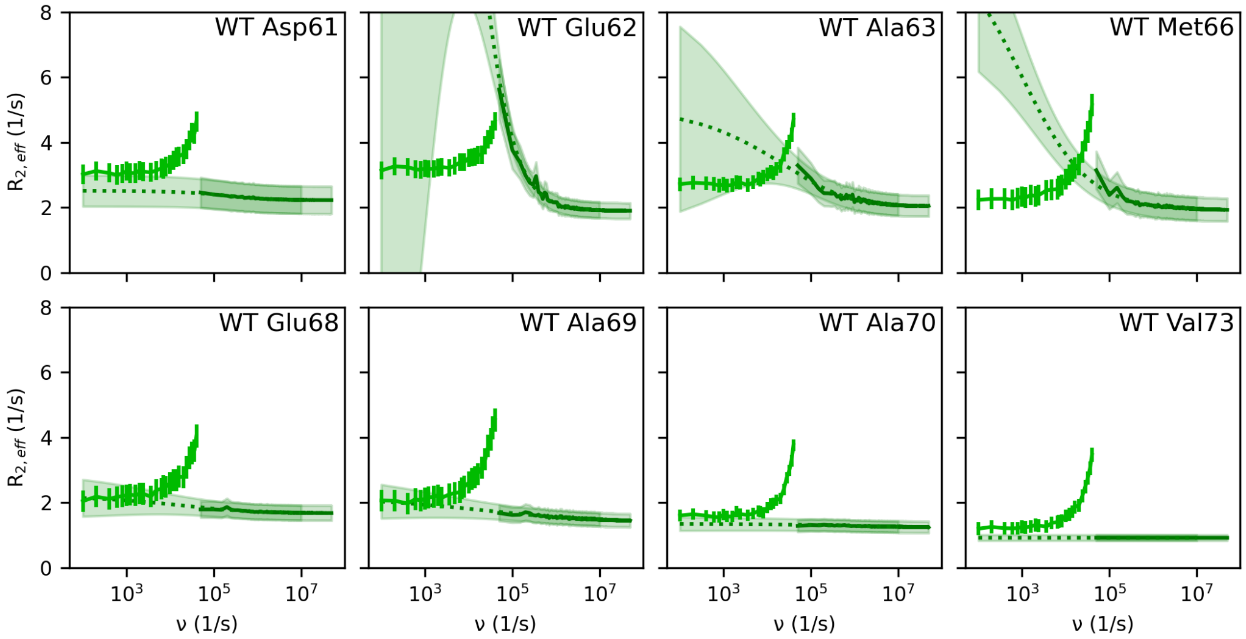

Supplementary Figure 4. Measured vs. calculated p53-TAD WT RD profiles at 298 K. For each residue (panels), profiles derived from NMR measurements are shown in light green (n=2); profiles calculated from MD simulations are shown in dark green (n=30). To facilitate comparison, stretched CPMG-fits to the MD profiles are provided (dotted lines). Errors of the NMR measurements (vertical bars) as well as the statistical uncertainty of the MD profiles (shaded area) were estimated from the standard deviations of repeated measurements or trajectories. The uncertainty of the fits (shaded areas) was estimated from the standard deviations of a posterior sample obtained via Bayes fitting. For residues 14-32, which involve α-helix 1, additional simulations with a fixed ideal α-helix (black lines) were carried out using dihedral restraints, and RD profiles were calculated (black lines and shaded areas, n=20).

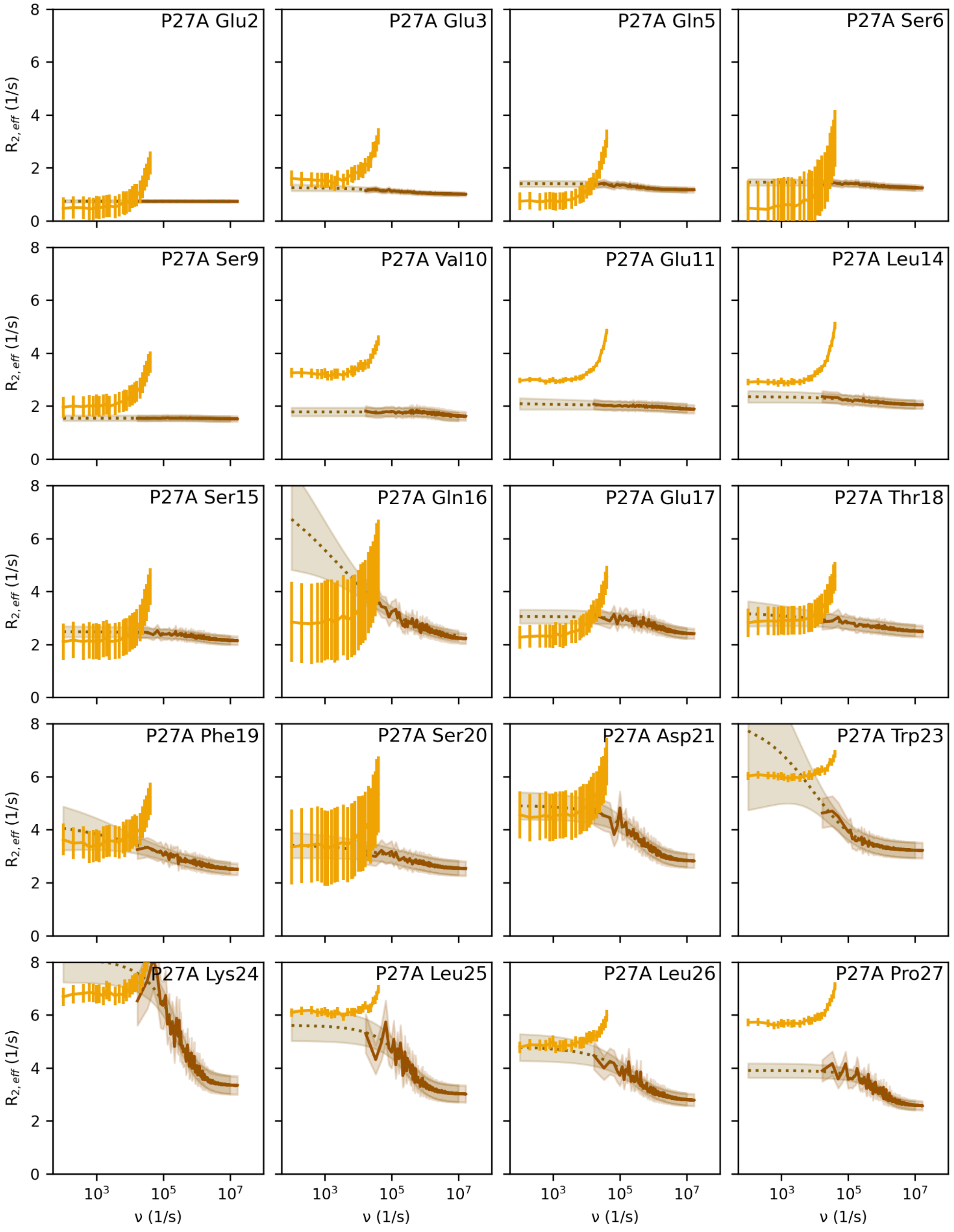
P27A Glu2
P27A Glu3
P27A Gln5
P27A Ser6
P27A Ser9
P27A Val10
P27A Glu11
P27A Leu14
P27A Ser15
P27A Gln16
P27A Glu17
P27A Thr18
P27A Phe19
P27A Ser20
P27A Asp21
P27A Trp23
P27A Lys24
P27A Leu25
P27A Leu26
P27A Pro27
$R_{2,eff}$ (1/s)
ν (1/s)
0
2
4
6
8
10^3
10^5
10^7

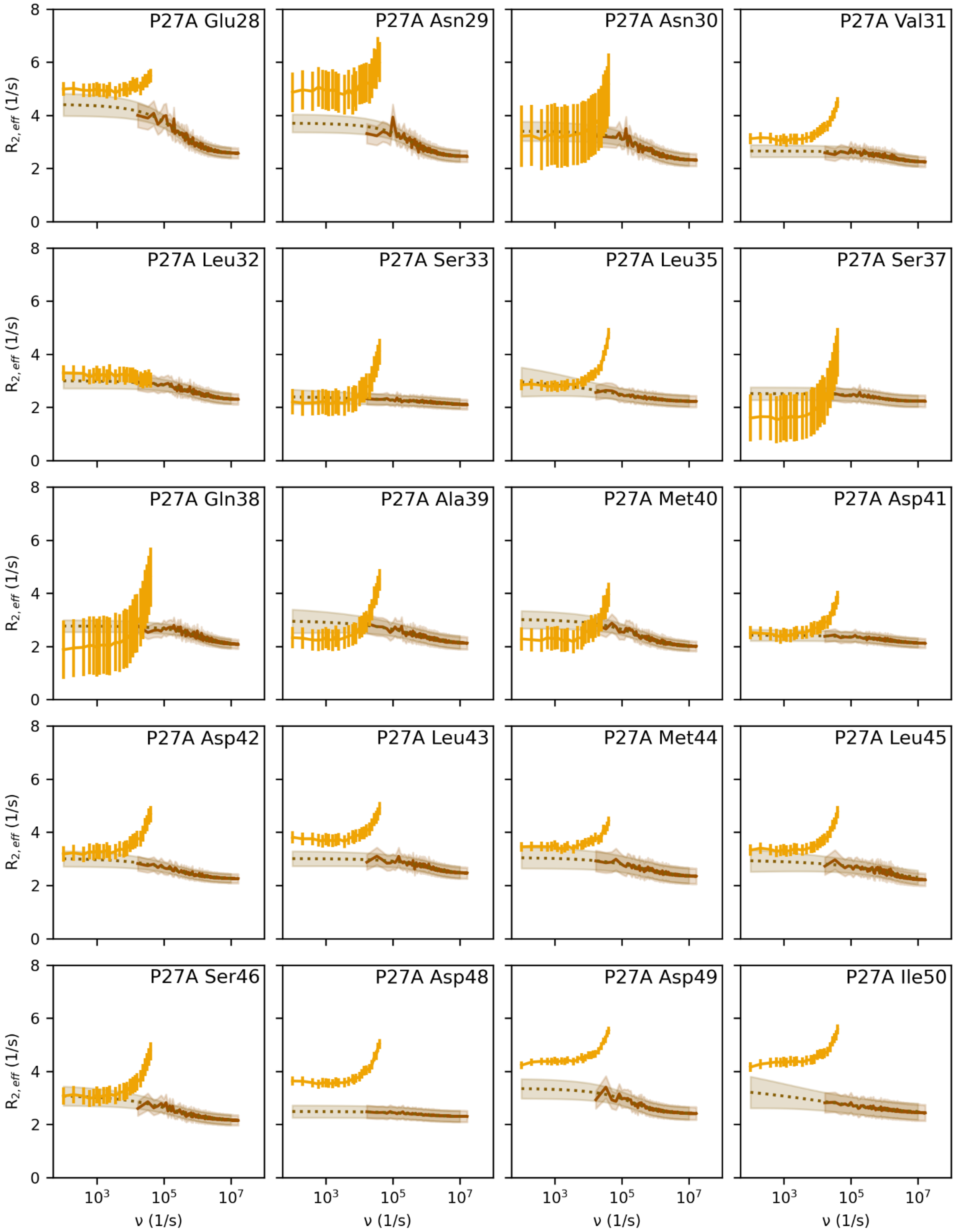
P27A Glu28
P27A Asn29
P27A Asn30
P27A Val31
P27A Leu32
P27A Ser33
P27A Leu35
P27A Ser37
P27A Gln38
P27A Ala39
P27A Met40
P27A Asp41
P27A Asp42
P27A Leu43
P27A Met44
P27A Leu45
P27A Ser46
P27A Asp48
P27A Asp49
P27A Ile50
$R_{2,eff}$ (1/s)
ν (1/s)
0
2
4
6
8
10^3
10^5
10^7

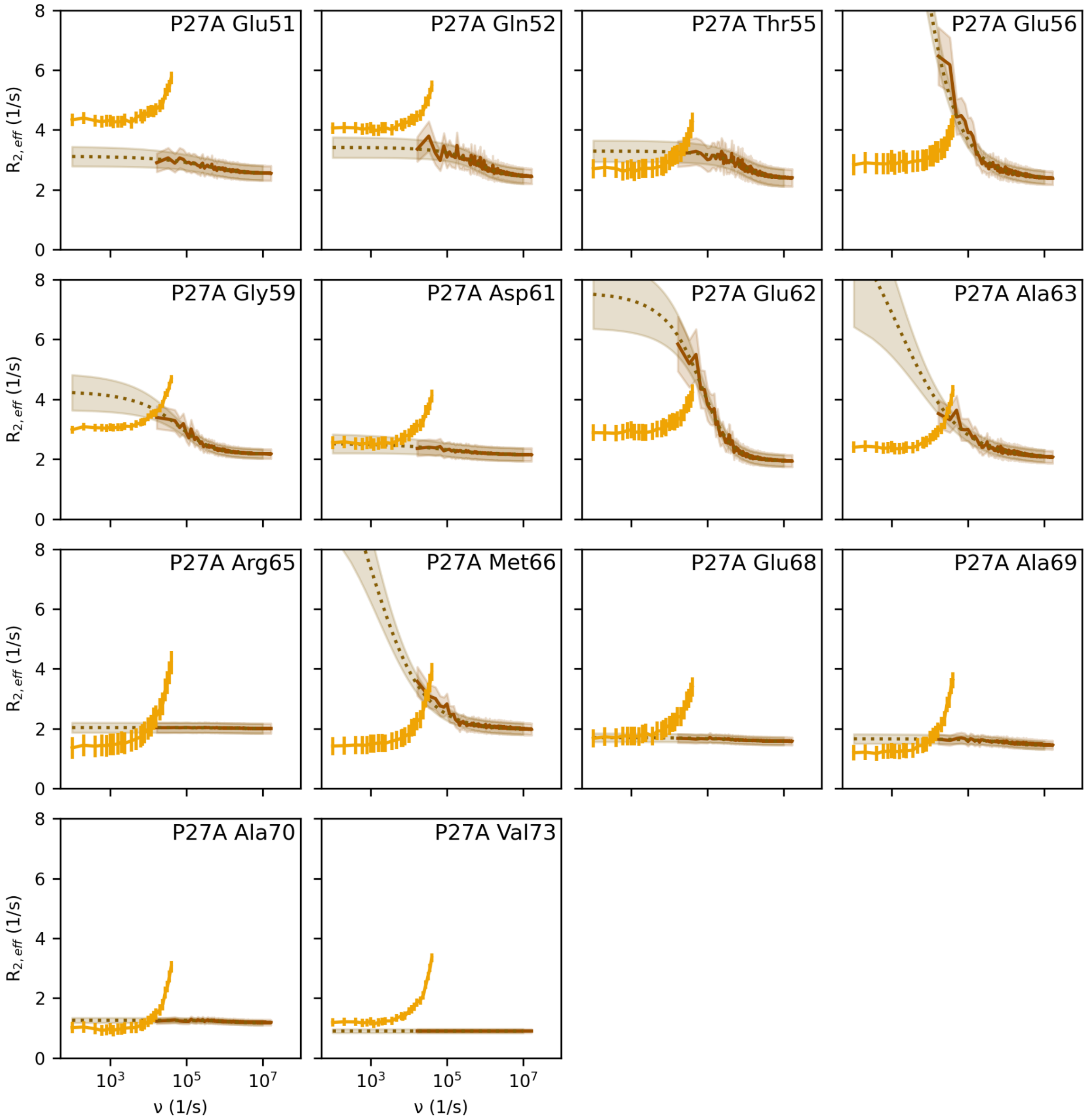

Supplementary Figure 5. Measured vs. calculated p53-TAD P27A mutant RD profiles at 298 K. For each residue (panels), profiles derived from NMR measurements (n=2) are shown in orange; profiles calculated from MD simulations (n=30) are shown in brown. To facilitate comparison, stretched CPMG-fits to the MD profiles are provided (dotted lines). Errors of the NMR measurements (vertical bars) as well as the statistical uncertainty of the MD profiles (shaded area) were estimated from the standard deviations of repeated measurements or trajectories. The uncertainty of the fits (shaded areas) was estimated from the standard deviations of a posterior sample obtained via Bayes fitting.

Fitting CPMG model to RD profiles calculated from MD simulations

As an illustrative example, Supplementary Figure 6 A-D show the RD profile calculated from MD simulations for the Leu25 residue (green) fitted with a non-stretched (A and C, cyan and blue, equation (19), main text) and a stretched (B and D, purple, equation (20), main text) CPMG function, respectively. For the non-stretched CPMG fit, two attempts were made, one with equal weights on all data points of the calculated profile (cyan), and a second one with weights inversely proportional to the frequency (blue). For better visual assessment of the fits both at low and high frequencies, they are shown on a linear (Supplementary Figure 6 A-B) as well as on a logarithmic (Supplementary Figure 6 C-D) scale.

The former fit agrees with the high-frequency part of the profile, but fails for the low-frequency part which contains fewer data points and, hence, carries little weight for the fit. The latter fit, vice versa, approaches the low-frequency part, but fails for the high-frequency part of the calculated profile. In contrast, the stretched function approximates the calculated profile well for the whole frequency range. Supplementary Figure 6E shows the mean stretch parameter γ for all p53-TAD WT residues, as obtained from similar stretched CPMG fits. Missing data points are due to proline residues without backbone amide protons, for which therefore no spectra could be recorded.

Supplementary Figure 6F illustrates a plausible interpretation of why the RD profile is best described by a stretched CPMG function, in terms of conformational dynamics between many conformational states, which are indeed seen in our trajectories. Generalising the well-known analytical results for a two-state Markov process, such multi-state conformational dynamics can be described by a multi-state Markov process[1]. Accordingly, and as has been shown previously[2-4], the RD profile (power spectrum) of such a Markov process with M states is a weighted superposition of M-1 Lorentzian (or here, CPMG) functions, with the M-1 non-zero eigenvalues of the Markov matrix representing the respective relaxation times τ.

As an example, Supplementary Figure 6F shows a superposition of 11 non-stretched CPMG functions (blue to green lines) with 11 relaxation times τ_i (i = 1…11) log-uniformly distributed between 60 ns and 2.5 μs and weights $1/i$. As can be seen, the sum of these 11 CPMG functions (bold green line) is well approximated by a stretched CPMG function (purple) with a typical stretch parameter γ = 0.65, taken from the above fits. Incidentally, this time range overlaps largely with the characteristic times extracted from the Markov model shown in Figure 3A-D (main text).

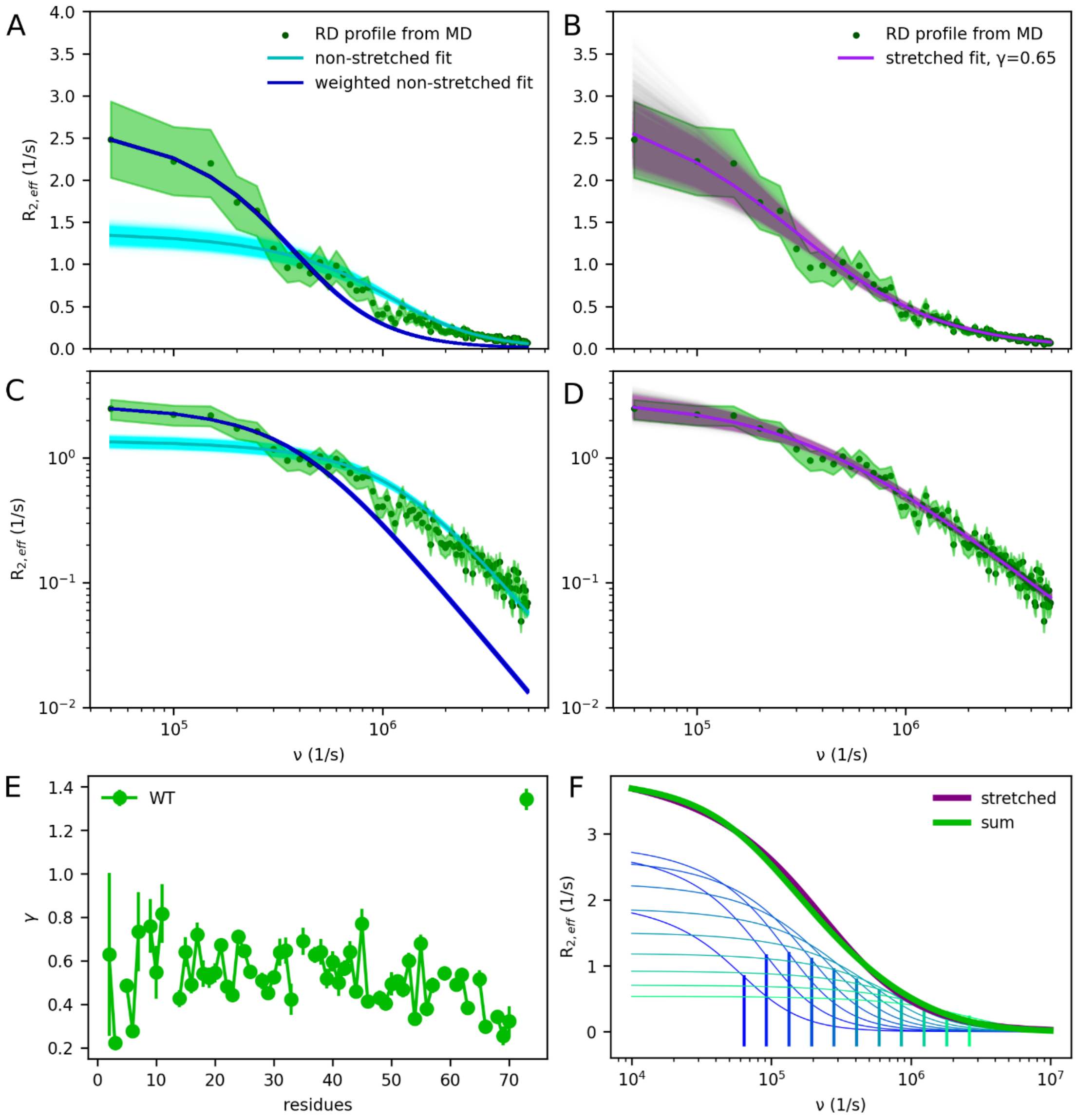

Supplementary Figure 6. Stretched CPMG fits to RD profiles reflect multi-state multi-timescale conformational dynamics. The p53-TAD WT RD profile of the Leu25 residue (green), calculated from 30 MD trajectories, was fitted with a non-stretched CPMG function with equal weights for each data point (solid cyan line) and with weights proportional to $1/\nu$ (solid blue line) (**A**, **C**). The same RD profile was also fitted by a stretched CPMG function (solid purple line) (**B**, **D**). For better visual inspection, profiles and fits are shown on a linear (**A**, **B**) as well as on a logarithmic scale (**C**, **D**). The statistical uncertainty of the calculated profile is shown as a green area around the mean (green dots). Transparent cyan, blue and purple regions show the fitting uncertainty, represented by Bayesian posterior ensembles; the respective best (average) fit is shown as an opaque cyan, blue and purple line. (**E**) Mean stretch parameter γ for all p53-TAD WT residues (n=30), obtained from similar fits to profiles calculated for each residue from our MD simulations; error bars indicate the standard deviation of each parameter. (**F**) A superposition (green line) of 11 non-stretched CPMG functions (blue to green lines, see text) with log-uniformly distributed relaxation times (vertical bars) is well approximated by a stretched CPMG function (purple line).

Effects of additional residues at the N-terminus

Our study aims to understand the hierarchical dynamics of the WT p53-TAD in its native form, and to compare these dynamics to the P27A mutant. Therefore, in our simulations we have constructed and simulated these peptides. In contrast, for technical reasons during the cloning process, the peptides used in the experiments included additional amino acids at the N-terminus, namely a Gly-Ser-extension to the WT sequence and a Gly-Ser-His-Met-extension to the P27A mutant. To assess the effect of these additional residues on the p53-TAD dynamics, we performed additional MD simulations of 5 µs each of the extended P27A construct, for which we expected a larger effect than for the WT. Specifically, five simulations each were performed for the two most likely protonation states of the histidine, singly protonated (neutral) and doubly protonated (charge +1). Simulation parameters and the analysis were as for the non-extended sequences. Due to shorter simulation lengths of the extended constructs, we compared the SDF at the frequency ω_N, which showed faster convergence and for the WT where measurements were available.

As can be seen in Supplementary Figure 7, marked differences between the SDF values calculated from the simulation of the extended peptide and those from the simulations lacking the extension for the N-terminal part of the peptide up to Val10. For the remainder of the peptides, no significant differences are seen. In contrast, SDF values measured by NMR agree very well with those calculated from both simulations of the extended peptide construct, now also for all N-terminal residues. We conclude that the N-terminal deviations reported in the main text are largely due to the presence of these 2-4 additional residues in the measurements. Notably, the SDF values calculated for both possible protonation states are very similar to each other (as well as to the measured NMR values) even close to the histidine at the N-terminus, which suggests that these do not markedly affect the dynamics of the peptide.

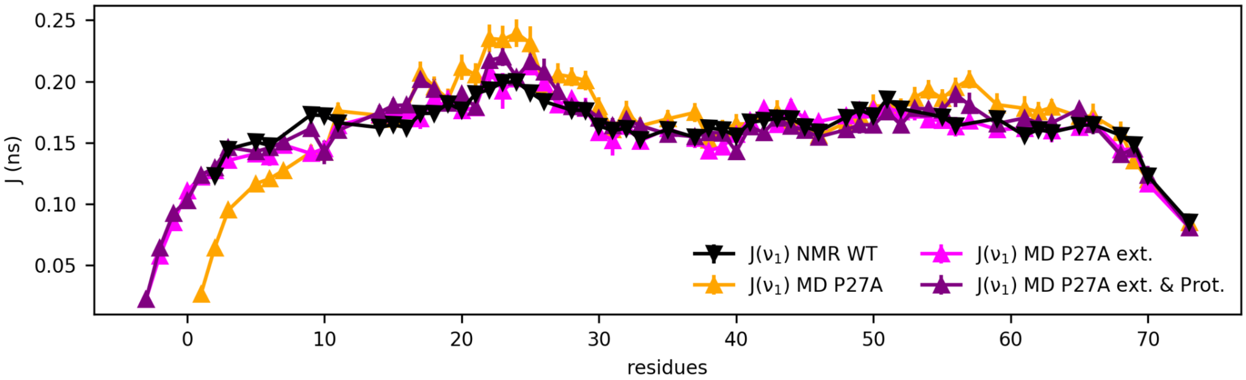

Supplementary Figure 7. Comparison of the extended vs. non-extended p53-TAD construct. Residue numbering is according to the original p53-TAD WT sequence. SDF values calculated at frequency ω_N from our MD simulations of the non-extended p53-TAD P27A (orange, n=30) are compared to those calculated from additional MD simulations of the extended peptide in two different histidine protonation states (single protonated: magenta (n=5) and double protonated: purple (n=5)); for comparison, SDF values measured at 298 K for the WT by NMR (black) are also shown. Errors are indicated as vertical bars and are otherwise smaller than the symbols.

Comparison of measured chemical shifts with those calculated from the MD structural ensemble

Chemical shifts were calculated from our p53-TAD WT trajectories using SPARTA+[5] and, for comparison, SHIFTX2[6]. For a small fraction of the structures, and for unknown reason, chemical shifts below 5 ppm were predicted incorrectly by SHIFTX2, as indicated by a discontinuous chemical shift distribution, which therefore were excluded from the comparison. All further analyses and comparisons with experiments were carried out with chemical shifts calculated by SPARTA+ (Supplementary Figure 8).

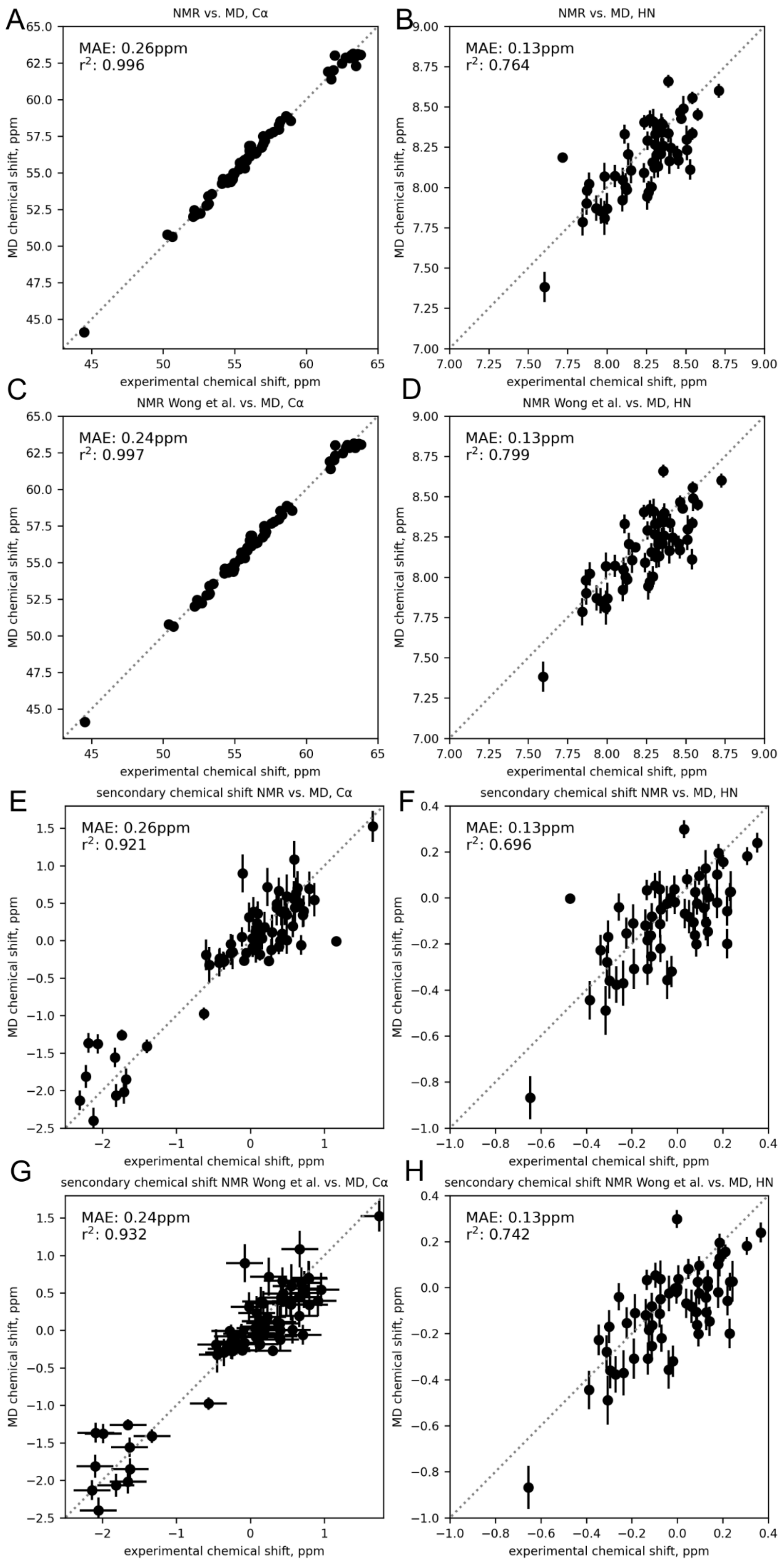

A
NMR vs. MD, Cα
MAE: 0.26ppm
r2: 0.996
B
NMR vs. MD, HN
MAE: 0.13ppm
r2: 0.764
C
NMR Wong et al. vs. MD, Cα
MAE: 0.24ppm
r2: 0.997
D
NMR Wong et al. vs. MD, HN
MAE: 0.13ppm
r2: 0.799
E
sencondary chemical shift NMR vs. MD, Cα
MAE: 0.26ppm
r2: 0.921
F
sencondary chemical shift NMR vs. MD, HN
MAE: 0.13ppm
r2: 0.696
G
sencondary chemical shift NMR Wong et al. vs. MD, Cα
MAE: 0.24ppm
r2: 0.932
H
sencondary chemical shift NMR Wong et al. vs. MD, HN
MAE: 0.13ppm
r2: 0.742
MD chemical shift, ppm
experimental chemical shift, ppm

Supplementary Figure 8. Comparison of predicted with measured p53-TAD chemical shifts. C_α (**A**, **C**) and HN (**B**, **D**) chemical shifts in ppm (circles) derived from MD simulations (n=30) compared to chemical shifts from two independent NMR measurements, our own (**A**, **B**, n=2) and published by Wong et al.[7] (**C**, **D**). The same comparison is also presented using secondary chemical shifts (panels E-H). The random coil values are taken from SPARTA+. Pearson correlation coefficients and mean absolute errors are shown in each panel; a linear fit (dotted line) is shown for comparison. Error bars show, for each nucleus, the standard error of the mean of the chemical shifts calculated for all structures of the MD ensemble. For the C_α chemical shifts, the error bar is smaller than the symbol size in case of the absolute value comparison.

Comparison of different force fields

Although force fields for explicit water MD simulations of IDP have been compared and assessed before[8-10] no consensus has emerged regarding which force field describes the structural ensemble and dynamics of IDPs most accurately. One reason is that the achieved accuracy seems to depend also on the studied IDPs.

We have therefore determined specifically for the p53-TAD IDP the accuracy of combinations of the two force fields that ranked highest in previous assessments with three different water models. In particular, 10 MD simulations covering a total length of 10 µs each were performed using (a) Amber99sbws[11] with the TIP4P2005s[12] water model, (b) CHARMM36m[10] with the default TIP3P water model and (c) CHARMM36m with the OPC water model[13], which recently was found to be similarly accurate for several systems (unpublished data).

For each trajectory and each residue, the SDF was calculated via equations (2) and (3), main text, from which the transverse cross-correlation rate constants η_{xy} and tumbling timescales τ_c were calculated using equations (4-9), main text as described above, as well as rate constants R_1 and R_2 from equations (10-15), main text and NOE values from equation (16), main text. These calculated observables were averaged, errors of the mean were estimated from their standard deviations, and the resulting values for each residue were compared to NMR measurements (Supplementary Figure 9).

As can be seen, for all five observables the best accuracy is achieved by the Amber99sbws + TIP4P2005s force field (green lines), which is generally closest to the measured values and best reproduces the peaks between residues 20 and 26 (helix 1). In contrast, the CHARMM36m + TIP3P (red lines) combination results in too fast dynamics, as reflected by short tumbling times τ_c, while those for CHARMM36m + OPC (purple lines) are too long, indicating too slow dynamics particularly of the highly flexible residues between p53-TAD helix 1 and helix 2.

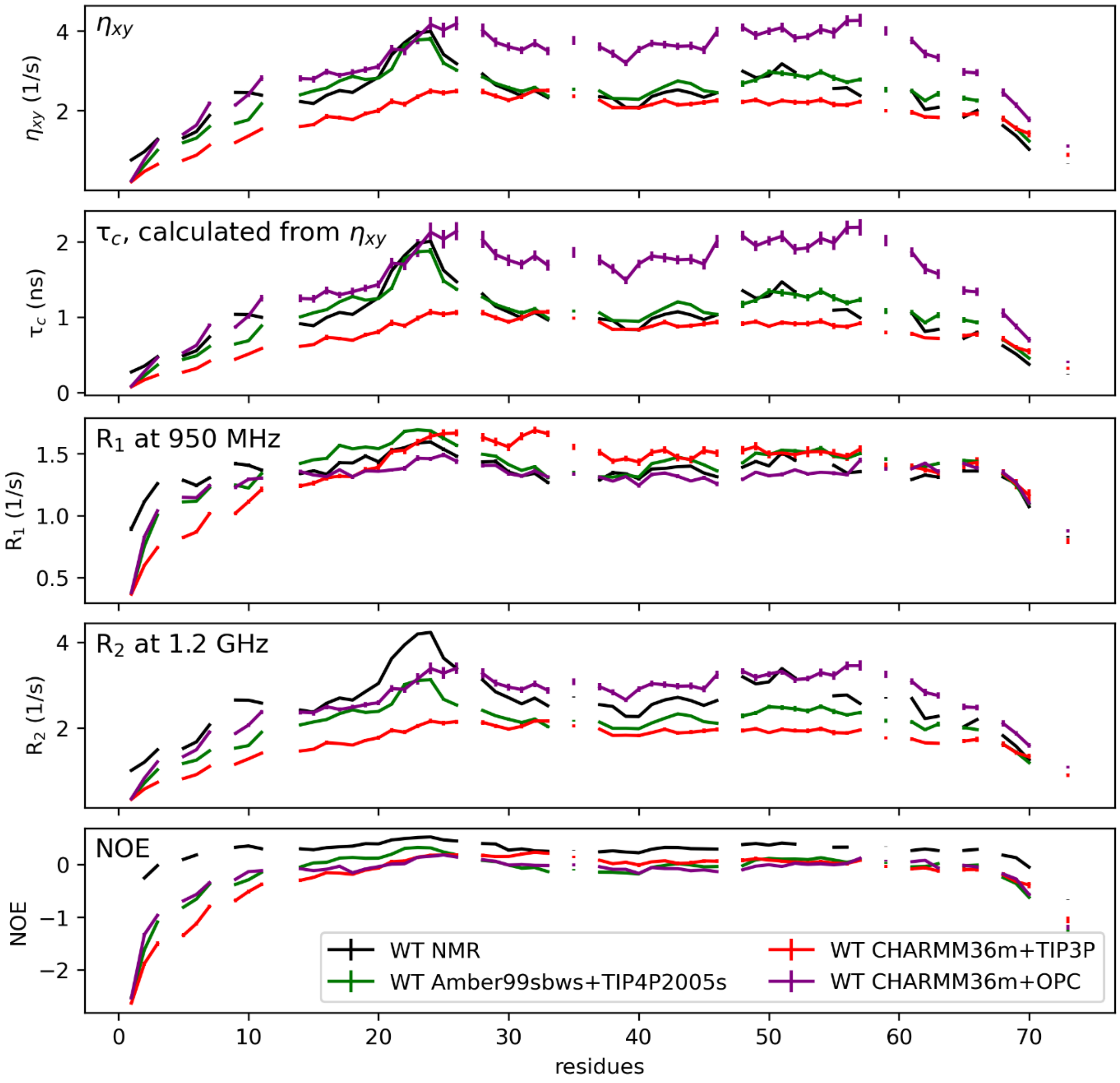

Supplementary Figure 9. Assessment of force field accuracies. The five panels show NMR measurements (black lines, n=2) of (from top to bottom) transverse cross-correlation rate constants η_{xy} and tumbling timescale τ_c (at a magnetic field of 1.2 GHz), rate constants R_1 (at 950 MHz) and R_2 (at 1.2 GHz), and NOE values (at 950 MHz). In each panel and for each residue, these are compared to respective values calculated from MD simulations using the three different force field-water combinations Amber99sbws + TIP4P2005s (green, n=30), CHARMM36m + TIP3P (red, n=20), and CHARMM36m + OPC (purple, n=20). Error bars indicate standard error of the mean estimated from standard deviations, and adjacent residues are connected by lines to guide the eye.

Convergence analysis of the MD simulations

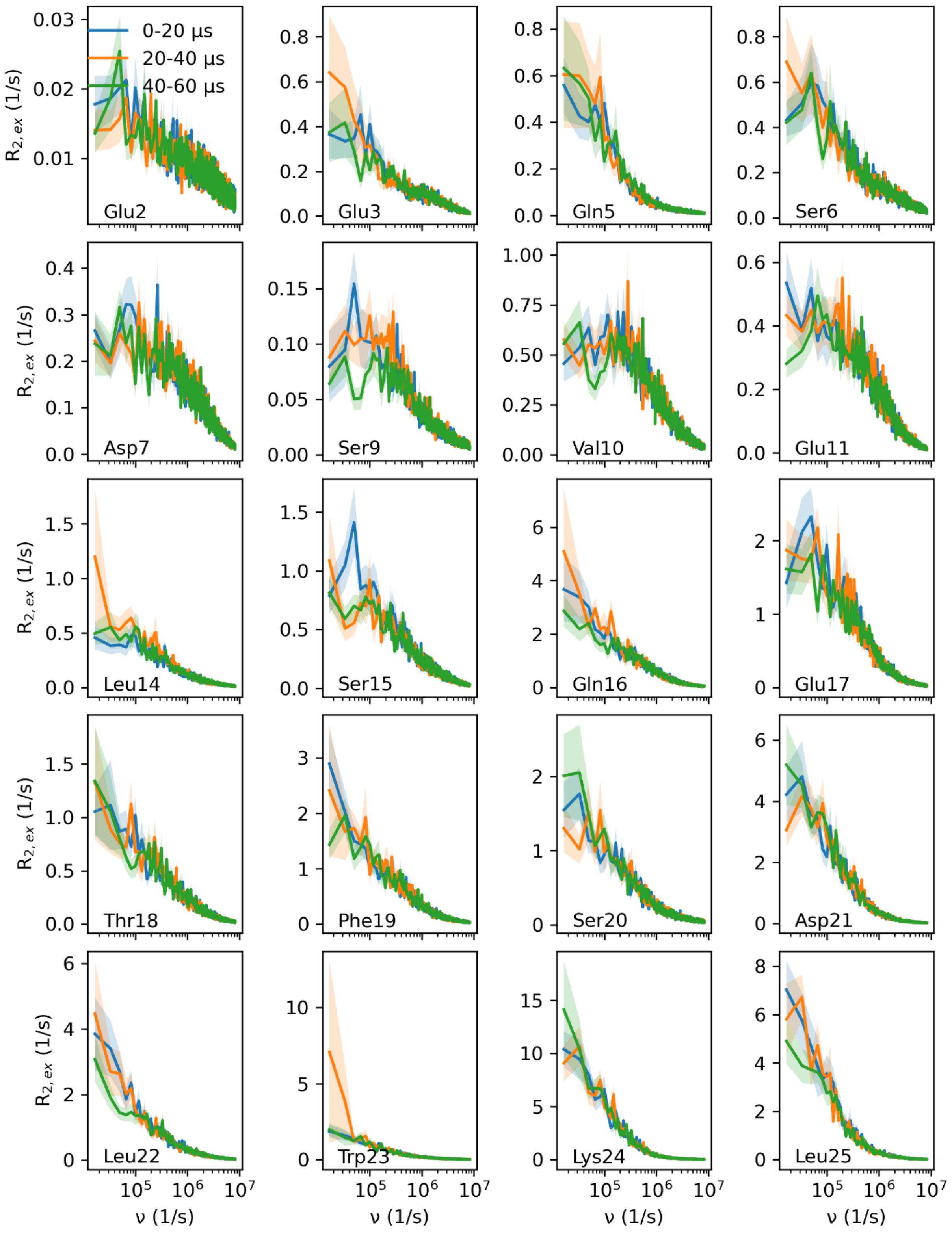

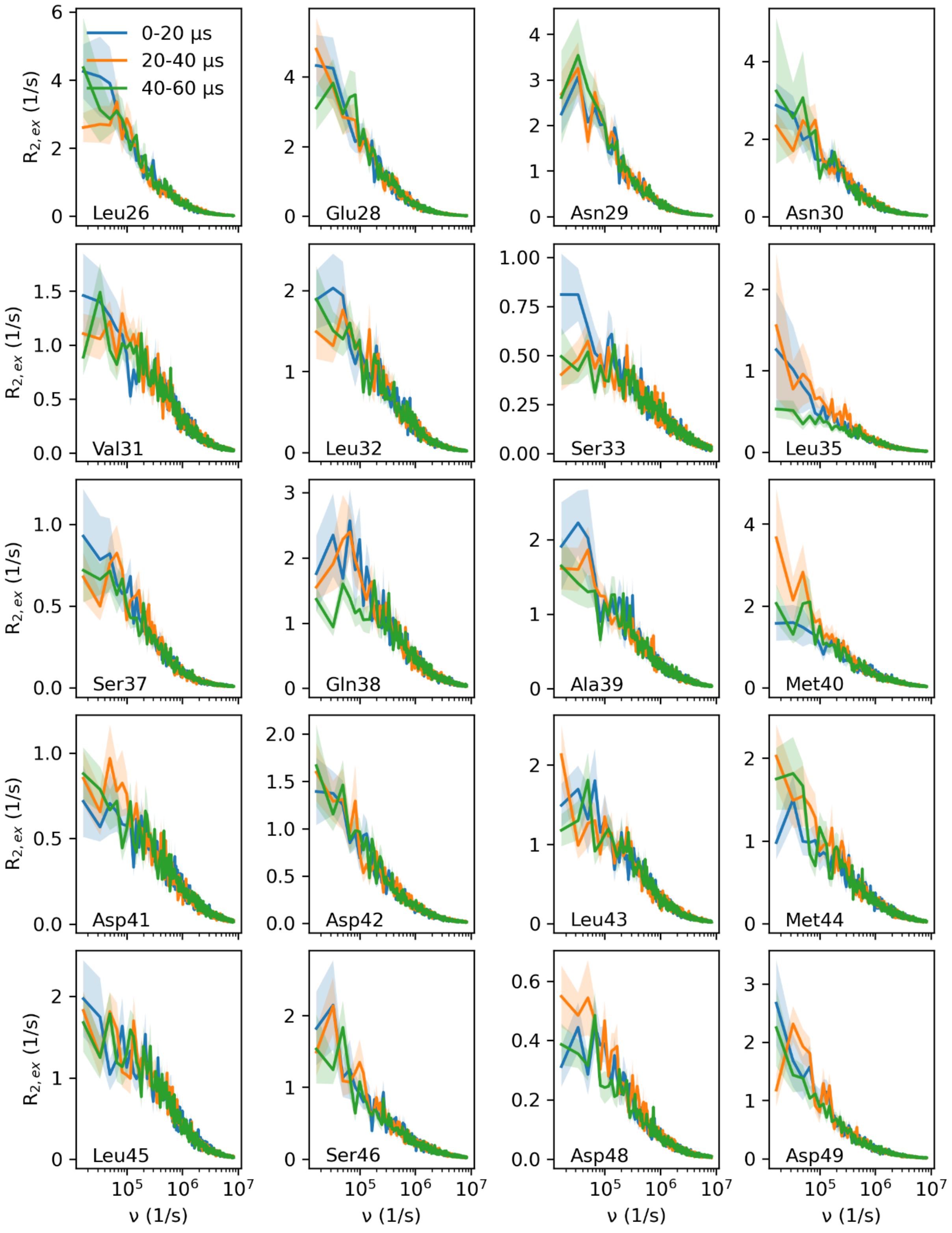
0-20 µs
20-40 µs
40-60 µs
$R_{2,ex}$ (1/s)
ν (1/s)
Leu26
Glu28
Asn29
Asn30
Val31
Leu32
Ser33
Leu35
Ser37
Gln38
Ala39
Met40
Asp41
Asp42
Leu43
Met44
Leu45
Ser46
Asp48
Asp49

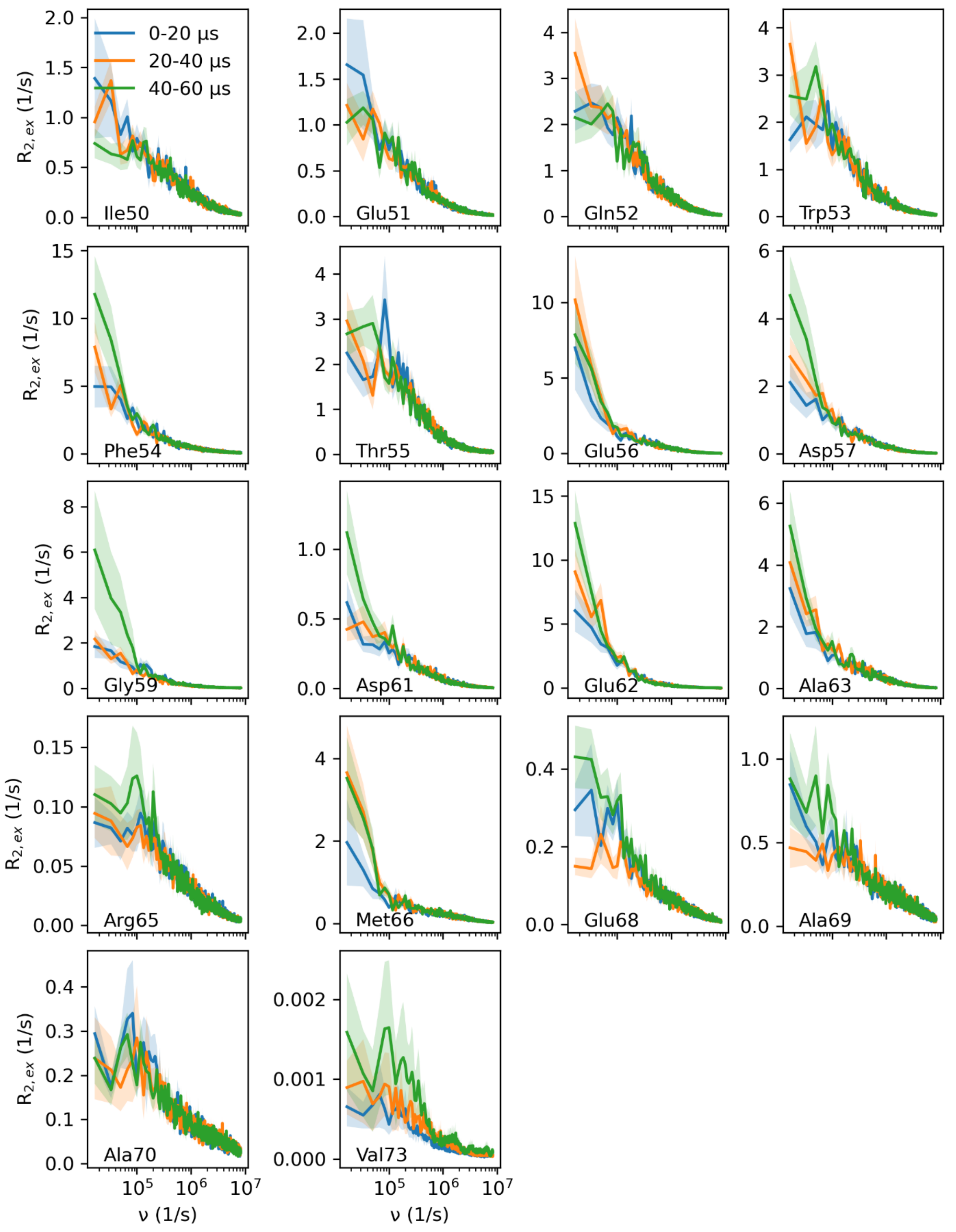

Supplementary Figure 10: MD simulations convergence analysis. Shown are, for each residue, averaged RD profiles (lines) calculated from three non-overlapping 20 μs blocks of the 30 x 60 μs long trajectories of the P27A mutant. Errors of the mean are shown as shaded areas. Note that these profiles only include the exchange related relaxation due to chemical shift changes, which is the critical part affecting convergence; the faster reorientation/tumbling related relaxation are therefore not included.

Comparison of measured with calculated NMR observables η_{xy}, τ_c, R_1, R_2, and NOEs

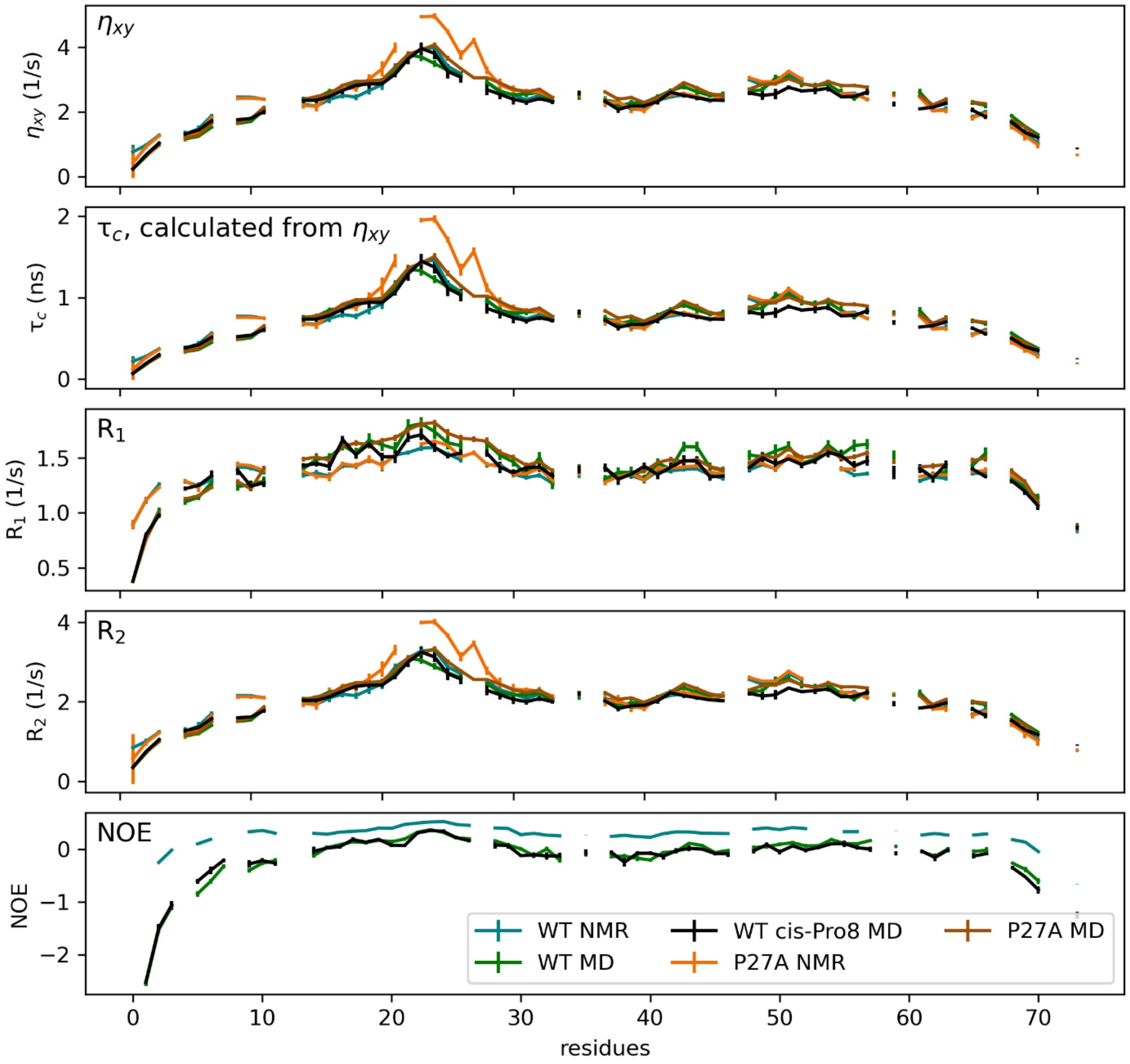

Supplementary Figure 11: Comparison of measured with calculated NMR observables. Comparison of η_{xy}, τ_c, the rate constants R_1, R_2, and NOE values derived from NMR measurements at 298 K (n=2) with values calculated from MD simulations (n=30) for the p53-TAD WT (turquoise and green lines) and the P27A mutant (orange and brown lines). A cis-isomer configuration of Pro8 (black line) shows no significant difference to the trans isomer of the WT (green line). R_1 and NOE values were measured at a magnetic field strength of 950 MHz; η_{xy}, τ_c, and R_2 at 1.2 GHz. The values calculated from our MD simulations used corresponding magnetic field strengths. Lines show the mean of the trajectory-wise calculated observables with error bars (vertical bars). Missing data points are due to proline residues without backbone amide protons, for which therefore no spectra were obtained.

Further accuracy assessments of the unbiased MD structural ensemble

Ensemble averaged radius of gyration and hydrodynamic radius

To further assess the accuracy of the p53-TAD (1-73) ensemble generated from our MD simulations, we calculated ensemble averaged (and distributions of) hydrodynamic radii and radii of gyration and compared these with three independent experiments, namely hydrodynamic radii derived from published SEC and DLS experiments, and radii of gyration derived from SAXS measurements.

First, the hydrodynamic radius R_h was determined from the radius of gyration R_g computed from the obtained structural ensemble by adopting the approach of Nygaard et al. [14],

$$\frac{R_g}{R_h}(N, R_g) = \frac{\alpha_1(R_g - \alpha_2 N^{0.33})}{N^{0.6} - N^{0.33}} + \alpha_3 \quad ,$$

where N = 73 is the number of p53-TAD residues, 0.33 (folded proteins) and 0.6 (disordered proteins) are the fitting parameters as previously published by Nygaard et al., and α_1 = 0.216 Å^{-1}, α_2 = 4.06 Å, and α_3 = 0.821 Å.

Second, R_h was determined from C_α-C_α distances obtained from simulation trajectories by applying the Kirkwood equation[15], corrected for the 19% underestimation of R_h due to the missing hydration shell according to Nygaard et al. [14]

Third, R_h was also determined from MD trajectories by first calculating the diffusion coefficient obtained using the HYDROPRO program[16] with default parameters and a viscosity value of η = 0.9 mPa·s. The diffusion coefficient was converted to R_h using the Stokes-Einstein equation[17].

Supplementary Figure 12A compares R_h distributions estimated from our MD simulations to hydrodynamic radii derived from SEC and DLS experiments (vertical lines). Considering some variation depending on the chosen calculation method, the mean of the MD distribution is larger by ca. 0.15 to 0.3 nm or 7% to 13%. Supplementary Figure 12B provides a more direct comparison of the R_g distribution calculated from our MD simulation ensembles to radii of gyration between 2.4 and 3.0 nm, determined from SAXS measurements at different p53-TAD concentrations[18]. Independent SAXS measurements by Daughdrill et al.[19] yield R_g values of 2.2 nm and 2.8 nm at protein concentrations of 10 and 4 mg/mL, respectively, albeit with a buffer with higher ionic strength (~70 mM vs. ~120 mM). In particular, because our simulation system contains only one isolated monomer and thus best describes a highly diluted solution, the mean radii of 2.98 nm for the WT and 2.94 nm for the P27A calculated from the simulations agree well with the measured R_g values at lower protein concentrations.

Supplementary Figure 12C shows a more direct comparison of calculated vs. measured SAXS spectra. SAXS spectra were computed from our MD ensembles using the program CrySol (part of ATSAS) [20,21] after intensity normalisation. Here, too, the SAXS spectra measured at lower intensities (1.6 and 3.2 mg/mL) agree well with the calculated spectra (χ^2=0. 978 using the 3.2 mg/mL experimental result as reference), which is also the case for the SAXS spectra measured by Daughdrill et al. [19] at 4.0 mg/mL. In contrast, deviations are seen for the spectra measured at higher concentrations of 6.4 mg/mL particularly at low wave numbers, likely due to intermolecular interactions within the sample.

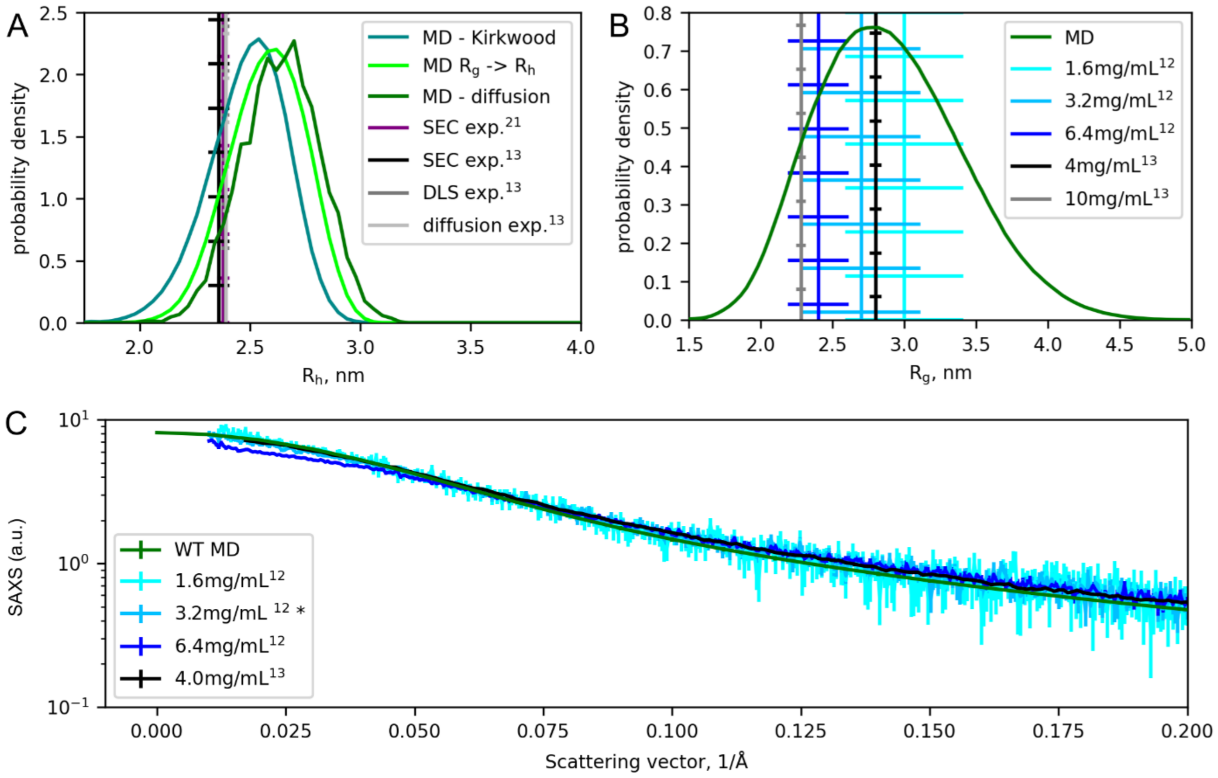

Supplementary Figure 12. Calculated p53-TAD radii and SAXS spectra vs. SEC, DLS, and SAXS measurements. (**A**) Comparison of R_h derived from SEC and DLS experiments (black, shades of blue and grey; means are shown as vertical lines, errors by horizontal lines) with R_h distributions calculated from MD simulations (green, n=30). (**B**) Comparison of R_g calculated from SAXS experiments (means are shown as vertical lines, errors by horizontal lines) at varying protein concentrations with distributions calculated from MD simulations (n=30) for p53-TAD WT (shades of green). (**C**) Direct comparison of measured SAXS spectra (black, shades of blue) with spectra calculated from MD simulations (green, n=30). χ^2=0. 978 using the 3.2 mg/mL experimental result as reference. The superscripts denote the reference from which the value was taken.

FRET distance distributions

Fluorescence Resonance Energy Transfer (FRET) is used to measure intramolecular distances between 2-10 nm within a molecule[22]. In these experiments, two dyes are attached to different positions of interest, and the efficiency of the energy transfer, which depends on the donor-acceptor distance, is measured. For p53-TAD, Huang et al. [23] used single-molecule FRET (SM-FRET) spectroscopy to measure the distance between dyes (donor: Alexa Fluor 488; acceptor: Alexa Fluor 647) attached to cysteine mutated residues 10 and 56 (Supplementary Figure 13A, black line) of a larger p53-TAD construct comprising residues 1-91. Fluorescence from the donor and acceptor was separately detected with two photon-counting modules (SPCM-AQR14; PerkinElmer). The experiments were carried out at 20 °C in phosphate buffer (pH 7, 20 mM phosphate+150 mM NaCl). To prevent sticking of the labelled protein to the cover-glass chamber 1 μM unlabelled protein was added to the solution. The same authors also used time-resolved FRET (TR-FRET) spectroscopy for different, shorter peptide constructs labelled at their N- and C-terminus (with naphthylalanine (Nal-Ala) at the C-terminus as FRET donor and 5-(((acetylamino)ethyl)amino)naphthalene-1-sulfate (EDANS) at the N-terminus as FRET acceptor), specifically, segments with residues 1-17 and 14-30 (Supplementary Figure 13B and C). TR-FRET experiments were carried out with a single photon counting setup with a pulse width of 500 ps at 282 nm. The peptides were measured in 20 mM phosphate+100 mM NaCl, at pH 7 and at 50 μM peptide concentration. Independently, Moses et al. [24] reported FRET efficiencies of a p53-TAD segment comprising residues 1-61, labelled also at the N- and C-terminus (Supplementary Figure 13D) with mTurquoise2 as donor and mNeonGreen as acceptor fluorescent protein in 20 mM phosphate buffer+100 mM NaCl at pH 7.4 and construct concentration of 1 μM. These fluorescent proteins are much larger and potentially interact with the IDP which would bias the measured distance to be smaller; however, no such effect is reported in the original paper. From these FRET efficiencies, we calculated the mean distance between the given residue pair using the Förster equation in the reordered form reported by Moses et al. [24],

$$d(\Delta E_f^{\mathrm{app}}) = \sqrt[6]{\frac{R_0^6}{\Delta E_f^{\mathrm{app}}} - R_0^6} \quad ,$$

where d is the distance, $\Delta E_f^{\mathrm{app}}$ is the measured FRET efficiency and R_0 is the Förster critical distance.

Supplementary Figure 13 compares the mean and uncertainty of these measured distance distributions for the above residue pairs with distance distributions calculated from the respective C_α positions taken from our MD simulations. To facilitate better comparison, all distance distributions were normalised to their maxima. While we compare here a distribution of mean values with a distribution for which full agreement is therefore not expected, the comparison does allow to test the plausibility of the simulated distances even if the sampling of the *in silico* ensemble is different from the real one. For all mean distances, the distances calculated from the simulations agree very well with the measured ones, except for residue pair 1-17 (Supplementary Figure 13B), for which the measured average distance is ca. 2 nm smaller. Here, the missing upstream residues in the sequence of the shorter peptide used for the TR-

FRET experiments might affect the detected distances compared to the full-length p53-TAD protein used for the other measurements. Also, a potential small population of cis-isomer conformations of the four proline residues within this particular sequence, which is not accounted for in the MD simulations, might cause additional shortening of this particular distance. The widths of the respective distance distributions to the measurement error are not expected to agree, nor do they, because those derived from the simulations indicate the ensemble distance distributions, whereas those reported from the FRET measurements involve milliseconds time averages, and are additionally affected by the inevitable shot noise of the recorded FRET efficiencies; for these reasons, the ensemble measurement in Supplementary Figure 13D shows a much narrower distribution than those determined by single-molecule FRET. Finally, Supplementary Figure 13 compares calculated C_α-C_α distances to measured dye-dye distances, for which anisotropic dye-orientation distributions are known to cause deviations. Here, due to the mostly disordered nature of the structural ensemble, we assume dye-orientation anisotropy to be small, and thus also this deviation. Only minor differences are seen between the WT and the mutant P27A. Overall, given these differences between experiment and simulations, the agreement is very good.

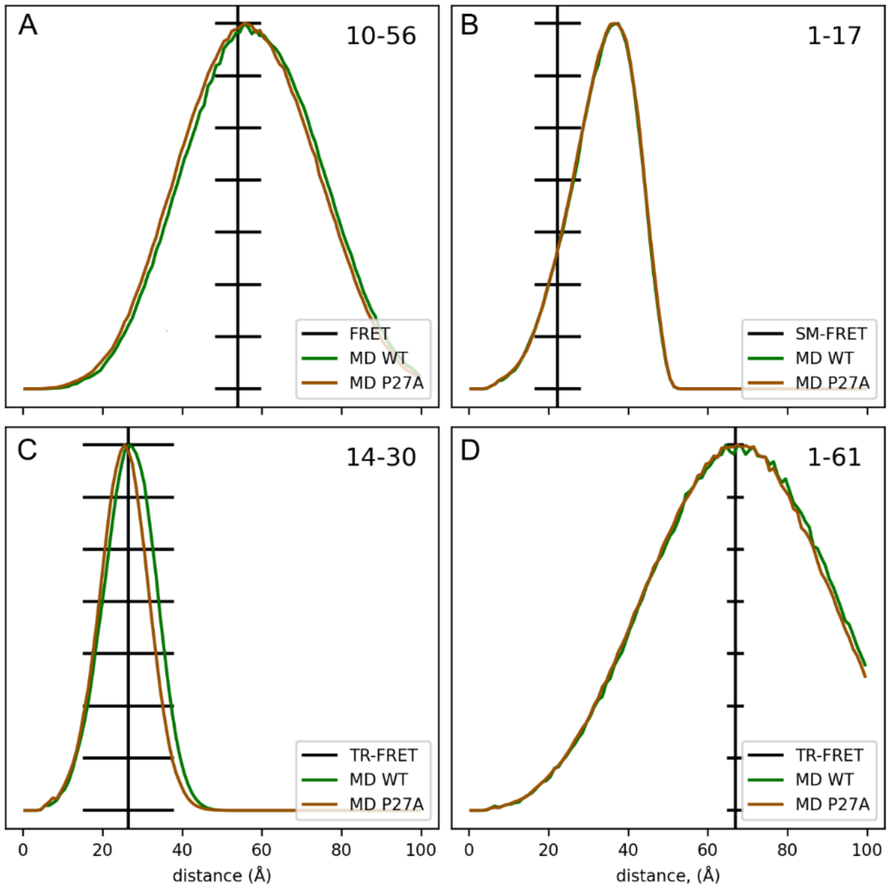

Supplementary Figure 13. Comparison of measured FRET mean distances with distance distributions calculated from our MD ensemble. Fluorophore distance means derived from SM-FRET[23], TR-FRET[23], and FRET[24] experiments (black solid lines, uncertainties shown as horizontal lines) and C_α-C_α distances calculated from MD simulations (n=30) are shown for residue pairs (**A**) 10-56, (**B**) 1-17, (**C**) 14-30, and (**D**) 1-61 for p53-TAD WT (green) and P27A (brown).

Photoinduced Electron Transfer Fluorescence Correlation Spectroscopy (PET-FCS)

Independent experimental information about the dynamics of p53-TAD WT (residues 1-93) was obtained by Lum et al. [25] using Photoinduced Electron Transfer Fluorescence Correlation Spectroscopy (PET-FCS). Briefly, these experiments rest on quenching of a fluorescence dye, attached to a specific residue, via photoinduced electron transfer upon contact with a tryptophane residue at a different position. The authors obtained a total four different constructs, which enabled them to measure the autocorrelation function (ACF) of dye-tryptophane contact formation (e.g. loop closure kinetics) for four p53-TAD segments via the fluorophore-quencher pairs at 13-23, 23-31, 31-53, and 53-60[25].

To compare our atomistic simulations to these measurements, we calculated respective dye/quencher contact formation ACF curves from our MD trajectories, using the C_α distances between the same residue pairs as in the experiments. Having been unable to obtain the original data by Lum et al., we recalculated the measured autocorrelation functions from the published loop closure coefficients and parameters obtained from the published figures. Because the absolute scaling of the ACF is unrelated to the timescales of interest, its amplitude was manually scaled to match the calculated ACF. As can be seen in Supplementary Figure 14, the ACF calculated from the simulations (green) agrees well with the measured ACF (black) for all four dye/quencher pairs and for nearly all lag times longer than 100 ns. For shorter lag times, deviations are seen, which however remain within the range of the experimental fluctuations seen in Fig. 1 by Lum et al.

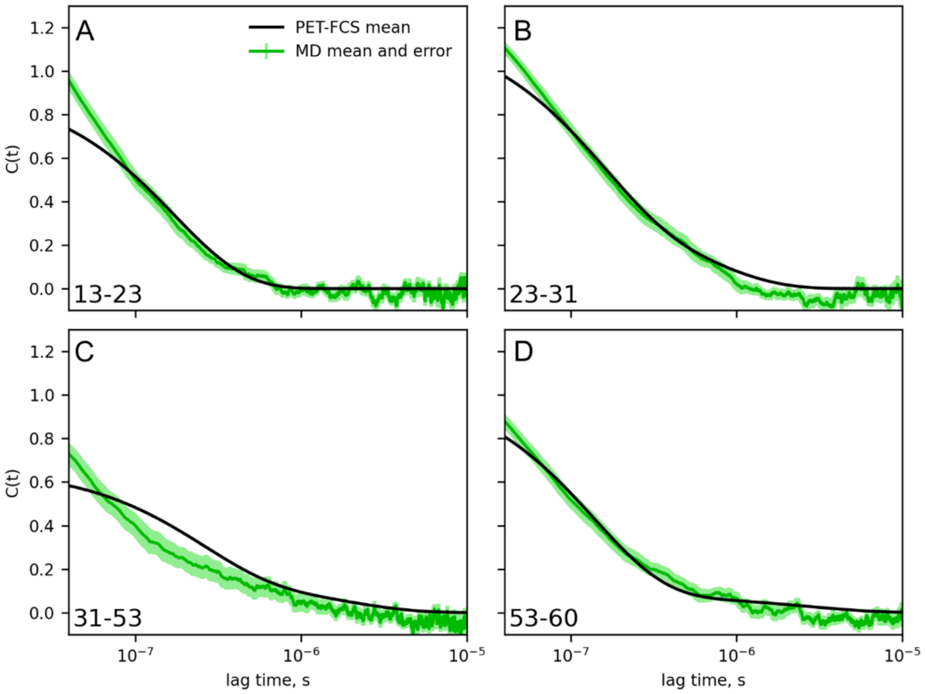

Supplementary Figure 14. Comparison of Photoinduced Electron Transfer Fluorescence Correlation Spectroscopy (PET-FCS) measurements with our atomistic simulations. Shown are fluorescence autocorrelation functions reproduced from published experiments (black), and fluorescence autocorrelation functions calculated from our MD trajectories (green, n=30) with error estimates (light green shared areas) for dye/quencher pairs at residues (**A**)13-23, (**B**) 23-31, (**C**) 31-53, and (**D**) 53-60.

Paramagnetic Relaxation Enhancement (PRE)

Residue distances also were derived from Paramagnetic Relaxation Enhancement (PRE) experiments[26], in which the unpaired electron spin of a paramagnetic spin label provides long-range distance information by enhancing NMR relaxation rates of nearby nuclei. Lowry et al.[27] introduced cysteine residues at four different positions of a p53-TAD (residues 1-73) construct, namely D7C, E28C, A39C and D61C, and attached the paramagnetic spin label MTSL to all four mutants. ^{15}N heteronuclear single quantum coherence (HSQC) spectra were recorded, from which resonance intensity ratios I_{ox}/I_{red} were calculated[27] (Supplementary Figure 15, black dots). Ratios I_{ox}/I_{red} for these four residues (green lines) were also calculated from the C_α-C_α distances taken from our MD ensemble as described by Liu et al. [28] and using the reported proton linewidth R_2 and correlation times τ_c[27].

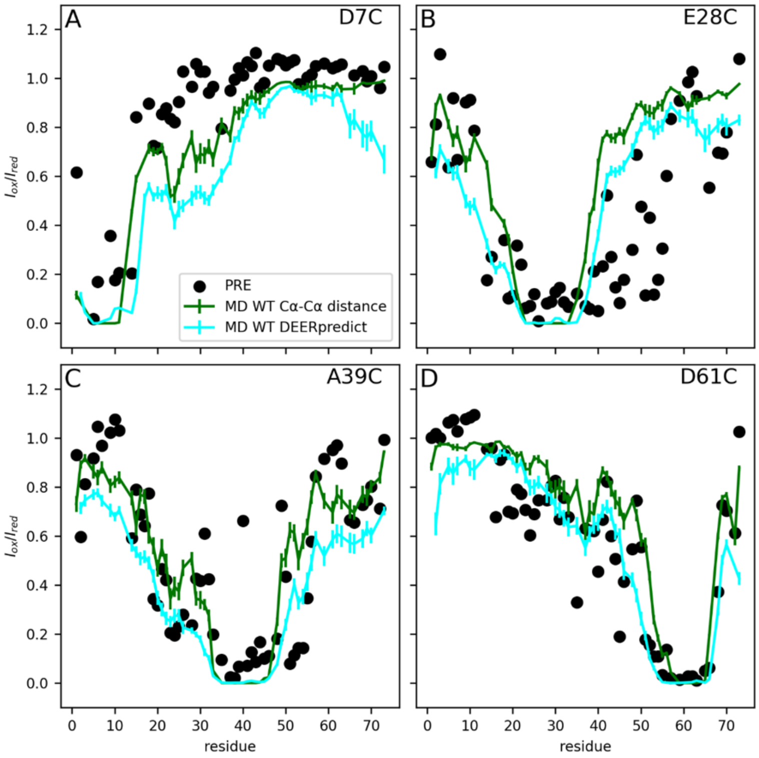

Supplementary Figure 15. Comparison of measured PRE intensity ratios with those calculated from MD ensemble. Resonance intensity ratios I_{ox}/I_{red} determined from PRE experiments[27] (black dots) and intensity ratios calculated from C_α-C_α distances obtained from MD simulation trajectories (green lines) and using the prediction tool DEER-PREdict[29] for label positions (**A**) D7C, (**B**) E28C, (**C**) A39C, and (**D**) D61C. Error bars indicate errors of the mean over 30 trajectories.

Overall, the calculated ratios agree rather well with the measured ones, with a root mean squared deviation (RMSD) of 0.19, 0.33, 0.21, and 0.17 for D7C, E28C, A39C, and D61C, respectively. These RMSD values are similar or smaller than those reported by Liu et al. [28]. The only notable discrepancy is seen for E28C between residues 35 and 55, indicating somewhat shorter distances to E28C in the experiment. This finding is in line with the above comparisons of molecular size (R_h and R_g), which also point to a slightly more extended MD ensemble. As a control, we have also calculated the PRE curves using DEER-PREdict[29] (cyan curves), which also reflect the overall shape of the measured curves, albeit with somewhat larger RMSD values of 0.29, 0.28, 0.23 and 0.17, respectively.

Markov state model analysis: Convergence and helix 2 folding dynamics

The Markov model for p53-TAD WT helix 2 comprising residues 40-53 (Supplementary Figure 16) was derived from the same MD ensemble in a similar way as described for helix 1 above, except that here five initial macro-states were requested, and after inspection as described above, no further merger was deemed necessary.

For both Markov models of helix 1 and helix 2, and for each final Markov state, a representative structure was determined at the highest density (most likely) region in the space spanned by the first two TICA components. These representative structures are depicted in Figure 3C, main text (helix 1) and Supplementary Figure 16 (helix 2). As can be seen from the estimated free energies of the individual states in Supplementary Figure 16C, helix 2 is much less stable than helix 1 (Figure 3C, main text) and also exhibits more diverse folding dynamic.

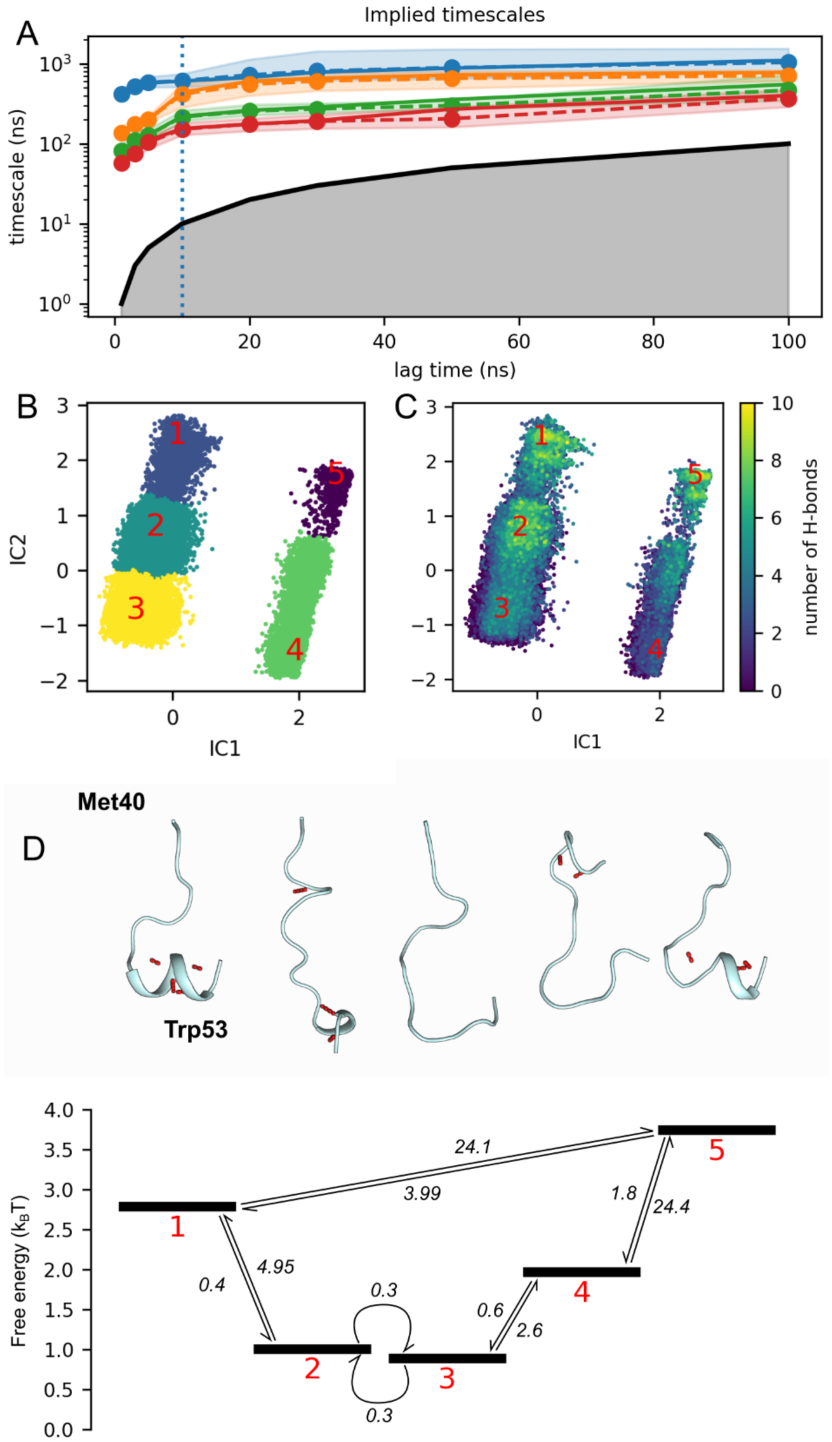

Supplementary Figure 16. Implied timescale analysis of the Markov state model of helix 1 and the multi-timescale folding/unfolding dynamics of helix 2. (**A**) Implied timescales of the Markov state model of helix 1 with lag times from 1 to 100 ns. The model with lag time of 10 ns was used in further analysis and is indicated by a dashed vertical line. (**B**) Projection of the conformational ensemble of helix 2 (residues 40-53) onto the TICA space defined by the two collective coordinates IC 1 and IC 2 that contribute most to the intra-helical dynamics of the WT trajectories. Colours and red numbers indicate five Markov states. (**C**) The same projection as in (B), but coloured by the number of intra-helical hydrogen bonds. (**D**) Representative structures of the five Markov states (intra-helical hydrogen bonds shown in red dots, top) and their free energies (black bars, below); mean first passage times of transitions between the states (arrows) are shown in µs.

Comparison of SDFs calculated from MD simulations with NMR measurements

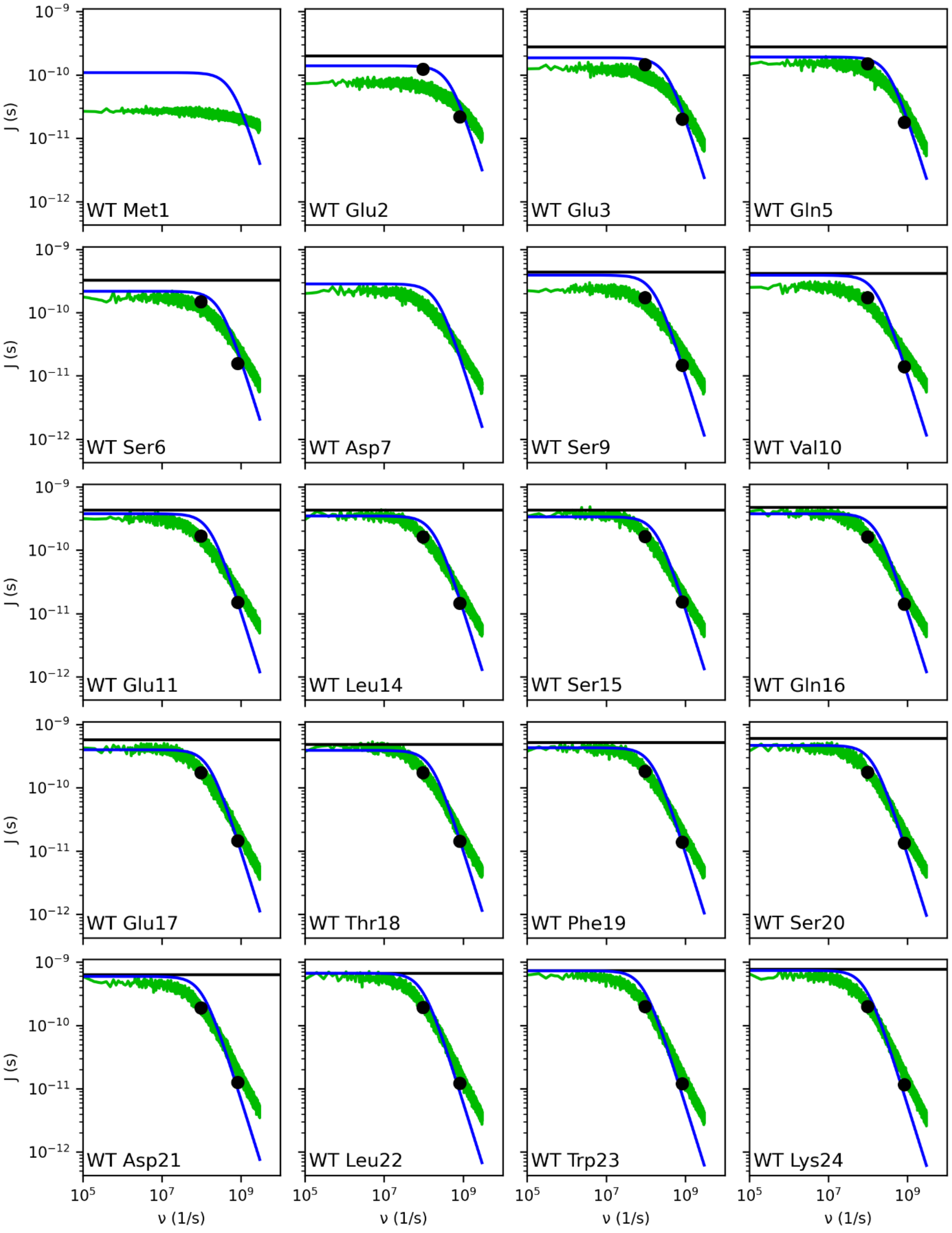

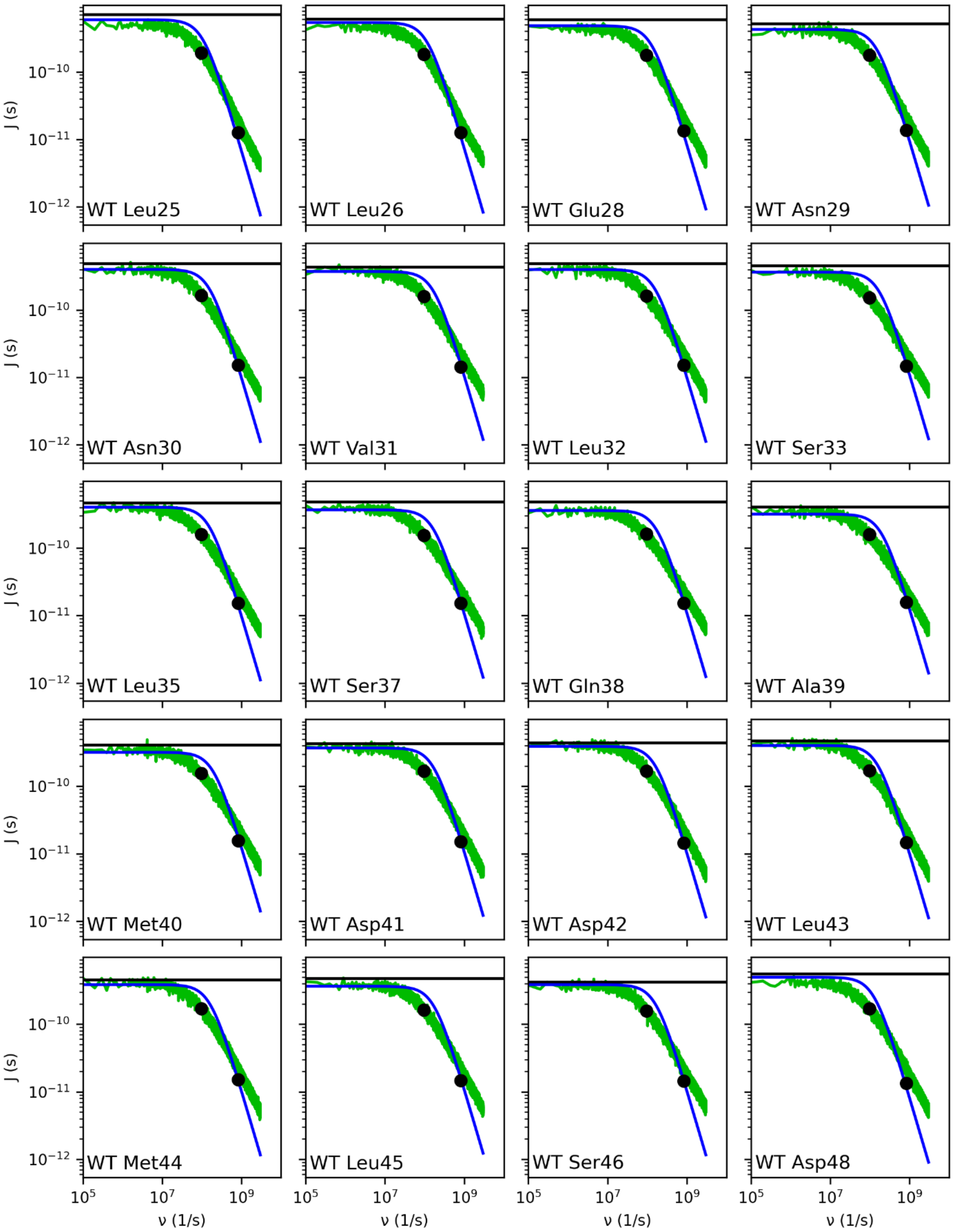
WT Leu25
WT Leu26
WT Glu28
WT Asn29
WT Asn30
WT Val31
WT Leu32
WT Ser33
WT Leu35
WT Ser37
WT Gln38
WT Ala39
WT Met40
WT Asp41
WT Asp42
WT Leu43
WT Met44
WT Leu45
WT Ser46
WT Asp48
J (s)
ν (1/s)

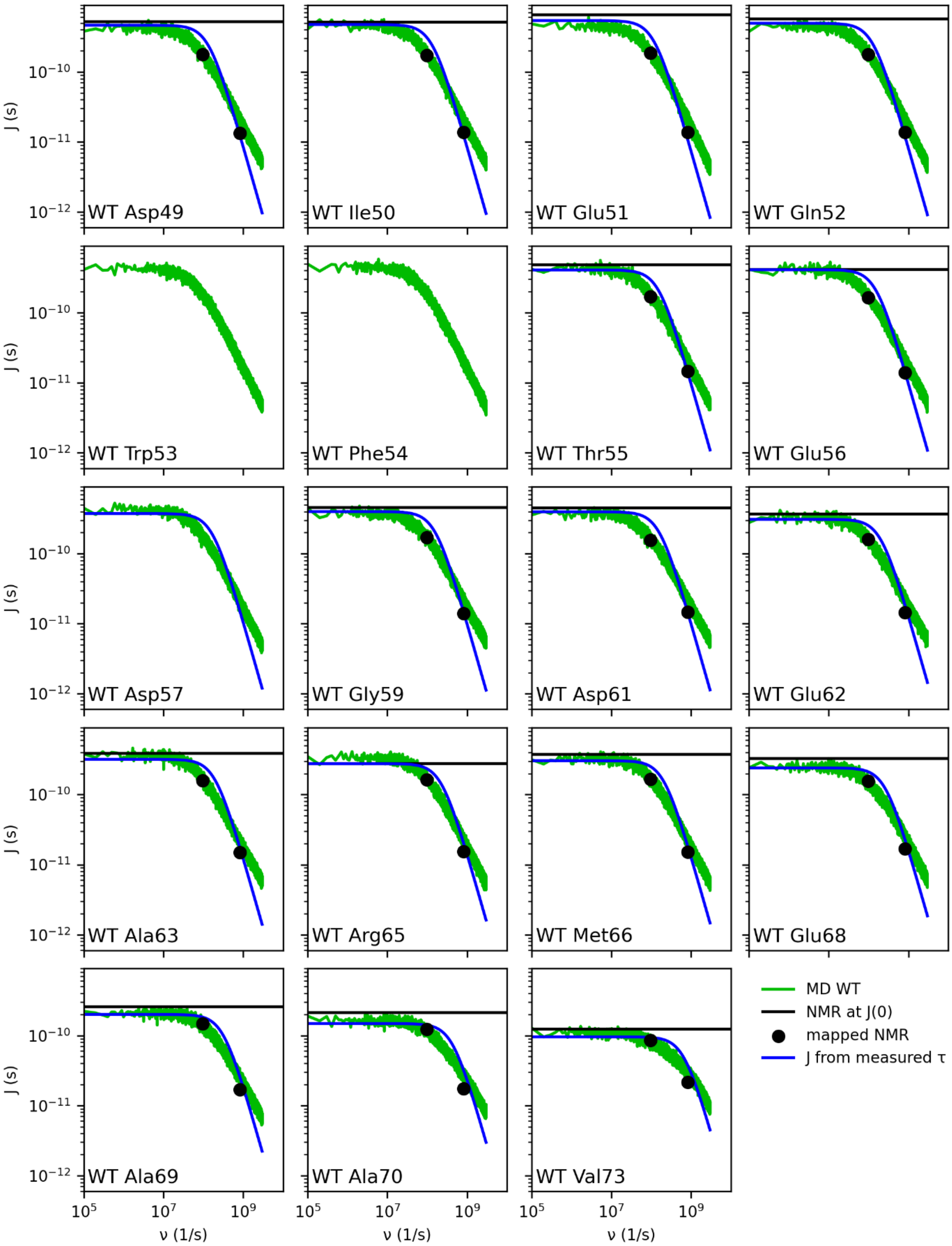

Supplementary Figure 17. Calculated SDFs vs. NMR experiment at 298 K. Shown are SDFs $J(\nu)$ calculated from our MD simulations (green lines, n=30) and measured SDF values (n=2) at frequency 0 (horizontal black lines) as well as at frequencies $\nu_1 = \omega_N/2\pi = 96.3$ MHz and $\nu_2 = \varepsilon\omega_H/2\pi = 826.5$ MHz, respectively (black dots) for each residue of the p53-TAD WT, as indicated in each panel. Blue lines show SDFs calculated from a simple single-state model, for which the relaxation time τ_c determined from TRACT experiments was used. Note that, following the convention in the literature, frequencies ν are given in units 1/s, whereas frequencies ω are in units rad/s. To facilitate easier comparison with low-frequency spectra, e.g. from CPMG experiments, and unfortunately contrary to common usage, we show high frequency spectra such as the SDF also as a function of ν and in units of 1/s.

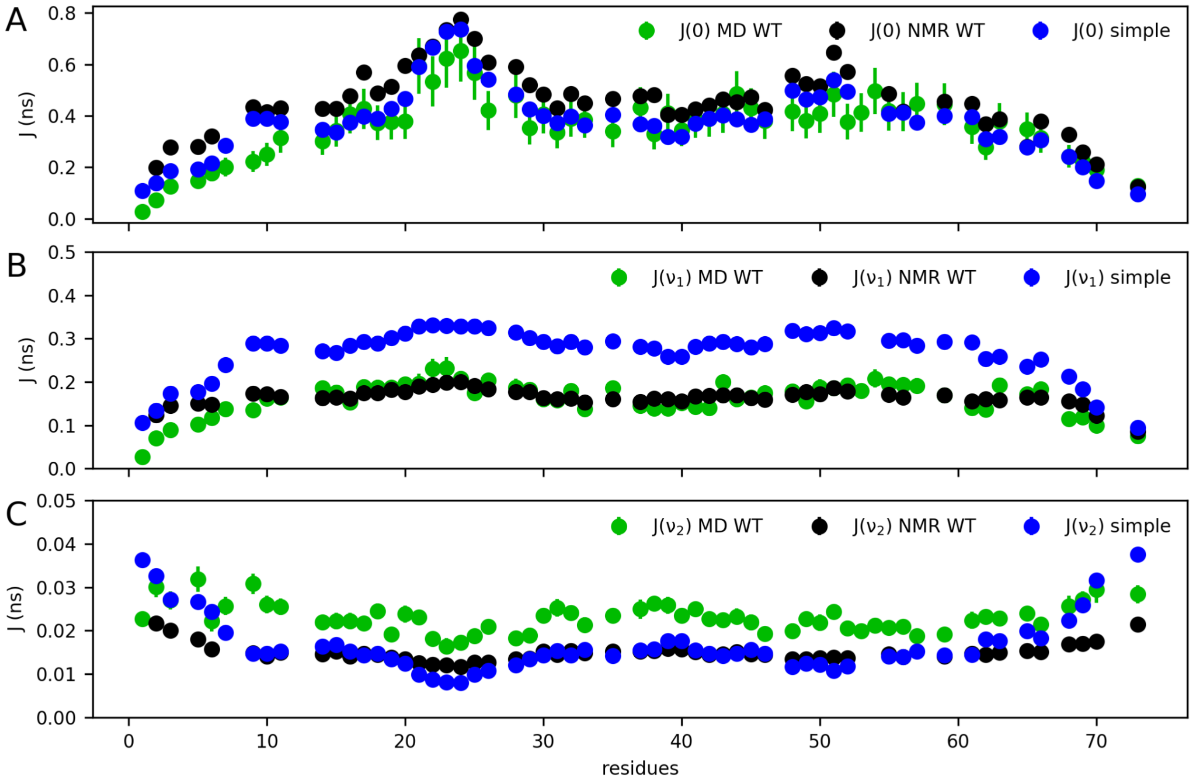

Supplementary Figure 18. Summary of calculated SDFs vs. NMR experiment at 298 K. Shown are SDFs $J(\nu)$ at the three experimentally accessible frequencies (**A**) $\nu = 0$, (**B**) $\nu_1 = \omega_N/2\pi$ = 96.3 MHz, and (**C**) $\nu_2 = \varepsilon\omega_H/2\pi$ = 826.5 MHz (cf. Supplementary Figure 17). Each panel compares SDF values calculated from our MD simulations (green, n=30) with measured ones (black, n=2) and those predicted from a simple model (blue) using measured values for τ_c from NMR. Errors are indicated as vertical bars and are otherwise smaller than the symbols.

Analyses and comparison to polymer models

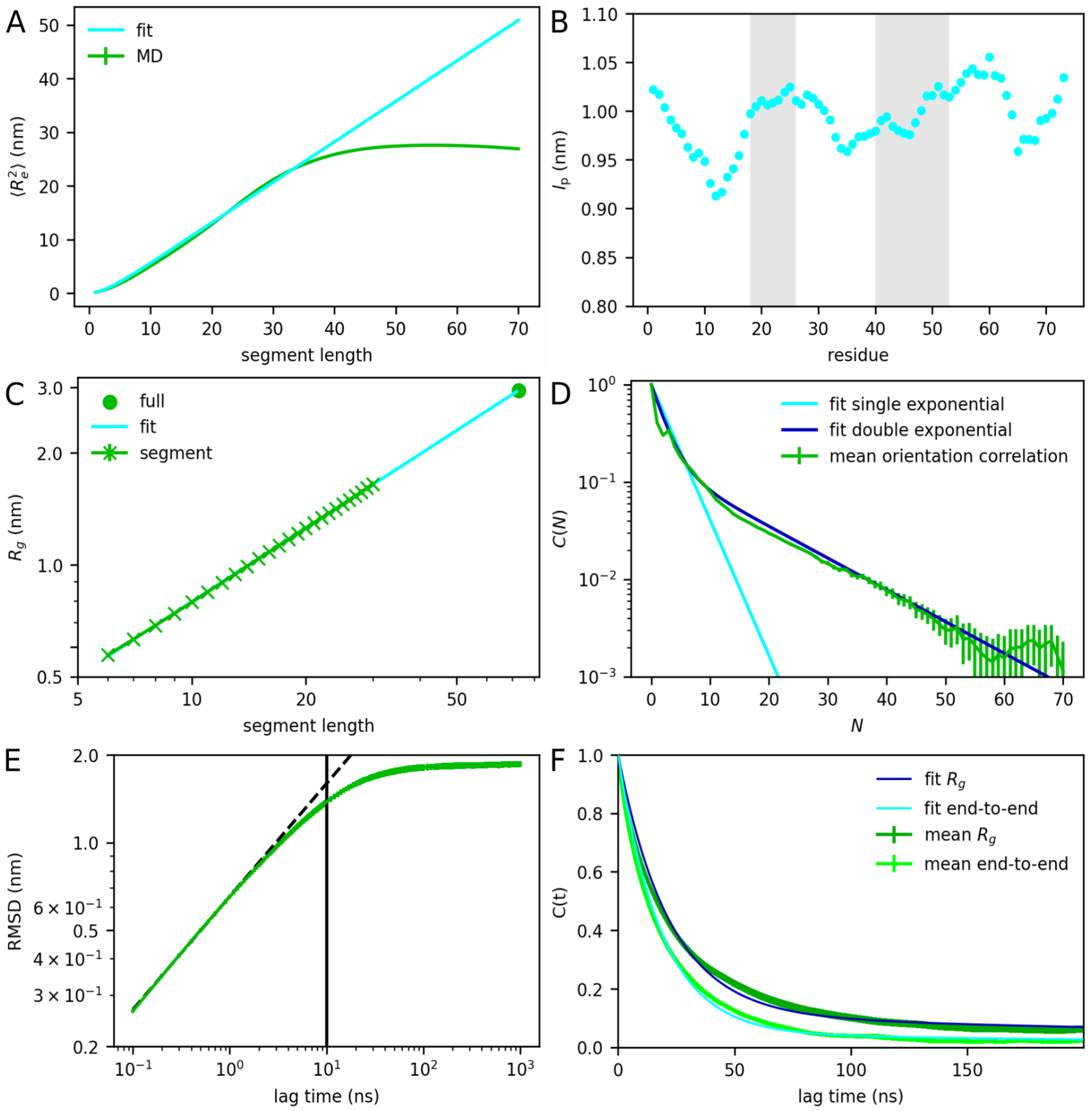

Supplementary Figure 19. Analysis of p53-TAD from a polymer physics perspective. (**A**) Mean squared end-to-end distance for increasing peptide segment length scanned over the protein (green) and a fitted scaling law function (cyan); the estimated error of the mean is smaller than the line width. (**B**) Persistence length (cyan) derived from the mean squared end-to-end distance of a fixed segment length, starting from each residue; grey areas indicate helix 1 (18-26) and helix 2 (40-54) residues. (**C**) Radius of gyration calculated for increasing protein segment lengths (green crosses) and for the full peptide (green circle); the cyan line shows a scaling law fit; the estimated standard error is smaller than the line width. (**D**) Orientation autocorrelation function of vectors of consecutive C_α atoms with increasing separation (*N*); error bars show the error of the mean; a single (cyan) and a double (blue) exponential function was fitted to obtain the persistence length. (**E**) Average increase of root mean squared deviation (RMSD) over increasing lag time; error bars show the error of the mean; the vertical black line indicates the estimated beginning of the RMSD saturation. (**F**) Time autocorrelation functions of the full p53-TAD radius of gyration (dark green) and end-to-end distance (light green); error bars show the standard error; dark blue and cyan lines show double exponential function fits. All calculations are based on 30 repeated MD trajectories of the WT sequence.

Stretching parameters from RD profiles for the Measles N_{TAIL} peptide

To test if the broad spectrum of time scales observed for p53-TAD is unique to this IDP or, rather, is a more general feature of IDPs, we have additionally calculated and analysed RD profiles from MD simulations of the measles virus peptide N_{TAIL} (residues 399-525). Compared to p53-TAD (Supplementary Figure 20), N_{TAIL} has a different charge distribution and no sequential similarity to p53-TAD. These simulations and their comparison to experiments have been described previously[30] and also indicate unexpectedly complex dynamics. For these simulations, the AMBER99SB-disp force field and the TIP4P water model[9,31] were used, with Na^+ and Cl^- ions at an ion concentration of 150 mM; six 10 µs MD simulations were performed.

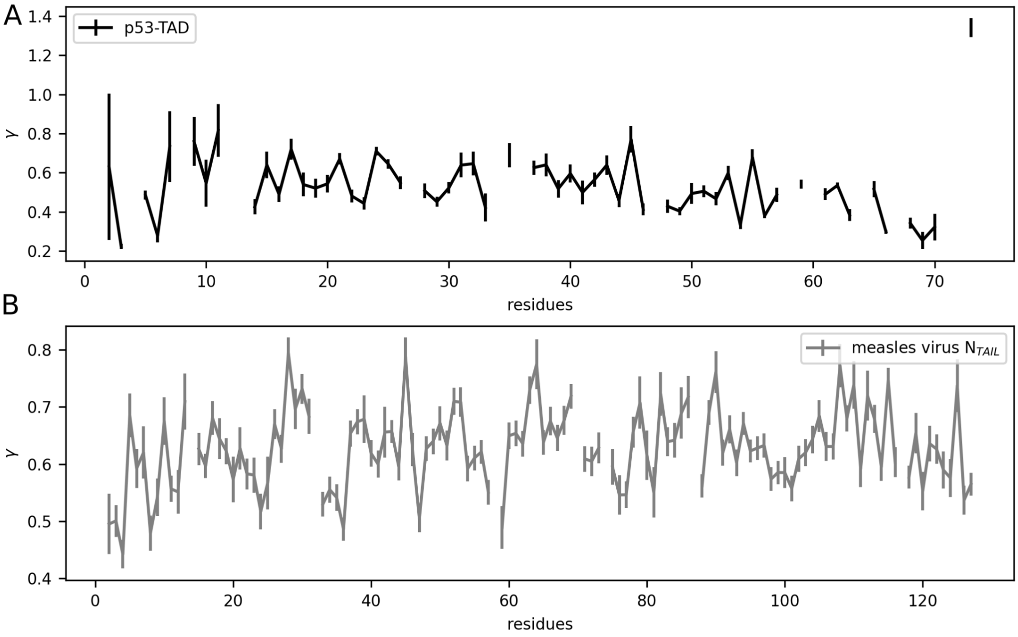

Supplementary Figure 20. Different IDPs show similar multi-timescale dynamics. Shown are, for each residue, stretching parameters γ obtained from fits of the stretched CPMG equation (equation (20), main text) to RD profiles calculated from a set of MD trajectories of (**A**) p53-TAD (n=30) and (**B**) the Measles virus N_{TAIL} (n=6). The symbols show the mean of the Bayesian posterior distributions and error bars indicate its standard deviation.

The RD profiles of N_{TAIL} (Supplementary Figure 20B) were calculated and a stretched CPMG equation (equation (20), main text) was fitted using the same procedure as described above for p53-TAD. Supplementary Figure 20 compares the resulting stretching parameters γ for each residue, which describes how much the profiles are stretched with respect to the analytical two-state CPMG profile (equation (19), main text). Here, $\gamma = 1$ corresponds to an unmodified version of the CPMG equation, and increasingly smaller values indicate more extended RD profiles and, hence, superpositions of more exchange processes with timescales distributed over an increasingly broader frequency range. As can be seen, on average both IDPs show similar γ-parameters of about 0.6, scattering over similar ranges between ca. 0.25 and 0.95 for the individual residues, with by far the most being markedly smaller than 1. This result suggests that the observed multi-timescale dynamics are rather independent of the sequence, length, and physico-chemical properties of a particular IDP, and rather a more general phenomenon. Clearly extensive MD simulations of other IDPs will be helpful to further establish this finding.

p53-TAD tertiary structures resembling protein structure elements

To test if the metastable tier 0 tertiary structures observed in our atomistic simulations also occur in protein structures, we searched the complete RCSB Protein Data Bank[32]. Specifically, we searched for protein structure fragments, the shape of which is most similar to these tier 0 tertiary structures. To this end, all 12 (WT) and 52 (P27A) stable tertiary structures detected by C_α distance fluctuations (see main text, methods) were used as a search query in "Structure Similarity Search" mode with parameters "strict_shape_match" and scoring_strategy = "structure", which identifies structures that are similar in shape (i.e. electron density overlap[33]), irrespective of sequence or specific atom types. Both complete tertiary structures as well as 11 residue long subsets sliding along the sequence (WT: 126; P27A: 460) were used for the search. For the complete structures, the best 10 search results (based on their score) were recorded; for the 11 residue long subsets, only the best matching result was recorded. Structure fragments consisting of a helix only were discarded. All identified protein structure fragments were compared to the respective metastable tier 0 tertiary structure using the software PyMOL[34]. Structure similarity was quantified using root mean squared distances (RMSDs) calculated using the PyMOL "super" method, which also accepts sequentially unrelated structures for comparison.

For the complete WT tertiary structures, four similar structure fragments were identified with RMSD values of approximately 0.3 nm; Supplementary Figure 21A shows an example. For the complete P27A tertiary structures, three fragments were identified (Supplementary Figure 21B). Using the 11 residue long sliding sub-structures, two structure fragments resembling tertiary structures seen for the WT were identified (e.g. Supplementary Figure 21C), and six different structure fragments seen for the P27A mutant. Supplementary Figure 21D shows an example of the latter with an RMSD of 0.08 nm.

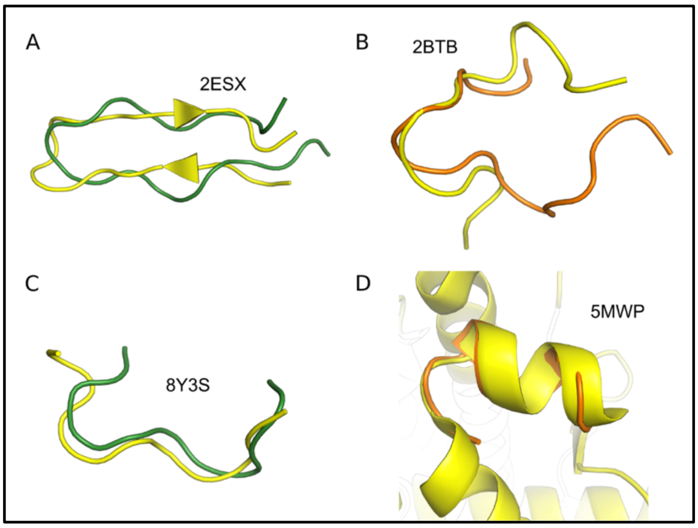

Supplementary Figure 21. Examples of transient tertiary tier 0 structures compared to similar structure fragments identified in the RCSB Protein Data Bank. (**A**) Cartoon representation of a tertiary structure observed for the p53-TAD WT (green) and structure fragment (yellow) of the V3 region of gp120 of the JR-FL HIV-1 strain with PDB-ID indicated in the figure; (**B**) P27A tertiary structure (orange) and N-terminal residues 1-15 of the human band 3 peptide (yellow); (**C**) p53-TAD WT tertiary structure (green) and residues 53-63 of the human keratin 19 head domain (yellow); (**D**) P27A tertiary structure (orange) and residues 22-32 of the human mineralocorticoid receptor (yellow).

Supplementary References

1 Chodera, J. D. & Noé, F. Markov state models of biomolecular conformational dynamics. *Curr. Opin. Struct. Biol.* **25**, 135-144 (2014). https://doi.org:10.1016/j.sbi.2014.04.002

2 Koss, H., Rance, M. & Palmer, A. G. Algebraic expressions for Carr-Purcell-Meiboom-Gill relaxation dispersion for N-site chemical exchange. *J. Magn. Reson.* **321**, 106846 (2020). https://doi.org:10.1016/j.jmr.2020.106846

3 Grey, M. J., Wang, C. & Palmer, A. G. Disulfide bond isomerization in basic pancreatic trypsin inhibitor: Multisite chemical exchange quantified by CPMG relaxation dispersion and chemical shift modeling. *J. Am. Chem. Soc.* **125**, 14324-14335 (2003). https://doi.org:10.1021/ja0367389

4 Salvi, N., Abyzov, A. & Blackledge, M. Multi-timescale dynamics in intrinsically disordered proteins from NMR relaxation and molecular simulation. *J. Phys. Chem. Lett.* **7**, 2483-2489 (2016). https://doi.org:10.1021/acs.jpclett.6b00885

5 Shen, Y. & Bax, A. SPARTA+: a modest improvement in empirical NMR chemical shift prediction by means of an artificial neural network. *J. Biomol. NMR* **48**, 13-22 (2010). https://doi.org:10.1007/s10858-010-9433-9

6 Han, B., Liu, Y., Ginzinger, S. W. & Wishart, D. S. SHIFTX2: significantly improved protein chemical shift prediction. *J. Biomol. NMR* **50**, 43-57 (2011). https://doi.org:10.1007/s10858-011-9478-4

7 Wong, T. R., Trevor; Freund, Stefan; Fersht, Alan. BMRB Entry 17760: Backbone resonance assignments of p53 N-terminal transactivation domain (1-93) https://bmrb.io/data_library/summary/?bmrbId=17760. (2011).

8 Rauscher, S. *et al.* Structural ensembles of intrinsically disordered proteins depend strongly on force field: A comparison to experiment. *J. Chem. Theory Comput.* **11**, 5513-5524 (2015). https://doi.org:10.1021/acs.jctc.5b00736

9 Robustelli, P., Piana, S. & Shaw, D. E. Developing a molecular dynamics force field for both folded and disordered protein states. *Proc. Natl. Acad. Sci. U.S.A.* **115**, E4758-E4766 (2018). https://doi.org:10.1073/pnas.1800690115

10 Huang, J. *et al.* CHARMM36m: an improved force field for folded and intrinsically disordered proteins. *Nat. Methods* **14**, 71-73 (2017). https://doi.org:10.1038/nmeth.4067

11 Best, R. B. & Hummer, G. Optimized molecular dynamics force fields applied to the helix-coil transition of polypeptides. *J. Phys. Chem. B* **113**, 9004-9015 (2009). https://doi.org:10.1021/jp901540t

12 Abascal, J. L. F. & Vega, C. A general purpose model for the condensed phases of water: TIP4P/2005. *J. Chem. Phys.* **123** (2005). https://doi.org:10.1063/1.2121687

13 Izadi, S., Anandakrishnan, R. & Onufriev, A. V. Building water models: A different approach. *J. Phys. Chem. Lett.* **5**, 3863-3871 (2014). https://doi.org:10.1021/jz501780a

14 Nygaard, M., Kragelund, B. B., Papaleo, E. & Lindorff-Larsen, K. An efficient method for estimating the hydrodynamic radius of disordered protein conformations. *Biophys. J.* **113**, 550-557 (2017). https://doi.org:10.1016/j.bpj.2017.06.042

15 Kirkwood, J. G. The general theory of irreversible processes in solutions of macromolecules. *J. Polym Sci.* **12**, 1-14 (1954). https://doi.org:10.1002/pol.1954.120120102

16 Ortega, A., Amorós, D. & García de la Torre, J. Prediction of hydrodynamic and other solution properties of rigid proteins from atomic- and residue-level models. *Biophys. J.* **101**, 892-898 (2011). https://doi.org:10.1016/j.bpj.2011.06.046

17 Sutherland, W. *LXXV. A dynamical theory of diffusion for non-electrolytes and the molecular mass of albumin*. Vol. 9 (Taylor & Francis, 1905).

18 Nagy, G., Hoffmann, S. V., Jones, N. C. & Grubmüller, H. Reference data set for circular dichroism spectroscopy comprised of validated intrinsically disordered protein models. *Appl. Spectrosc.* **78**, 897-911 (2024). https://doi.org:10.1177/00037028241239977
19 Daughdrill, G. W. *et al.* Understanding the structural ensembles of a highly extended disordered protein. *Mol. Biosyst.* **8**, 308-319 (2012). https://doi.org:10.1039/C1MB05243H
20 Franke, D. *et al.* ATSAS 2.8: a comprehensive data analysis suite for small-angle scattering from macromolecular solutions. *J. Appl. Crystallogr.* **50**, 1212-1225 (2017). https://doi.org:10.1107/S1600576717007786
21 Svergun, D., Barberato, C. & Koch, M. H. J. CRYSOL – a program to evaluate X-ray solution scattering of biological macromolecules from atomic coordinates. *J. Appl. Crystallogr.* **28**, 768-773 (1995). https://doi.org:10.1107/S0021889895007047
22 dos Remedios, C. G., Miki, M. & Barden, J. A. Fluorescence resonance energy transfer measurements of distances in actin and myosin. A critical evaluation. *J. Muscle Res. Cell Motil.* **8**, 97-117 (1987). https://doi.org:10.1007/BF01753986
23 Huang, F. *et al.* Multiple conformations of full-length p53 detected with single-molecule fluorescence resonance energy transfer. *Proc. Natl. Acad. Sci. U.S.A.* **106**, 20758-20763 (2009). https://doi.org:10.1073/pnas.0909644106
24 Moses, D. *et al.* Structural biases in disordered proteins are prevalent in the cell. *Nat. Struct. Mol. Biol* **31**, 283-292 (2024). https://doi.org:10.1038/s41594-023-01148-8
25 Lum, J. K., Neuweiler, H. & Fersht, A. R. Long-range modulation of chain motions within the intrinsically disordered transactivation domain of tumor suppressor p53. *J. Am. Chem. Soc.* **134**, 1617-1622 (2012). https://doi.org:10.1021/ja2078619
26 Clore, G. M. & Iwahara, J. Theory, practice, and applications of paramagnetic relaxation enhancement for the characterization of transient low-population states of biological macromolecules and their complexes. *Chem. Rev.* **109**, 4108-4139 (2009). https://doi.org:10.1021/cr900033p
27 Lowry, D. F., Stancik, A., Shrestha, R. M. & Daughdrill, G. W. Modeling the accessible conformations of the intrinsically unstructured transactivation domain of p53. *Proteins: Struct. Funct. Bioinf.* **71**, 587-598 (2008). https://doi.org:10.1002/prot.21721
28 Liu, X. & Chen, J. Residual structures and transient long-range interactions of p53 transactivation domain: Assessment of explicit solvent protein force fields. *J. Chem. Theory Comput.* **15**, 4708-4720 (2019). https://doi.org:10.1021/acs.jctc.9b00397
29 Tesei, G. *et al.* DEER-PREdict: Software for efficient calculation of spin-labeling EPR and NMR data from conformational ensembles. *PLoS Comput. Biol.* **17**, e1008551 (2021). https://doi.org:10.1371/journal.pcbi.1008551
30 Otteson, L. *et al.* Transient non-local interactions dominate the dynamics of measles virus N-TAIL. *Commun. Chem.* **8**, 298 (2025). https://doi.org:10.1038/s42004-025-01682-0
31 Piana, S., Donchev, A. G., Robustelli, P. & Shaw, D. E. Water dispersion interactions strongly influence simulated structural properties of disordered protein states. *J. Phys. Chem. B* **119**, 5113-5123 (2015). https://doi.org:10.1021/jp508971m
32 Berman, H. M. *et al.* The Protein Data Bank. *Nucleic Acids Res.* **28**, 235-242 (2000). https://doi.org:10.1093/nar/28.1.235
33 Guzenko, D., Burley, S. K. & Duarte, J. M. Real time structural search of the Protein Data Bank. *PLoS Comput. Biol.* **16**, e1007970 (2020). https://doi.org:10.1371/journal.pcbi.1007970
34 The PyMOL Molecular Graphics System, Version 3.9 Schrödinger, LCC (2024).
