# Supplementary material for: Hierarchical multi-timescale structural dynamics of the disordered N-terminal of p53
Source: Nat Commun. 2026 May 21;17:4812. doi: 10.1038/s41467-026-73145-6 (PMC13221463; doi:10.1038/s41467-026-73145-6)
Supplement: Supplementary file 2 — Description of Additional Supplementary Files [file 41467_2026_73145_MOESM2_ESM.pdf]

Description of Additional Supplementary Files

Supplementary Movie 1: Super slow-motion movie of a sample MD simulation trajectory of the p53 P27A mutant. Shown is the trace of the backbone (grey), with helix1 highlighted in cyan, helix 2 in orange, and the N-terminal in yellow. A total of 100 ns is shown (2.5 ns/second).

Supplementary Movie 2: Slow motion movie of a sample MD simulation trajectory of the p53 P27A mutant. Coloring as in 1; a total of 3 µs is shown (25 ns/second).

Supplementary Movie 3: Movie of a sample MD simulation trajectory of the p53 P27A mutant. Coloring as in 1; a total of 10 µs is shown in slow motion (250 ns/second).
